# Ambient Aqueous Synthesis of Imine-Linked Covalent Organic Frameworks (COFs) and Fabrication of Freestanding Cellulose Nanofiber@COF Nanopapers

**DOI:** 10.1021/jacs.3c10691

**Published:** 2023-12-19

**Authors:** Xueying Kong, Zhongqi Wu, Maria Strømme, Chao Xu

**Affiliations:** † Division of Nanotechnology and Functional Materials, Department of Materials Science and Engineering, Uppsala University, Uppsala SE-75121, Sweden; ‡ Institute of Molecular Engineering and Applied Chemistry, Anhui University of Technology, Ma’anshan 243002, P. R. China

## Abstract

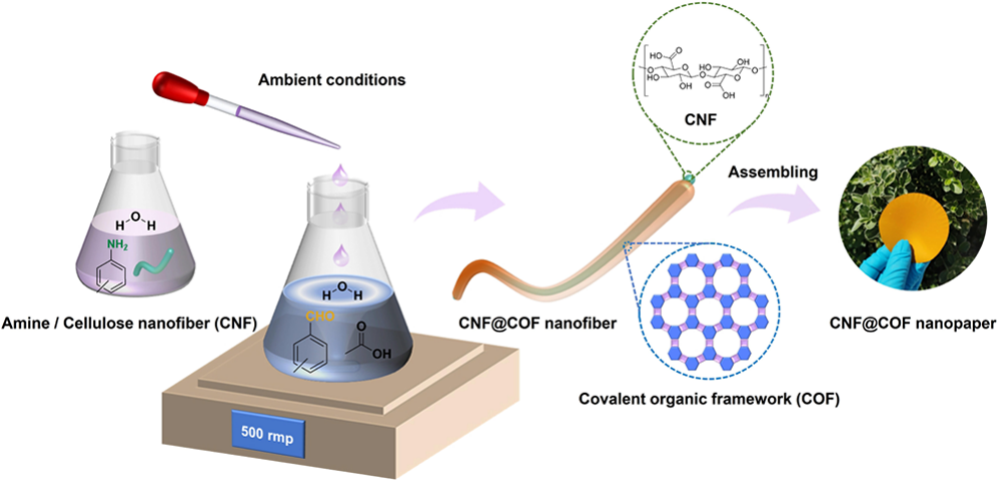

Covalent organic frameworks (COFs) are usually synthesized under solvothermal conditions that require the use of toxic organic solvents, high reaction temperatures, and complicated procedures. Additionally, their insolubility and infusibility present substantial challenges in the processing of COFs. Herein, we report a facile, green approach for the synthesis of imine-linked COFs in an aqueous solution at room temperature. The key behind the synthesis is the regulation of the reaction rate. The preactivation of aldehyde monomers using acetic acid significantly enhances their reactivity in aqueous solutions. Meanwhile, the still somewhat lower imine formation rate and higher imine breaking rates in aqueous solution, in contrast to conventional solvothermal synthesis, allow for the modulation of the reaction equilibrium and the crystallization of the products. As a result, highly crystalline COFs with large surface areas can be formed in relatively high yields in a few minutes. In total, 16 COFs are successfully synthesized from monomers with different molecular sizes, geometries, pendant groups, and core structures, demonstrating the versatility of this approach. Notably, this method works well on the gram scale synthesis of COFs. Furthermore, the aqueous synthesis facilitates the interfacial growth of COF nanolayers on the surface of cellulose nanofibers (CNFs). The resulting CNF@COF hybrid nanofibers can be easily processed into freestanding nanopapers, demonstrating high efficiency in removing trace amounts of antibiotics from wastewater. This study provides a route to the green synthesis and processing of various COFs, paving the way for practical applications in diverse fields.

## Introduction

Covalent organic frameworks (COFs), a novel class of porous crystalline materials, are constructed by linking organic building blocks through covalent bonds.^1−3^ COFs have predesigned structures, high chemical and thermal stabilities, high porosity and ordered porous channels, which has led them being widely studied in adsorption,^4−6^ separation,^7−10^ sensing,^11,12^ catalysis,^13,14^ drug delivery,^15,16^ and proton conduction^17,18^ applications. In recent years, solvothermal,^1,19,20^ sonochemical,^21,22^ microwave,^23^ and mechanochemical^24^ syntheses have been developed to fabricate a variety of COFs. Of the aforementioned approaches, solvothermal reactions are the most commonly used. Solvothermal COF synthesis usually involves harsh experimental conditions (e.g., toxic organic solvents, high reaction temperatures, long reaction times, and appropriate pressure) and complicated procedures (e.g., freeze–pump–thaw cycles, flame sealing of tubes, heating for several days). The use of toxic organic solvents, in particular, is economically and environmentally costly. Meanwhile, the complicated procedures have significantly limited the large-scale synthesis of COFs. Therefore, there is great interest in exploring facile, scalable, and environmentally friendly methods for COF synthesis.

Schiff-base condensation reactions between amines and aldehydes or ketones, to create imine bonds, have been widely employed in the construction of porous organic materials,^25^ such as imine-linked porous organic polymers^26,27^ and COFs.^28,29^ Interestingly, imines can be synthesized in aqueous or organic solvents (or even by solid-state synthesis), which means Schiff-base reactions are ideally suited to be used in environmentally friendly COF synthesis. Banerjee and co-workers have developed a simple approach for synthesizing a series of highly crystalline COFs;^30^ they do so by baking organic linkers (2,4,6-trimethoxy-1,3,5-benzenetricarbaldehyde and different diamines) with a *p*-toluene sulfonic acid (PTSA) catalyst and small amounts of water as the solvent. This study was a milestone in the development of COF materials, offering a simple and easy COF synthesis procedure. However, the large quantity of PTSA required incurs an environmental cost. Zamora and co-workers have recently reported the synthesis of imine-linked COFs in aqueous solutions with acetic acid.^31^ Although this approach avoided the use of organic solvents, the reactions required a very low concentration of the reactants due to the low aqueous solubility of the organic monomers that would produce a large amount of wastewater when it comes to large-scale synthesis. High reaction temperatures and relatively long reaction times (5 days) were still required for COF crystallization by this method. Recently, Cooper and co-workers have developed a probe sonochemical synthesis method for imine-linked COFs in aqueous solutions.^22^ The high-energy ultrasound significantly facilitated the dissolution and dispersion of the organic monomers in water and, thus, increased their reactivity. By sonication of the starting monomers in an aqueous acetic acid solution at room temperature for 1 h, a large family of COFs with high crystallinity were synthesized. However, the practical application of probe sonochemical synthesis remains a challenge.

On the other hand, COFs are typically synthesized in the form of insoluble and infusible powders, which present considerable difficulties when attempting to shape them into desired forms and structures for practical applications.^32,33^ Recently, the emergence of liquid–liquid and liquid–air interface polymerization methods has allowed for the successful production of freestanding thin COF films. However, this approach encounters obstacles in terms of scalability, such as the use of a substantial quantity of organic solvents, and the limited mechanical strength of COF films.^9,10,34−36^ To overcome these processing challenges, an alternative approach involves integrating COFs with specific substrates to create composite materials. For instance, blending as-synthesized COF particles with polymers to form mixed matrix membranes (MMMs) represents a straightforward method for COF processing. Nonetheless, this approach can result in the entrapment of COF particles within the matrix, partially blocking their porous channels due to the presence of blending agents.^37−39^ Interfacial synthesis of COFs on specific substrates (e.g., porous oxides, polymer membranes, electrospun polyacrylonitrile nanofibers) offers a solution by allowing for the formation of COF layers on surfaces and the subsequent fabrication of freestanding composites.^7,40,41^ Nevertheless, the challenge remains in fabricating freestanding COF composites with high mechanical strength and flexibility by using environmentally friendly methods.

In this study, we present an easy and environmentally friendly approach to synthesizing imine-linked COFs. By stirring the starting organic monomers in water and acetic acid, at room temperature, highly crystalline and porous COFs can be synthesized in a very short reaction time (up to 1 min). The key to this procedure lies in the preactivation of the aldehyde monomer using acetic acid, which significantly enhances its reactivity in water. The strategy demonstrates remarkable generality, as evidenced by the successful synthesis of 16 distinct COFs using this method. Moreover, we have achieved the formation of COF nanolayers on cellulose nanofibers (CNFs) through interfacial synthesis in an aqueous solution. The resulting COF@CNF hybrid nanofibers can be fabricated into freestanding and flexible nanopapers.

## Results and Discussion

**Scheme 1:**
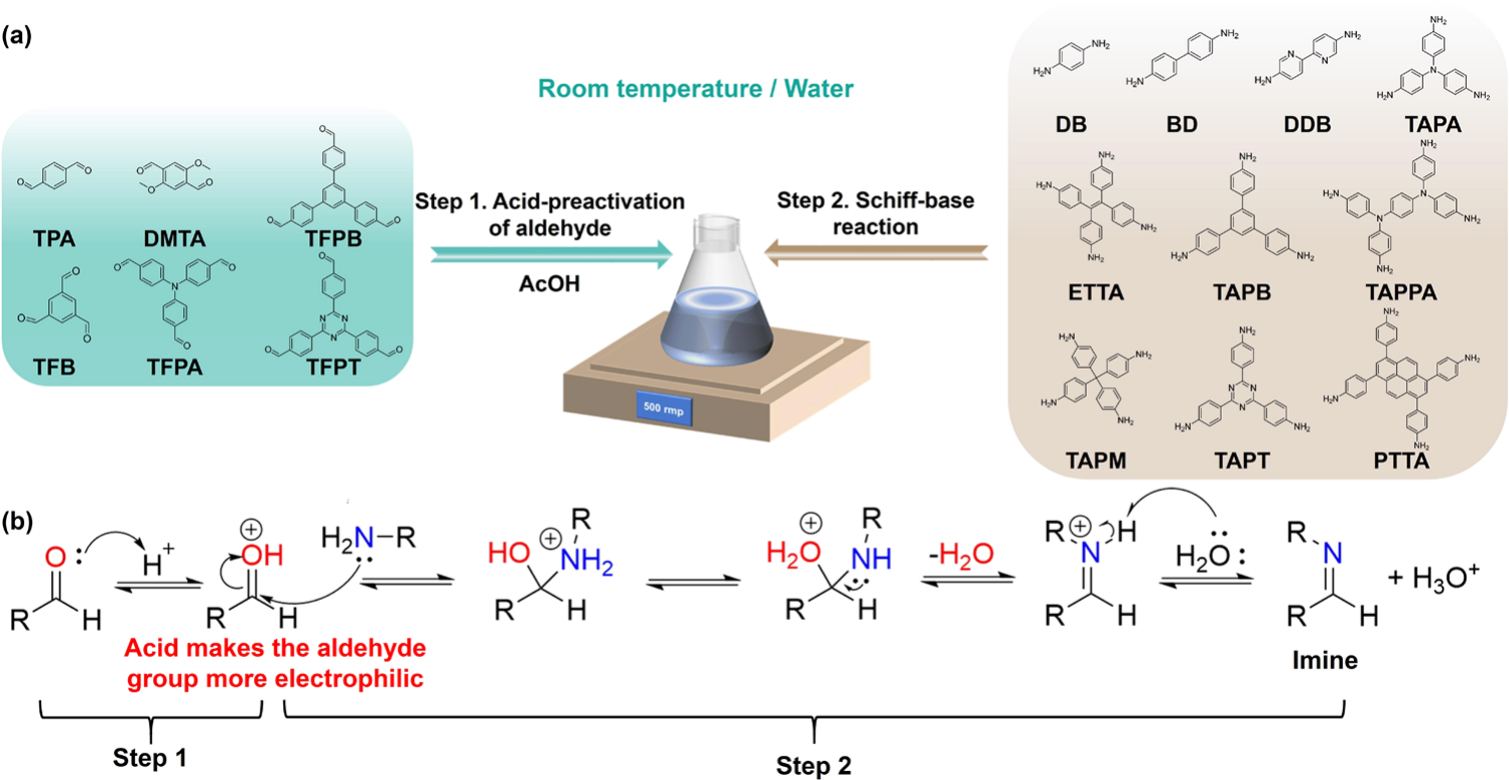
(a) Schematic Synthesis of Imine-Linked COFs in Aqueous Solution. (b) Formation Process of Imine through Nucleophilic Addition of Amine on Acid-Preactivated Aldehyde^42^

The use of acid catalysts is crucial for Schiff-base condensation reactions and the formation of imine-linked COFs. Schiff-base condensations involve a nucleophilic attack of the aldehyde by the amine, to form a hemiaminal intermediate, followed by the dehydration of the hemiaminal to produce the imine.^42^ Acid plays different roles in two steps: the presence of acid hinders the first step by protonating the amine, while acid must be present in the second step for the protonation of the OH leaving group, which allows the formation of an imine by dehydrating the hemiaminal. Therefore, the acidity of the reaction mixture must be carefully adjusted to compensate for these competing processes. If the pH is either too high or too low, the COF synthesis will not proceed.^31,43^ Unlike the protonation of the amine, which decreases its nucleophilicity and thereby slows the nucleophilic addition reaction, an acid could make the aldehyde group more electrophilic. For instance, Jiang et al. conducted a study in which they observed an increase in the Fukui function for nucleophilic attack sites on an aldehyde monomer from 0.066 to 0.152 upon acid activation. This suggests that the acid-activated aldehyde is more liable to nucleophilic attack.^44^ We therefore utilized the fact that reacting the amine monomer with acid-preactivated aldehyde monomers would circumvent the decrease in the amine nucleophilicity and simultaneously increase the electrophilicity of the aldehyde. This strategy allows us to overcome the low aqueous solubility of the organic monomers, and their associated low reactivity, which makes the development of efficient approaches for aqueous synthesis of COF possible (Scheme 1).

**Figure 1:**
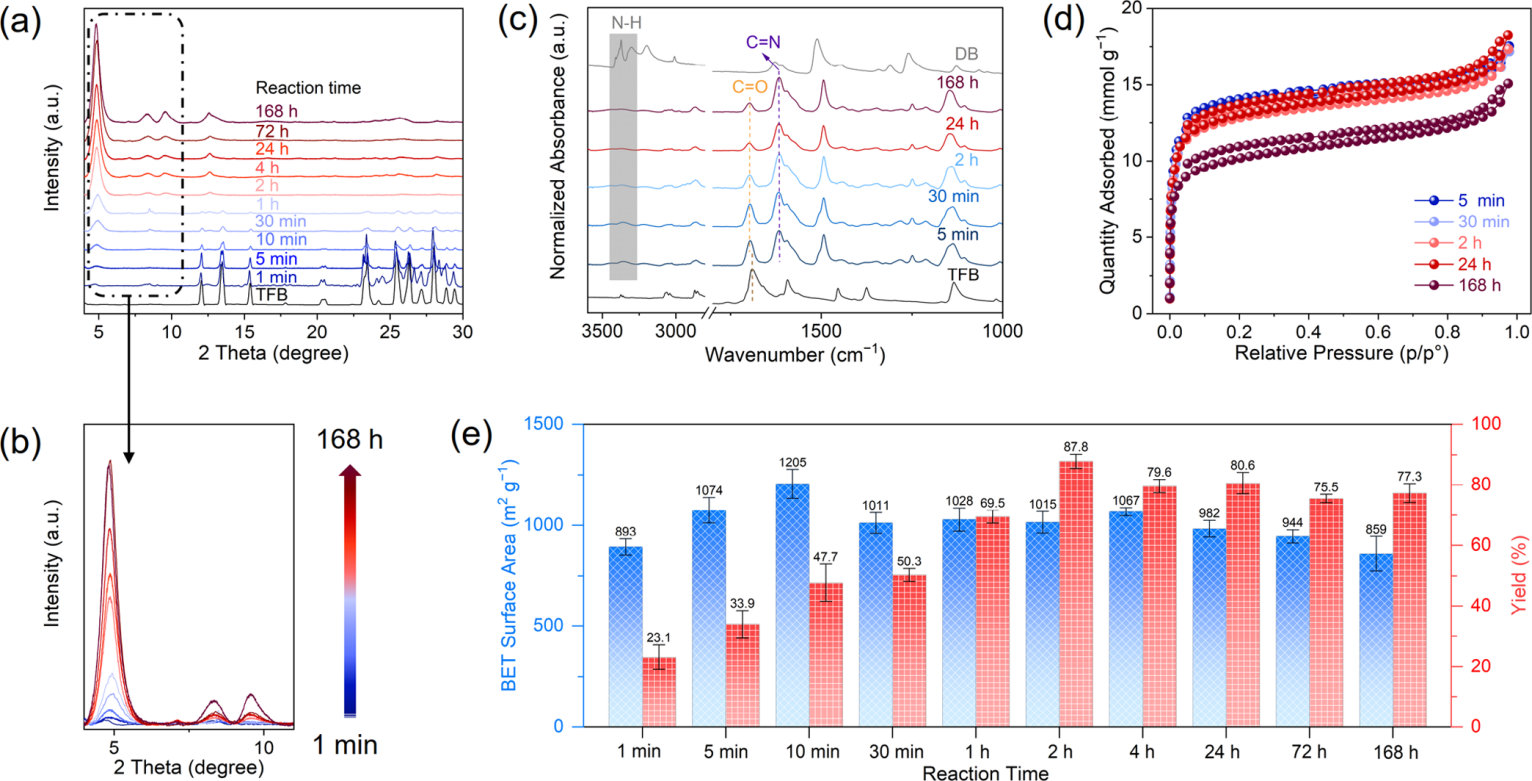
(a, b) X-ray diffraction patterns of the crude TFB-DB COF samples collected at different reaction intervals (from 1 min to 168 h). (c) Infrared spectra of the purified TFB-DB COF samples collected at different reaction intervals. (d) N_2_ adsorption–desorption isotherms of the purified TFB-DB COF samples collected at reaction intervals of 5, 30 min, 2, 24, and 168 h. (e) Comparison of the yield and BET surface area of TFB-DB COFs after different reaction times. All of the crude products were washed with water to remove residual acetic acid and unreacted DB monomer prior to the measurements. The purified samples were washed with both water and acetone to remove any acetic acid and unreacted monomers prior to the measurements.

In order to test our hypothesis, we performed a range of reactions for the synthesis of TFB-DB COF (TFB = 1,3,5-triformylbenzene, DB = 1,4-diaminebenzene) in aqueous solutions at room temperature. TFB-DB COF, also known as COF-LZU1, is a widely studied material for separation,^7^ catalysis,^13^ and energy storage^45^ applications. First, an attempt to synthesize TFB-DB COF was made by reacting aqueous DB solution with an acid-preactivated TFB suspension in water. (Note: this synthesis method will be described as AP—aldehyde preactivation—in the following discussions). Specifically, TFB powder (104 mg) was dispersed in 2 mL of water before 2 mL of glacial acetic acid was added to the mixture and stirred for ∼30 min. TFB powder is hydrophobic and its solubility in water was relatively low (Figure S1a). Adding acetic acid significantly facilitated the dispersion of the powder in water, resulting in a homogeneous suspension (Figure S1b), and this is caused by the protonation of the aldehyde group increasing the hydrophilicity and aqueous solubility of the monomer (Figure S2). A clear, brown aqueous solution of DB monomer (4 mL, 0.24 M) was added dropwise into the TFB suspension with stirring at room temperature, and a large amount of orange precipitate formed immediately (Figure S1c,d). Aliquots were taken from the suspension at time intervals of 1, 5, 10, 30 min, 1, 2, 4, 24, 72, and 168 h, and the solids collected were analyzed by powder X-ray diffraction (XRD), with the aim of monitoring the reaction by analyzing the structural evolution of the product over time. The collected samples were washed with water to remove the acetic acid and any unreacted DB monomer prior to the XRD measurements. Remarkably, the characteristic TFB-DB COF peak at 2θ ≈ 4.8° (corresponding to the (100) plane of TFB-DB COF) was observed in the samples after 1 min of reacting (Figure 1a,b).^13^ However, the polymerization reaction was not fully completed at this point since sharp diffraction peaks attributable to TFB monomer were observed. The relative intensity ratio of the TFB-DB COF and TFB diffraction peaks gradually increased with increasing reaction times, indicating an increasing degree of polymerization and yield of TFB-DB COF. In addition, the diffraction peaks of TFB disappeared in the samples collected after 2 h, and only diffraction peaks of TFB-DB COF could be observed, indicating that the TFB monomer was completely consumed within 2 h. No significant change was observed in the XRD patterns for the samples collected at intervals exceeding 2 h, suggesting that the structure of TFB-DB COF remained unchanged in the solution over these times.

After washing with both water and acetone, as well as subsequent Soxhlet extraction using acetone, all of the purified TFB-DB COFs obtained at various reaction intervals (ranging from 1 min to 168 h) displayed high crystallinity, as revealed by XRD analysis (Figure S4). Notably, these samples exited Brunauer–Emmett–Teller (BET)-specific surface areas ranging from 859 to 1205 m^2^ g^–1^ (Figure 1d,e), significantly surpassing those of TFB-DB COF samples synthesized through conventional solvothermal methods.^13^ The pore size distribution analysis of the adsorption isotherms indicated that the COF samples had narrow pores with sizes centered between 1.2 and 1.5 nm, in agreement with other reported samples (Figure S6). Surprisingly, a short reaction time of 1 min gave a relatively high yield of 23.1%. The yield significantly increased up to a peak value of 87.8% after 2 h and stabilized at 80.6–75.5% over the extended reaction times (4–168 h, Figure 1e). These results were consistent with results from time-dependent XRD and infrared (IR) studies (Figure 1a–c) showing that most of the monomers were polymerized within 2 h. It should be noted that longer reaction times (4–168 h) slightly decreased both the surface area and the yield of the product, which can be attributed to the reversibility of imine bonds that the large amount of water present in the reaction mixture resulted in hydrolysis of the imine products (Figures 1e and S5). It can therefore be concluded that TFB-DB COFs were successfully synthesized with a high surface area and a high yield, in water, at room temperature with a relatively short reaction time of 2 h. The short reaction time results in potentially high space–time yields of up to 62.1 and 9.5 g h^–1^ L^–1^ for the 10 min and 2 h reactions, respectively. These values are significantly higher than those reported for other aqueous synthesis methods for COFs (Table S4).

**Figure 2:**
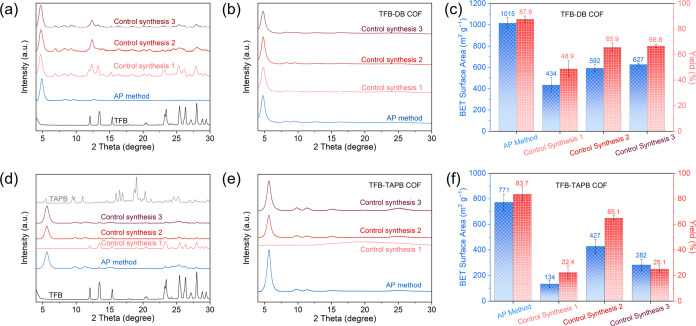
Comparison of the COFs synthesized by the AP method and the control conditions 1–3, all after 2 h of reacting: (a) X-ray diffraction (XRD) patterns of the crude TFB-DB COF; (b) XRD patterns of the purified TFB-DB COF; (c) the yield and BET surface area of TFB-DB COF; (d) XRD of the crude TFB-TAPB COF; (e) XRD of the purified TFB-TAPB COF; and (f) yield and BET surface area of the TFB-TAPB COF. All of the crude products were washed with water to remove residual acetic acid prior to the measurements. The purified samples were washed with both water and acetone to remove any acetic acid and unreacted monomers prior to the measurements.

We next designed a series of control experiments to investigate the influence of the addition of acetic acid at different reaction stages on the COF synthesis. Control synthesis 1: the amine monomer was mixed with aqueous acetic acid solution and then added to an aqueous suspension of the aldehyde monomer. Control synthesis 2: the amine and aldehyde monomers were each added acetic acid separately and then mixed together. Control synthesis 3: an aqueous solution of amine was added to an aqueous suspension of aldehyde, followed by the addition of acetic acid. The final concentration of acetic acid in all mixtures was 4.38 M. All mixtures were stirred at room temperature. In all of these reactions, a dark yellow precipitate was formed at the start of the reaction, in contrast to the orange TFB-DB COF precipitate formed by the AP synthesis method (Figure S7). The crude products collected (after washing with water) from these control syntheses after 10 min displayed strong diffraction peaks for the TFB monomer, with no significant diffraction peaks from TFB-DB COF, indicating a low yield and a low degree of crystallinity of the products. In contrast, the crude product obtained from the AP synthesis clearly showed the characteristic diffraction peak at 4.8° of the (001) plane of TFB-DB COF (Figure S8a). When the reaction time was extended to 2 h, the products of all of the control experiments displayed strong diffraction peaks characteristic of TFB-DB COF (Figure 2a). However, diffraction peaks for the TFB monomer remained in the XRD spectra of the samples obtained from the control syntheses; no such peaks were observed in the samples collected from the AP synthesis (Figure 2a). The AP synthesis method gave relatively high yields of TFB-DB COF (43.3% at 10 min, 87.8% at 2 h), significantly higher than the yields of control synthesis 1 (8.1% at 10 min, 48.9% at 2 h), control synthesis 2 (20.2% at 10 min, 65.9% at 2 h), and control synthesis 3 (18.5% at 10 min, 66.8% at 2 h) (Figure S8d). The pretreatment of the amine monomer with acetic acid (control synthesis 1) resulted in the lowest yield of all of the control experiments; this is attributed to the significantly decreased nucleophilicity and reactivity of the amine upon protonation. When both TFB and DB monomers were treated with acid (control syntheses 2 and 3), the decreased nucleophilicity of amine probably is somewhat compensated for by the increased electrophilicity of the aldehyde. This would explain why the yields of controls 2 and 3 are so similar, higher than control 1, but still lower than that of the AP synthesis. After 2 h of reacting, all of the purified samples displayed similarly high crystallinity; however, the TFB-DB COF synthesized by the AP method had a significantly higher BET surface area (1015 m^2^ g^–1^) than those of the samples obtained in the control experiments (434–627 m^2^ g^–1^) (Figures 2c and S10a and Table S1).

Furthermore, the control synthesis conditions were applied to the synthesis of TFB-TAPB COF (TAPB = 1,3,5-tris(4-aminophenyl)benzene) to demonstrate the general applicability of the proposed theory. As expected, the TFB-TAPB COF with high crystallinity was successfully obtained with a yield of 43.8% by the AP synthesis method after a reaction time of 10 min (Figure S11 and Table S2). The syntheses under Controls 2 and 3 conditions gave much lower yields and lower crystallinity (Figure S12). Strikingly, protonation of the TAPB monomer resulted in a yield of 3% under Control 1 synthesis conditions, in this instance the decreased nucleophilicity of the amine has almost completely suppressed the condensation reaction, this is consistent with the results reported by Zomora et al.^31^ Even when the reaction time was extended to 2 h, the product yield from Control synthesis 1 remained low (22.4%), and the purified sample was predominantly amorphous with a low porosity and a surface area of 134 m^2^ g^–1^ (Figure 2f). The AP synthesis of TFB-TAPB COF had a significantly higher yield of 83.7% after 2 h and displayed significantly higher crystallinity and a higher surface area of 771 m^2^ g^–1^ (Figure 2e,f). These results demonstrate that preactivation of the aldehyde monomer by acid, while preventing amine protonation, is key to the efficient synthesis of imine-linked COF, in water at room temperature.

It is worth highlighting that the AP approach enables the rapid synthesis of crystalline and porous COF in water under ambient conditions, which is more challenging to achieve by conventional solvothermal methods. The crystallization of imine-linked COF is generally understood by the mechanism of dynamic imine exchange. The breaking and reforming of imine bonds, with appropriate reaction rates, allows for the self-correction of defects and crystallization.^46^ Since organic monomers are typically highly soluble in organic solvents, the polymerization is very fast under solvothermal conditions, and products isolated at the initial stages of the reactions are usually amorphous. This can be explained by the fact that the rate of imine formation was much faster than that of imine breaking in the initial stages of the reaction, and the resulting polymer was rich in defects and disordered structures. In contrast, the imine formation method described in this work, in an aqueous solution at room temperature, had a much slower rate, although that was significantly accelerated by the preactivation of the aldehyde. This is evidenced by the lower yield of the isolated product from aqueous solution, at the earlier stages of the reaction, than from the organic system (e.g., 95% for TAPB-TPA COF at 15 min).^43^ However, imine breaking is more favorable in aqueous solutions than in organic solvents. The decreased rate of imine formation and increased rate of imine braking in aqueous solutions allow for the modulation of the equilibrium such that the crystallization of the product occurs from the very beginning of the reaction, at room temperature (Figure S13).

**Figure 3:**
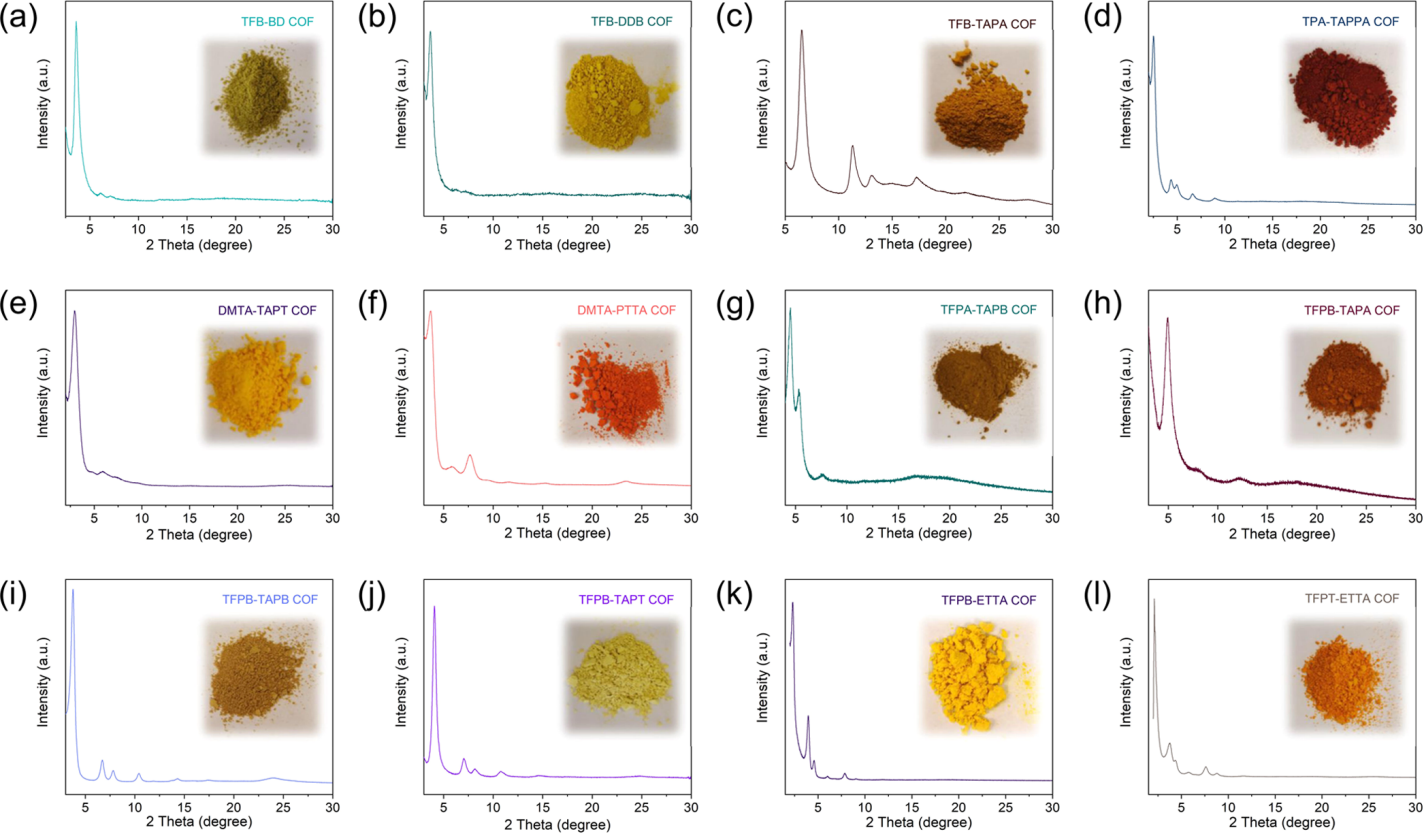
XRD patterns and optical images of TFB-BD COF (a), TFB-DDB COF (b), TFB-TAPA COF (c), TPA-TAPPA COF (d), DMTA-TAPT COF (e), DMTA-PTTA COF (f), TFPA-TAPB COF (g), TFPB-TAPA COF (h), TFPB-TAPB COF (i), TFPB-TAPT COF (j), TFPB-ETTA COF (k), and TFPT-ETTA COF (l).

To demonstrate the generality of the synthetic strategy proposed, we performed a range of reactions with different amine and aldehyde monomers in order to synthesize other imine-linked COFs. The following 14 additional COFs were synthesized with high crystallinity: TFB-BD COF,^33^ TFB-DDB COF,^47^ TFB-TAPA COF,^48^ TPA-TAPPA COF,^49^ DMTA-TAPT COF,^22^ DMTA-PTTA COF,^21^ TFPA-TAPB COF,^6^ TFPB-TAPA COF,^50^ TFPB-TAPB COF,^51^ TFPB-TAPT COF,^52^ TFPB-ETTA COF,^53^ TFPT-ETTA COF,^22^ TFB-ETTA COF,^21^ and TPA-TAPM COF^29^ (DMTA: 2,5-dimethoxybenzene-1,4-dicarboxaldehyde, TPA: terephthalaldehyde, TFPA: tris(4-formylphenyl)amine, TFPB: 1,3,5-tris(4-formylphenyl)-benzene, TFPT: 2,4,6-tris(4-formylphenyl)-1,3,5-triazine, BD: benzidine, DDB: 5,5′-diamino-2,2′-bipyridine, TAPA: tris(4-aminophenyl)-amine, TAPT: 4,4′,4″-(1,3,5-triazine-2,4,6-triyl)trianiline, PTTA: 4,4′,4″,4‴-(pyrene-1,3,6,8-tetrayl)tetraaniline, TAPPA: *N*,*N*,*N*′,*N*′-tetrakis(4-aminophenyl)-1,4-phenylenediamine, and TAPM: tetrakis(4-aminophenyl)methane. Their compositions and structures were characterized by scanning electron microscopy (SEM) (Figures S14, S19, and S34), infrared spectroscopy (Figures S15, S20, and S30), solid-state ^13^C nuclear magnetic resonance spectroscopy (Figure S16), XRD (Figures 3, S20 and S36), and N_2_ adsorption (Figures S17, S20, and S41). The XRD spectra of the COFs were in good agreement with similar data in the literature (Table S6). For example, the XRD spectra of TFPT-ETTA COF showed strong diffraction peaks at 2.1, 3.8, 4.4, and 7.6°, which can be assigned to (200), (001), (220), and (202) planes, respectively (Figure S18a).^22^ The transmission electron microscopy (TEM) images of the TFPB-TAPB COF clearly show the lattice fringes and the honeycomb-like porous structures, revealing the formation of a periodic porous structure and ordered framework alignment with a high degree of crystallinity (Figure S21). Since the solubility and reactivity of monomers are decreased in aqueous solutions with an increasing molecular size and degree of conjugation, the acidity of the reaction system was optimized to synthesize COF structures from large, conjugated organic monomers (Table S5). For example, the reaction of TFPT and ETTA in an aqueous solution with an acetic acid concentration of 4.38 M resulted in an amorphous and nonporous polymer product (surface area: 45 m^2^ g^–1^). By increasing the acid concentration was increased to 8.75 M, a crystalline and highly porous COF with a surface area of 965 m^2^ g^–1^ was obtained (Figure S18). All of the COFs had high surface areas of between 305 and 2487 m^2^ g^–1^, which were comparable to, or higher than, the same COFs synthesized by the conventional solvothermal method (Table S6). This strategy was found to be applicable to the COF synthesis with organic monomers of different sizes, geometries, and core structures. In addition to the most common [3 + 2] polymerization reactions that occur in COF synthesis, [3 + 3] and [4 + 2] connections were also found to be viable for the construction of COFs with different topological structures and dimensions (the numbers in the brackets refer to the amount of amine or aldehyde groups in the monomer). More importantly, the synthesis method worked with a variety of organic linkers that contained heteroatoms and pedant groups. For example, the use of TFPA, TAPPA, and TFPT monomers that contain tertiary amines or triazine functional groups formed the COFs with high crystallinity. Due to the presence of slightly twisted tertiary amine units in TFPA and TAPPA monomers, the COFs obtained with these monomers have contorted networks rather than the planar frameworks of COFs comprising fully aromatic monomers. Therefore, this new method demonstrates excellent generality for synthesizing various imine-linked COFs from aqueous solutions at room temperature. Finally, we performed scaled-up reactions and successfully synthesized TFB-DB COF and TPA-TAPPA COF on the gram scale (Figure S22). Scaling up the reaction did not demonstrate any significant influence on the properties of the product in terms of composition, crystallinity, or porosity (Figures S23, S24 and Tables S1, S3). It is hoped that this facile and green synthetic approach will be a powerful tool for exploring new COF structures.

**Figure 4:**
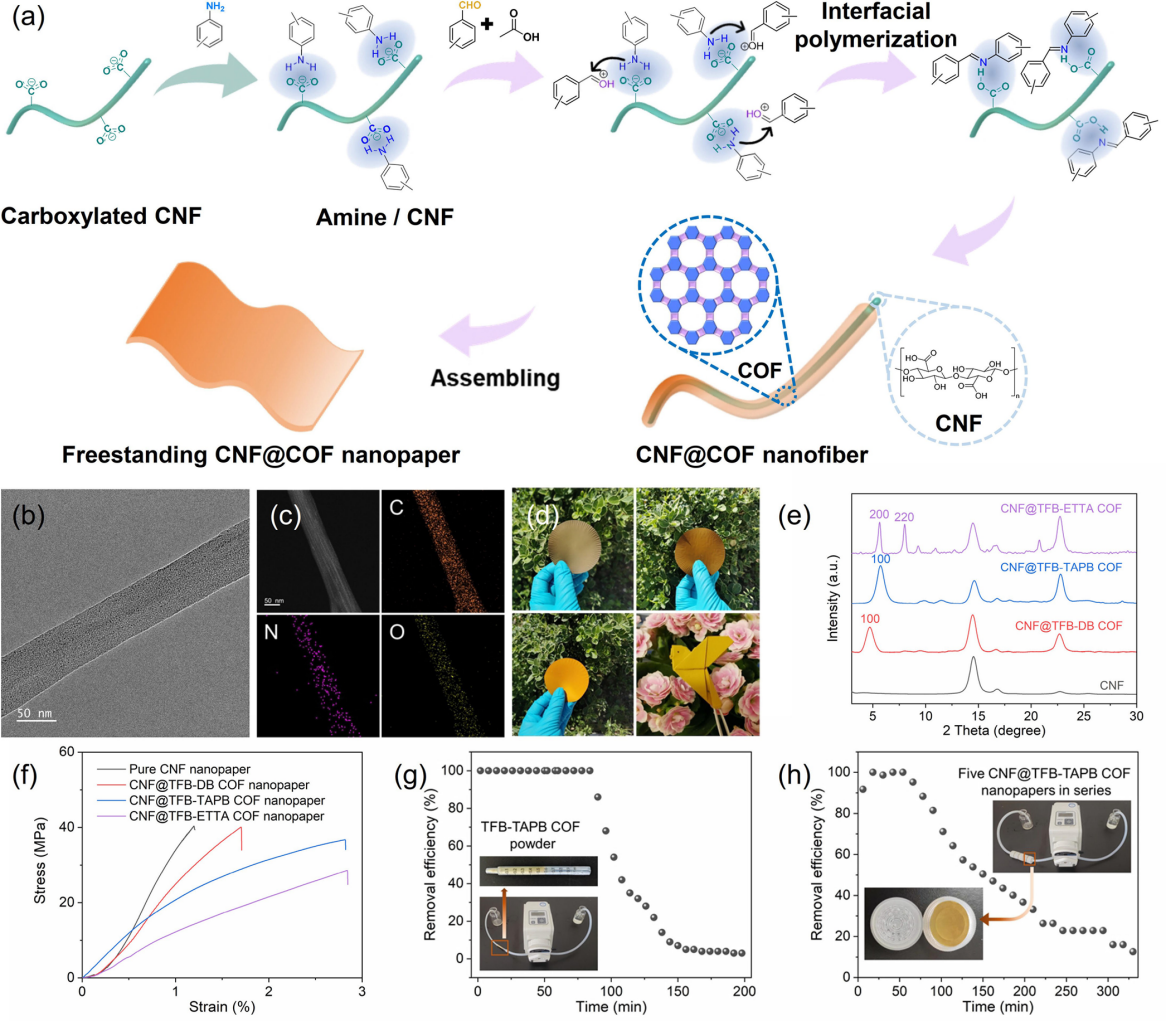
(a) Illustration of synthesis of CNF@COF nanofibers and freestanding nanopapers; transmission electron microscopy images (b) and energy-dispersive X-ray spectroscopy mapping (c) of CNF@TFB-TAPB COF nanofibers; (d) optical images of CNF@TFB-DB COF, CNF@TFB-TAPB COF, and CNF@ETTA COF nanopapers; (e) XRD patterns of pure CNF and CNF@COF nanopapers; (f) strain–stress curves of pure CNF and CNF@COF nanopapers; (g) removal efficiency of TFB-TAPB COF powder for ofloxacin from aqueous solution (10 ppm); and (h) removal efficiency of CNF@TFB-TAPB COF nanopapers for ofloxacin from aqueous solution (2 ppm).

Additionally, we have developed an interfacial synthesis approach for the growth of COF nanolayers on the surface of CNFs in aqueous solution, to address the challenges associated with processing COFs (Figure 4a). The unique attributes of CNFs, such as renewability, high surface area, abundant surface functionality, nanofibrous structure, good dispersibility, and water processability, position them as exceptional substrates or templates for the fabrication of various functional materials.^54−56^ In this study, carboxylated CNFs extracted from green algae were employed as substrates for engineering COFs (Figures S27–S29). Specifically, aldehyde and amine monomers were mixed with an aqueous acetic acid solution and an aqueous suspension of CNFs, respectively, followed by stirring for 1 h. Subsequently, the acid-activated aldehyde solution was introduced into the aqueous mixture of CNFs and the amine monomer under ambient conditions, and the resulting mixture was stirred for either 24 or 72 h. As a result, three distinct COFs were successfully synthesized with CNFs, yielding nanocomposites referred to as CNF@TFB-DB COF, CNF@TFB-TAPB COF, and CNF@TFB-ETTA COF. The purified CNF@COF products exhibited a characteristic nanofiber structure, with the nanofiber thickness ranging from 44 to 67 nm as observed from the TEM images (Figures 4b and S26). This thickness was significantly greater than that of pure CNFs (∼20 nm; Figure S27). Energy-dispersive X-ray spectroscopy (EDS) mapping of the nanofibers revealed the presence and uniform distribution of N, C, and O elements on the nanofiber surface (Figures 4c and S26c,i). Since pure CNFs lacked any N content, the observed N content on the hybrid nanofiber was attributed to the coated COF layer, which consisted of imine linkages and terminal amine groups. The IR spectra of the samples exhibited strong bands at 1616–1626 cm^–1^ corresponding to the vibration stretching of imine bonds (Figure S30). In contrast to the pure COF samples, the C=N vibration exhibited a blue shift of 5–10 cm^–1^ upon the growth of TFB-DB COF and TFB-TAPB COF on CNFs. This shift is likely attributed to the interaction between the imine groups within the COF and the carboxylate groups present on the surfaces of the CNFs in the CNF@COF samples. The presence of such an interaction is further supported by the study of X-ray photoelectron spectra (XPS). In addition to the main peak at 399.0 eV in the N 1s region for TFB-TAPB COF, a new shoulder peak emerged at 400.0 eV upon the growth of TFB-TAPB COF onto the surface of CNFs (Figure S32). SEM images of the samples at lower magnifications displayed homogeneous nanofibrous structures, without significant COF particles, indicating the high efficiency of the coating process and the templating effect of the CNFs in facilitating the growth of COF nanolayers (Figures S33 and S34). In contrast, the use of unmodified CNFs in the synthesis resulted in a significant amount of isolated COF nanoparticles in the composites, demonstrating the crucial role of CNF surface chemistry in the formation of the core–shell nanostructure of CNF@COF (Figure S35). Furthermore, XRD patterns of the CNF@COF samples exhibited characteristic diffraction peaks corresponding to COFs and CNFs, which closely matched the patterns of their individual counterparts (Figures 4e and S36). These results provide strong evidence for the successful deposition of COF layers onto the CNF surface.

Owing to the nanofibrous structure and good aqueous dispersibility of CNF@COFs (Figures S33 and S37), the suspension of CNF@COFs can be readily processed into freestanding, flexible, and foldable nanopapers through a series of vacuum filtration and drying steps (Figures 4d and S38). SEM images captured from the top views of the nanopapers illustrated the stacking and interlacing of the nanofibers, while the side view images revealed the layered structures (Figure S39). The COF content in the CNF@COF samples was determined to be in the range of 54–65 wt % through thermogravimetric analysis (Figure S40). The BET surface area of the CNF@COF nanopapers was calculated to range from 205 to 549 m^2^ g^–1^ based on N_2_ sorption isotherms (Figure S41 a–c). Pore size distribution analyses indicated the existence of hierarchical porous structures in CNF@COFs, with micropores originating from the COF components and mesopores attributed to the stacking of the hybrid nanofibers (Figure S41d–f). Remarkably, the CNF@COF nanopapers exhibited high tensile strength and high Young’s moduli of up to 40.2 MPa and 2.6 GPa, respectively, as evidenced by the strain–stress curves (Figure 4f). Compared to cellulose-COF composites prepared by the physical mixture method in previously reported studies (Table S7), the superior mechanical strength of the CNF@COF nanopapers can be attributed to the intertwined structure of the nanofibers and the high crystallinity of both CNF and COF components.

The high surface area and abundant microporosity inherent in COFs, as well as their incorporation into CNF@COF nanopapers, render them highly promising for wastewater treatment applications. Specifically, our study focused on the application of TFB-TAPB COF in the removal of trace amounts of ofloxacin (OFX), a widely used antibiotic, from aqueous solutions. Remarkably, TFB-TAPB COF exhibited relatively high adsorption capacities for OFX at low concentrations (e.g., 6.8 mg g^–1^ at *C*_e_ = 1.3 ppm, 11.8 mg g^–1^ at *C*_e_ = 4.1 ppm; where *C*_e_ denotes equilibrium concentration, Figure S44). Moreover, the adsorption kinetics demonstrated rapid uptake, with over 76.3% of the maximum adsorption capacity achieved within just 1 min at a 10 ppm concentration (Figure S45). For proof-of-concept applications, we utilized TFB-TAPB COF powder as a fixed adsorption bed to remove trace amounts of OFX from wastewater (Figure 4g). An efficient setup involved packing a plastic column with 180 mg of TFB-TAPB COF powder and passing a 10 ppm of OFX aqueous solution through the column at a constant flow rate of 0.17 mL min^–1^. Impressively, the COF column exhibited exceptional removal efficiency, capturing all OFX within the initial 85 min, and achieving an overall removal efficiency of 96.7% within 100 min. Furthermore, the freestanding nature and high mechanical strength of CNF@COF nanopapers inspired us to employ them as membranes in separation process. As depicted in the inset images of Figure 4h, a homemade filtration device was designed by connecting five filters in series, with each filter assembled with a piece of CNF@TFB-TAPB COF nanopaper with an effective area of 3.8 cm^2^ and a COF loading density of 3.6 mg cm^–2^. An aqueous OFX solution with a concentration of 2 ppm was passed through the apparatus at a constant flow rate of 0.17 mL min^–1^, and the OFX concentration in the outlet solution was monitored at intervals. Despite the lower amount of COF used (∼68.4 mg in five nanopapers) and the reduced OFX concentration compared to the column filtration experiment, the membrane filtration apparatus consistently exhibited an outstanding removal efficiency of nearly 100% during the initial 60 min. Importantly, the utilization of freestanding CNF@COF nanopapers as membranes for the capturing process provides significant advantages, including ease of operation, convenient recyclability, high flux rates, and high separation capacities when compared to traditional COF powder-based methods.

## Conclusions

A green, facile, general, and highly efficient strategy has been developed for synthesizing a variety of imine-linked COFs by stirring acid-preactivated aldehydes with amine monomers in aqueous solutions at room temperature. A range of control experiments were carried out, and the results clearly demonstrated that acid-preactivation of the aldehyde monomers, while avoiding amine protonation, significantly increased their reactivity in aqueous solutions and synergistically facilitated the formation of crystalline and porous COFs in high yields. Interestingly, the crystallization of COFs occurred at the very beginning of the reactions; this is proposed to be due to modulated rates of imine formation and breaking. This method further enables the uniform growth of COF nanolayers on the surface of CNFs through interfacial polymerization and the fabrication of freestanding CNF@COF nanopapers. Remarkably, the freestanding nanopapers exhibited high efficiency in removing a trace amount of OFX from aqueous solutions through a membrane separation process. We anticipate that this study will not only expedite the discovery of new COF materials but also establish a scalable, green synthesis and processing route for COFs, thereby advancing their practical use in real-world applications. The knowledge acquired from this study has the potential to drive advancements in the synthesis and processing of diverse functional porous materials by utilizing the principles of green and sustainable chemistry.

## Supporting Information Available

Experimental methods; additional results and discussions of PXRD patterns; NMR spectra; XPS spectra; IR spectra; N_2_ sorption isotherms; SEM images; and TEM images (PDF)
