# Supplementary material for: Ambient Aqueous Synthesis of Imine-Linked Covalent Organic Frameworks (COFs) and Fabrication of Freestanding Cellulose Nanofiber@COF Nanopapers
Source: J Am Chem Soc. 2023 Dec 19;146(1):742–51. doi: 10.1021/jacs.3c10691 (PMC10785817; doi:10.1021/jacs.3c10691)
Supplement: Supplementary file 1 — ja3c10691_si_001.pdf [file ja3c10691_si_001.pdf]

Supporting Information

Ambient Aqueous Synthesis of Imine-Linked Covalent Organic Frameworks (COFs) and Fabrication of Freestanding Cellulose Nanofiber @ COF Nanopapers

Xueying Kong[a], Zhongqi Wu[b], Maria Strømme[a], Chao Xu[ab]*

a Division of Nanotechnology and Functional Materials, Department of Materials Science and Engineering, Uppsala University, Uppsala, SE-75121, Sweden

b Institute of Molecular Engineering and Applied Chemistry, Anhui University of Technology, Ma'anshan, 243002 (P. R. China)

*Email: chao.xu@angstrom.uu.se

Table of Contents

1. Materials 3

2. Characterizations 3

3. Experimental Methods 4

4. Figures and Tables 12-52

5. References 53

1. Materials

1,3,5-triformylbenzene (TFB), 2,5-dimethoxybenzene-1,4-dicarboxaldehyde (DMTA), terephthalaldehyde (TPA), tris(4-formylphenyl)amine (TFPA), 1,3,5-tris(4-formLphenyl)-benzene (TFPB), 2,4,6-tris(4-formylphenyl)-1,3,5-triazine (TFPT), benzidine (BD), 5,5´-diamino-2,2´-bipyridine (DDB), tris(4-aminophenyl)-amine (TAPA), 1,3,5-tris(4-aminophenyl)benzene (TAPB), 4,4',4''-(1,3,5-triazine-2,4,6-triyl)trianiline (TAPT), 4,4',4'',4'''-(pyrene-1,3,6,8-tetrayl)tetraaniline (PTTA), 4,4′,4″,4‴-(ethene-1,1,2,2-tetryl)tetraaniline (ETTA), N, N, N´, N´-tetrakis(4-aminophenyl)-1,4-phenylenediamine (TAPPA) and tetrakis(4-aminophenyl)methane (TAPM) were purchased from Tensus Biotech. 1,4-diaminebenzene (DB) and acetic acid were purchased from Alfa Aesar. All reagents and solvents were used without extra purification. Cellulose nanofibers (CNFs) were provided by FMC Biopolymer, originally extracted from *Cladophora* algae. Carboxylated CNFs were obtained through a TEMPO oxidation method, following a previously reported procedure.[1]

2. Characterizations

The morphology of covalent organic frameworks (COF) and CNF@COF nanocomposites were investigated by using a scanning electron microscope (SEM, Zeiss, Leo Gemini 1530) and a transmission electron microscope (TEM, JEM-F200). The energy-dispersive X-ray spectroscopy of CNF@COFs were investigated by using the TEM instrument. N_2 sorption isotherms were recorded on a Micromeritics ASAP 2020 unit at 77 K. The DFT model was used to analyse the pore size distributions based on the adsorption isotherms. Before measurement, the samples were degassed at 100 °C for 6 h under a kinetic vacuum ($<10^{-5}$ mmHg). Powder X-ray diffraction (XRD) patterns were recorded on a Bruker Focus D8 diffractometer (Cu Kα radiation, λ = 1.5418 Å). The infrared (IR) spectra were recorded on a Bruker Tensor 27 spectrometer. The thermogravimetric curves were measured on a thermogravimetric analyzer (Mettler Toledo, TGA/SDTA851e) under a N_2 flow. The mechanical properties of both CNF and CNF@COF nanopapers were assessed at room temperature using an Instron Instrument (Model 5944) operating at a cross-head speed of 3 mm/min. This evaluation was conducted in accordance with the standard test method outlined in ASTM D-638. UV-vis spectra were recorded on a Shimadzu UV-2600 UV-VIS spectrophotometer. Solid-state ^{13}C nuclear magnetic resonance (NMR) spectra were recorded on a Bruker AVWBIII600 spectrometer. X-ray photoelectron spectra (XPS) were recorded on a Physical Electronics Quantera II Scanning XPS Microprobe.

3. Experimental Methods

3.1 Synthesis of COFs powder in water

3.1.1 Synthesis of TFB-DB COFs in aqueous solutions with different Methods

Aldehyde preactivation (AP) method:

104 mg (0.64 mmol) TFB and 4 mL aqueous acetic acid solution (8.75 M) were added into a 20 mL glass vial. The mixture was stirred at room temperature for 30 min and a homogeneous suspension was formed. Subsequently, 104 mg (0.96 mmol) DB was dissolved in 4 mL deionized (DI) water and the solution was dropwise added into the glass vial. The mixture was stirred at room temperature for varying durations (1 min – 168 h).

Control synthesis 1:

104 mg (0.64 mmol) TFB and 4 mL DI water were added into a 20 mL glass vial and the mixture was stirred at room temperature for 30 min. Meanwhile, 104 mg (0.96 mmol) DB was dissolved in 4 mL aqueous acetic acid solution (8.75 M) and the solution was dropwise added into the glass vial. The mixture was stirred at room temperature for varying durations (10 min or 2 h).

Control synthesis 2:

104 mg (0.64 mmol) TFB and 4 mL acetic acid (4.38 M) were added into a 20 mL glass and the mixture was stirred at room temperature for 30 min. Meanwhile, 104 mg (0.96 mmol) DB was dissolved in 4 mL acetic acid (4.38 M) assisted by sonication and the solution was dropwise added into the glass vial. The mixture was stirred at room temperature for varying durations (10 min or 2 h).

Control synthesis 3:

104 mg (0.64 mmol) TFB and 3 mL DI water were added into a 20 mL glass vial and the mixture was stirred at room temperature for 30 min. Meanwhile, 104 mg (0.96 mmol) DB was dissolved in 3 mL DI water assisted by sonication and the solution was dropwise added into the glass vial. Subsequently, 2 mL acetic acid was dropwise added into the mixture solutions and the mixture solution was stirred at room temperature for varying durations (10 min or 2h).

Gram-scale synthesis:

1.04 g (6.4 mmol) TFB and 40 mL acetic acid (8.75 M) were added into a 500 mL glass bottle and the mixture was stirred at room temperature for 30 min. Meanwhile, 1.04 g (9.6 mmol) DB was dissolved 40 mL DI water assisted by sonication and the solution was dropwise added into the glass bottle. The mixture solutions were stirred at room temperature for 2 h.

All of the collected precipitates were purified by Soxhlet extraction using acetone and then dried in an oven at 70 °C for 12 h. The yield of TFB-DB COFs was given in Table S1.

Table S1. Synthesis conditions and results of TFB-DB COF

Entry	Synthesis method	Reaction time	Temperature (°C)	Concentration of acetic acid (M) [a]	Solvent	Yield (%)	S_{BET} ($m^2\ g^{-1}$)
1	AP method	1 min	RT[b]	4.38	Water	23.1	893
2	AP method	5 min	RT	4.38	Water	33.9	1074
3	AP method	10 min	RT	4.38	Water	47.7	1205
4	Control synthesis 1	10 min	RT	4.38	Water	8.1	-
5	Control synthesis 2	10 min	RT	4.38	Water	20.2	-
6	Control synthesis 3	10 min	RT	4.38	Water	18.5	-
7	AP method	30 min	RT	4.38	Water	50.3	1011
9	AP method	1 h	RT	4.38	Water	69.5	1028
10	AP method	2 h	RT	4.38	Water	87.8	1015
11	Control synthesis 1	2 h	RT	4.38	Water	48.9	434
12	Control synthesis 2	2 h	RT	4.38	Water	65.9	592
13	Control synthesis 3	2 h	RT	4.38	Water	66.8	627
15[c]	AP method	2h	RT	4.38	Water	79.2	1081
16	AP method	4 h	RT	4.38	Water	79.6	1067
17	AP method	24 h	RT	4.38	Water	80.6	982
18	AP method	72 h	RT	4.38	Water	75.5	944
19	AP method	168 h	RT	4.38	Water	77.3	859

[a]: Concentration of acetic acid in the final solution.

[b]: RT ≈ 22 °C.

[c]: Gram-scale synthesis

3.1.2 Synthesis of TFB-TAPB COF

Different batches of TFB-TAPB COF were synthesized, which followed the method as above described that used for synthesizing TFB-DB COF. 104 mg (0.64 mmol) TFB and 225 mg (0.64 mmol) TAPB were used in each batch of synthesis. The synthesis conditions and results were given in Table S2.

Table S2. Synthesis conditions and results of TFB-TAPB COF

Entry	Synthesis method	Reaction time	Temperature (°C)	Concentration of acetic acid (M) [a]	Solvent	Yield (%)	S_{BET} (m^2 g^{-1})
1	AP method	10 min	RT[b]	4.38	Water	43.8	292
2	Control synthesis 1	10 min	RT	4.38	Water	3	-
3	Control synthesis 2	10 min	RT	4.38	Water	18.3	-
4	Control synthesis 3	10 min	RT	4.38	Water	19.9	-
5	AP method	2 h	RT	4.38	Water	83.7	771
6	Control synthesis 1	2 h	RT	4.38	Water	22.4	134
7	Control synthesis 2	2 h	RT	4.38	Water	65.1	427
8	Control synthesis 3	2 h	RT	4.38	Water	25.1	282
9	AP method	72 h	RT	4.38	Water	80.0	1415

[a]: Concentration of acetic acid in the final solution.

[b]: RT ≈ 22 °C.

3.1.3 Synthesis of TFB-ETTA COF

104 mg (0.64 mmol) TFB and 4 mL 8.75 M acetic acid were added into a 20 mL glass vial. The mixture was stirred at room temperature for 30 min and a homogeneous suspension was formed. Subsequently, 188 mg (0.48 mmol) ETTA was dissolved in 4 mL DI water and the solution was dropwise added into the glass vial. The mixture was stirred at room temperature for 72 h. All of the collected precipitates were purified by Soxhlet extraction using acetone and then dried in an oven at 70 °C for 12 h. The yield of TFB-ETTA COFs is 81%.

3.1.4 Synthesis of TFB-BD COF

104 mg (0.64 mmol) TFB and 4 mL 8.75 M acetic acid were added into a 20 mL glass vial. The mixture was stirred at room temperature for 30 min and a homogeneous suspension was formed. Subsequently, 177 mg (0.96 mmol) BD was dissolved in 4 mL DI water, and the solution was dropwise added into the glass vial. The mixture was stirred at room temperature for 72 h. All of the collected precipitates were purified by Soxhlet extraction using acetone and then dried in an oven at 70 °C for 12 h. The yield of collected TFB-DB COF is 87 %.

3.1.5 Synthesis of TFB-DDB COF

TFB-DDB COF was synthesized via AP method, which followed the method as above described that were used for synthesizing TFB-BD COF. 104 mg (0.64 mmol) TFB with 4 mL 17.5 M acetic acid and 178 mg (0.96 mmol) DDB with 4 mL DI water were used in the synthesis. The yield of collected TFB-DDB COF is 58 %.

3.1.6 Synthesis of TFB-TAPA COF

TFB-TAPA COF was synthesized via AP method, which followed the method as above described that were used for synthesizing TFB-BD COF. 52 mg (0.32 mmol) TFB with 2 mL 8.75 M acetic acid and 93 mg (0.32 mmol) TAPA with 2 mL DI water were used in the synthesis. The yield of collected TFB-TAPA COF is 90 %.

3.1.7 Synthesis of TPA-TAPPA COF

TPA-TAPPA COF was synthesized via AP method, which followed the method as above described that were used for synthesizing TFB-BD COF. 80 mg (0.6 mmol) TPA with 4 mL 17.5 M acetic acid and 142 mg (0.3 mmol) TAPPA with 4 mL H_2O were used in the synthesis. The yield of collected TPA-TAPPA COF is 72 %.

Gram-scale synthesis:

TPA-TAPPA COF was synthesized via AP method, which followed the method as above described that were used for synthesizing TFB-BD COF. 800 mg (6 mmol) TPA with 40 mL 17.5 M acetic acid and 1420 mg (3 mmol) TAPPA with 40 mL 8 M acetic acid were used in the synthesis. The yield of collected TPA-TAPPA COF is 74 %.

Table S3. Synthesis conditions and results of TPA-TAPPA COF.

Entry	Synthesis method	Reaction time	Reaction temperature	Concentration of acetic acid (M) [a]	Solvent	Yield (%)	S_{BET} ($m^2 g^{-1}$)
1	AP method	72 h	RT[b]	13.2	Water	72	923
2[c]	AP method	72 h	RT[b]	13.2	Water	74	1003

[a] Concentration of acetic acid in the final solution.

[b] RT ≈ 22 °C.

[c] Gram-scale synthesis

3.1.8 Synthesis of DMTA-TAPT COF

DMTA-TAPT COF was synthesized via AP method, which followed the method as above described that were used for synthesizing TFB-BD COF. 58 mg (0.3 mmol) DMTA with 2 mL 8.75 M acetic acid and 71 mg (0.2 mmol) TAPT with 2 mL DI water were used in the synthesis. The yield of collected DMTA-TAPT COF is 73 %.

3.1.9 Synthesis of DMTA-PTTA COF

DMTA- PTTA COF was synthesized via AP method, which followed the method as above described that were used for synthesizing TFB-BD COF. 58 mg (0.3 mmol) DMTA with 2 mL 8.75 M acetic acid and 84.9 mg (0.15 mmol) PTTA with 2 mL DI water were used in the synthesis. The yield of collected DMTA- PTTA COF is 83 %.

3.1.10 Synthesis of TFPA-TAPB COF

TFPA-TAPB COF was synthesized via AP method, which followed the method as above described that were used for synthesizing TFB-BD COF. 92.9 mg (0.32 mmol) TFPA with 2 mL 17.5 M acetic acid and 124.8 mg (0.32 mmol) TAPB with 2 mL DI water were used in the synthesis. The yield of collected TFPA-TAPB COF is 74 %.

3.1.11 Synthesis of TFPB-TAPA COF

TFPB-TAPA COF was synthesized via AP method, which followed the method as above described that were used for synthesizing TFB-BD COF. 124.8 mg (0.32 mmol) TFPB with 2 mL 8.75 M acetic acid and 93 mg (0.32 mmol) TAPA with 2 mL DI water were used in the synthesis. The yield of collected TFPB-TAPA is 73 %.

3.1.12 Synthesis of TFPB-TAPB COF

TFPB-TAPB COF was synthesized via AP method, which followed the method as above described that were used for synthesizing TFB-BD COF. 124.8 mg (0.32 mmol) TFPB with 2 mL 17.5 M acetic acid and 112.5 mg (0.32 mmol) TAPB with 2 mL DI water were used in the synthesis. The yield of collected TFPB-TAPB COF is 82 %.

3.1.13 Synthesis of TFPB-TAPT COF

TFPB-TAPT COF was synthesized via AP method, which followed the method as above described that were used for synthesizing TFB-BD COF. 124.8 mg (0.32 mmol) TFPB with 2 mL 8.75 M acetic acid and 94.4 mg (0.24 mmol) TAPT with 2 mL DI water were used in the synthesis. The yield of collected TFPB-TAPT COF is 92 %.

3.1.14 Synthesis of TFPB-ETTA COF

TFPB-ETTA COF was synthesized via AP method, which followed the method as above described that were used for synthesizing TFB-BD COF. 124.8 mg (0.32 mmol) TFPB with 2 mL 17.5 M acetic acid and 94.2 mg (0.24 mmol) ETTA with 2 mL DI water were used in the synthesis. The yield of collected TFPB-ETTA COF is 65 %.

3.1.15 Synthesis of TFPT-ETTA COF

TFPT-ETTA COF was synthesized via AP method, which followed the method as above described that were used for synthesizing TFB-BD COF. 125.9 mg (0.32 mmol) TFPT with 2 mL 17.5 M acetic acid and 94.2 mg (0.24 mmol) ETTA with 2 mL DI water were used in the synthesis. The yield of collected TFPT-ETTA COF is 81 %.

3.1.16 Synthesis of TPA-TAPM COF (COF-300)

TPA-TAPM COF was synthesized via AP method, which followed the method as above described that were used for synthesizing TFB-BD COF. 64.5 mg (0.48 mmol) TPA with 4 mL 17.5 M acetic acid and 91.2 mg (0.24 mmol) TAPM with 4 mL H_2O were used in the synthesis. The yield of collected TPA-TAPM COF is 88 %.

3.2 Synthesis of CNF@COF nanofibers and fabrication of nanopapers

3.2.1 Synthesis of CNF@TFB-DB COF and fabrication of nanopaper

To synthesize CNF@TFB-DB COF, 52 mg of DB was dissolved in an aqueous suspension containing carboxylated CNFs (10 mL, 6.5 mg/mL). The mixture was stirred at 1000 rpm for 60 min. Separately, 52 mg of TFB and 10 mL of aqueous acetic acid solution (8.75 M) were combined in a 20 mL glass vial and stirred at room temperature for 60 min to form a homogeneous suspension. Next, the TFB solution was added dropwise into the DB/CNF solution, and the resulting mixture was stirred at room temperature for 24 h. The collected precipitates were purified using acetone in a Soxhlet extractor. The purified CNF@TFB-DB COF was washed with water and then dispersed in water using a probe sonicator. The resulting homogeneous suspension was filtered through a Durapore® PVDF membrane filter (pore size: 0.45 μm, diameter: 9 cm). Finally, a freestanding CNF@TFB-DB COF nanopaper was obtained after drying in an oven at 70 °C for 12 h. The CNF@TFB-DB COF nanopaper contained 51% TFB-DB COFs, as calculated by thermogravimetric curves.

3.2.2 Synthesis of CNF@TFB-TAPB COF and fabrication of nanopaper

CNF@TFB-TAPB COF was synthesized using the same procedure described above for CNF@TFB-DB COF. Specifically, 70.5 mg of TAPB and 32 mg of TFB were used in the synthesis, and the reaction time was extended to 72 h. The fabrication of CNF@TFB-TAPB COF nanopaper followed the same method as used for CNF@TFB-DB COF nanopaper. The CNF@TFB-TAPB COF nanopaper contained 64% TFB-TAPB COFs, as calculated by thermogravimetric curves.

3.2.3 Synthesis of CNF@TFB-ETTA COF and fabrication of nanopaper

The synthesis of CNF@TFB-ETTA COF followed the procedure used for CNF@TFB-DB COF. Specifically, 59 mg of ETTA and 32 mg of TFB were used in the synthesis, and the reaction time was extended to 72 hours. The fabrication of CNF@TFB-ETTA COF nanopaper followed the same method as used for CNF@TFB-DB COF nanopaper. The CNF@TFB-ETTA COF nanopaper contained 55% TFB-ETTA COFs, as calculated by thermogravimetric curves.

3.2.4 Synthesis of COFs with unmodified CNFs

The synthesis followed the procedure used for CNF@COF, wherein an aqueous solution containing unmodified CNF (3 mg mL^{-1}, 22 mL) was employed to replace the carboxylated CNF.

3.3 Adsorption experiment

3.3.1 Adsorption kineties

A total of 25 mg of TFB-TAPB COF powder was added to 50 mL ofloxacin (OFX) solution (10 ppm) and the mixture was stirred at room temperature. 2 mL of aliquots were withdrawn from the suspension at intervals of 1, 3, 15, 30, 45, 90, 120, and 240 min and the solid from the suspensions was removed by centrifugation. The concentration of ofloxacin in the supernatants were analysed by UV-vis spectroscopy and the calibration curve.

3.3.2 Adsorption isotherm

5 mg of TFB-TAPB COF powder was added to aqueous OFX solutions with different concentrations (2−20 ppm) containing a same amount of OFX (0.1 mg). The mixture were stirred at room temperature for 12 h to reach equilibrium. Subsequently, TFB-TAPB COF powder was removed from the mixtures by centrifugation. The concentration of ofloxacin in the resulting supernatants was analysed by UV-vis spectroscopy with reference to the calibration curve. The adsorption capacity (q_e) was calculated using the following equation:

$$q_e = \frac{(C_0 - C_e) \times V}{m}$$

Where q_e (mg g^{-1}) is the equilibrium adsorption capacity, C_0 (ppm) is the initial concentration of OFX, and C_e (ppm) is the residual concentration of OFX after the adsorption.

The calculated adsorption capacities at different concentrations were plotted and fitted by Langmuir model:

$$\frac{C_e}{q_e} = \frac{C_e}{q_{max,e}} + \frac{1}{q_{max,e}K_L}$$

Where $q_{max,e}$ (mg g^{-1}) denotes the maximum adsorption capacity, K_L (mg $^{-1}$) is the Langmuir constant.

3.3.3 Trace ofloxacin removal from aqueous solution using TFB-TAPB COF powder and CNF@TFB-TAPB COF nanopaper

TFB-TAPB COF powder:

180 mg TFB-TAPB COF powder were packed into a plastic column. An aqueous solution of OFX with a concentration of 10 ppm was passed through the column at a constant flow rate of

0.17 mL min^{-1}. The concentration of OFX in the outlet solution was monitored at different intervals.

CNF@TFB-TAPB COF nanopaper:

A homemade filtration device was designed by connecting five filters in series and each filter was assembled with a piece of CNF@TFB-TAPB COF nanopaper with an effective area of 3.8 cm^2 and a COF loading density of 3.6 mg cm^{-2}. An aqueous OFX solution with a concentration of 2 ppm was passed thought the apparatus at a constant flow rate of 0.17 mL min^{-1} and the concentration of OFX in the outlet solution was monitored at different intervals.

The removal efficiency was calculated using the formula:

$$Removel\ efficiency = \frac{C_0 - C_t}{C_0} \times 100\ \%$$

Where C_0 (ppm) is the initial concentration of OFX, and C_t (ppm) is the residual concentration of ofloxacin after the separation process.

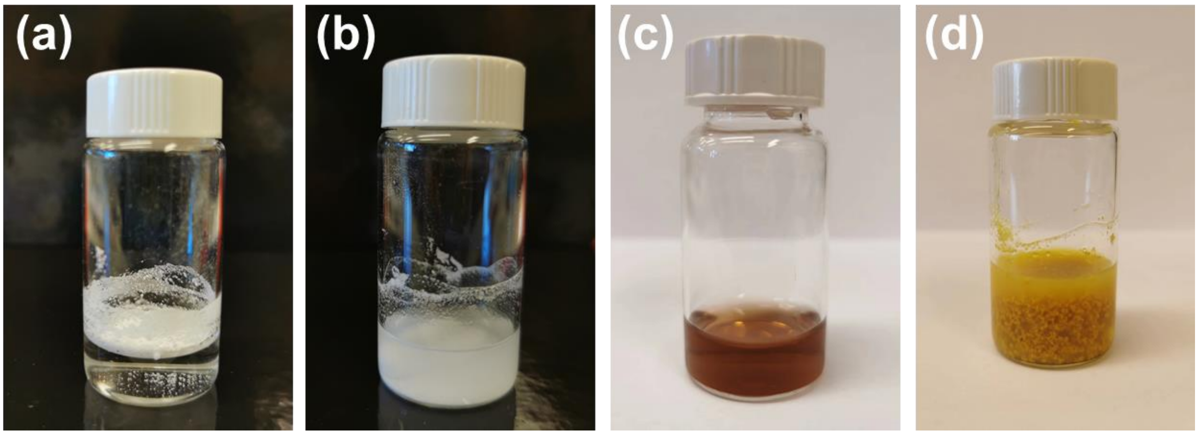

Figure S1. (a) A mixture consisting of 104 mg TFB and 4 mL water; (b) A mixture consisting of 104 mg TFB and 4 mL aqueous acetic acid solution (8.75 M); (c) A solution of 104 mg DB in 4 mL water; (d) Formation of yellow precipitate once the DB solution (c) was added into the TFB suspension (b).

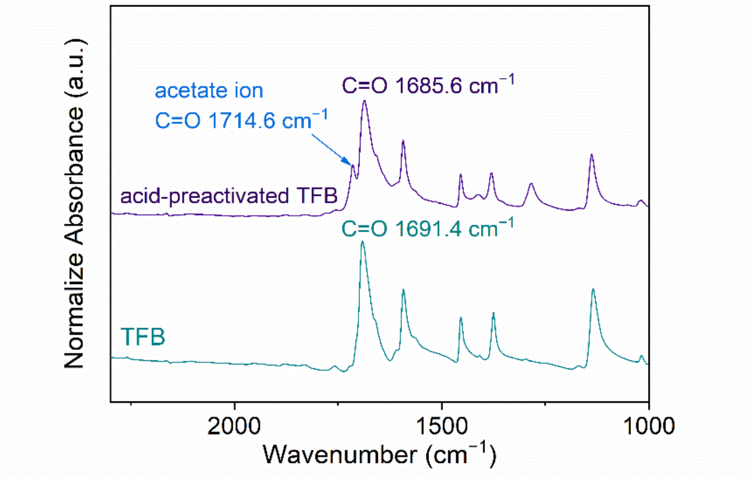

Figure S2. Comparison of infrared spectra of TFB and acid-preactivated TFB.

The protonation of the aldehyde group increasing the hydrophilicity and aqueous solubility (Figure S1b) of the monomer, as evidenced by the infrared spectra of TFB and its acid-treated sample (Figure S2). The strong stretching vibration of C=O bond of TFB at 1691 cm^{-1} slightly shifted to 1686 cm^{-1} after the acetic acid treatment. In addition, a new peak at 1715 cm^{-1} was observed, which can be attributed to the C=O stretching vibration of the acetate ion.

Additional discussion on the synthesis and characterization of TFB-DB COF

Ten parallel syntheses of TFB-DB COF were performed and the precipitates were analyzed at the same time points as before, however, in this instance the precipitates were thoroughly washed with water, acetone and then further purified by Soxhlet extraction using acetone. The samples obtained displayed similarly nanofibrous morphology (Figure S3) and high crystallinity as revealed by SEM and XRD analyses, respectively. The diffraction peaks observed at $2\theta \approx 4.8°$, 8.4°, 9.6°, 12.6°, and 25.7° in the XRD patterns were attributed to the (100), (110), (200), (210), and (001) planes of TFB-DB COF (Figure S4a), respectively.[2] The analysis of the full width at half maxima (FWHM(100)) from the XRD patterns revealed that the sample collected after 10 min of reaction appeared to have the highest crystallinity (Figure S5), which is consistent with the result of time-dependent BET surface area.[3] Infrared spectra of the COF samples displayed strong stretching vibrations at 1616 cm^{-1}, indicating the formation of imine bonds. In contrast to the C=O vibration at 1691 cm^{-1} observed in TFB, the intensity of the vibration signals (1696 cm^{-1}) of the aldehyde functional groups were significantly reduced in the COF samples, indicating the consumption of aldehyde during the condensation reaction (Figure 1c and S4b). In addition, the relative intensity ratio of the IR

peaks at 1616 cm^{-1} and 1696 cm^{-1} had no significant changes after 2 h of reaction, which suggested that the reaction reached an equilibrium at 2 h.

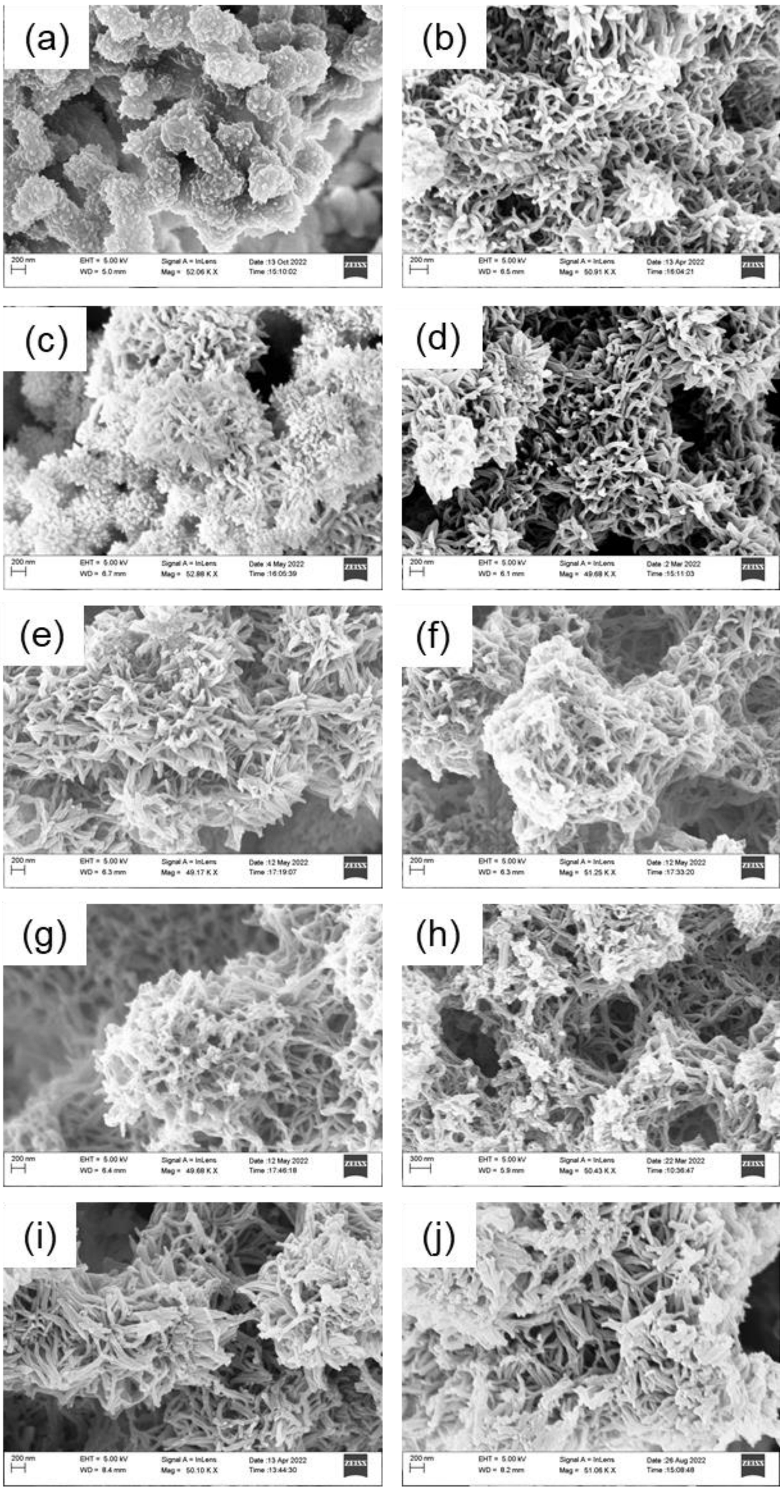

Figure S3. SEM images of the TFB-DB COFs that were synthesized using the AP method at room temperature with different reaction intervals (1 min (a), 5 min (b), 10 min (c), 30 min (d), 1 h (e), 2 h (f), 4 h (g), 24 h (h), 72 h (i), and 168 h (j)).

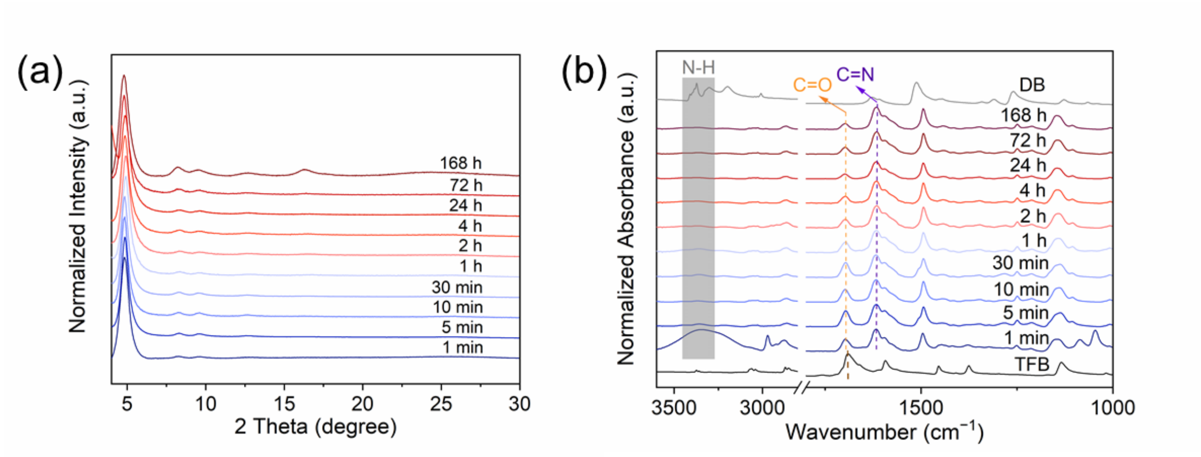

Figure S4. (a) XRD patterns and (b) IR spectra of the purified TFB-DB COFs that were synthesized using the AP method at room temperature with different reaction intervals (1, 5, 30 min, 1, 2, 4, 24, 72, and 168 h). The samples obtained displayed similarly high crystallinity as revealed by XRD analyses. The diffraction peaks observed at 2θ ≈ 4.8°, 8.4°, 9.6°, 12.6°, and 25.7° in the XRD patterns were attributed to the (100), (110), (200), (210), and (001) planes of TFB-DB COF, respectively.[2]

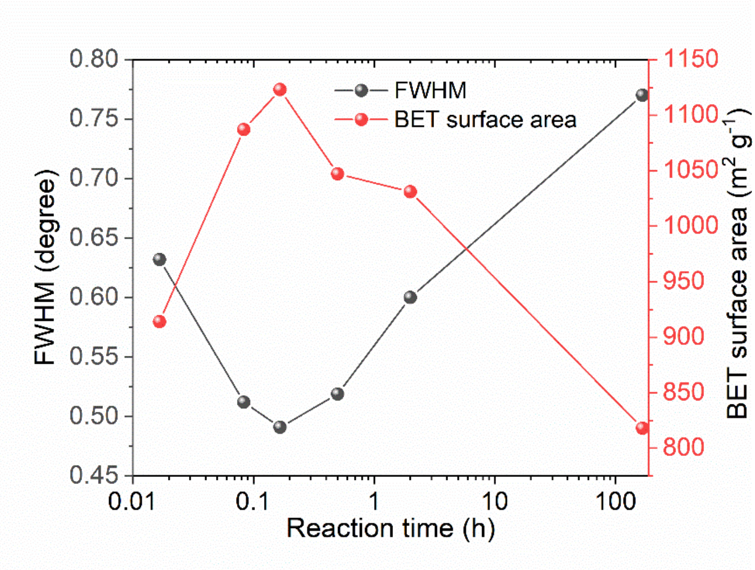

Figure S5. Comparison of the FWHM $_{(100)}$ and BET surface area among the obtained TFB-DB COFs with different reaction time.

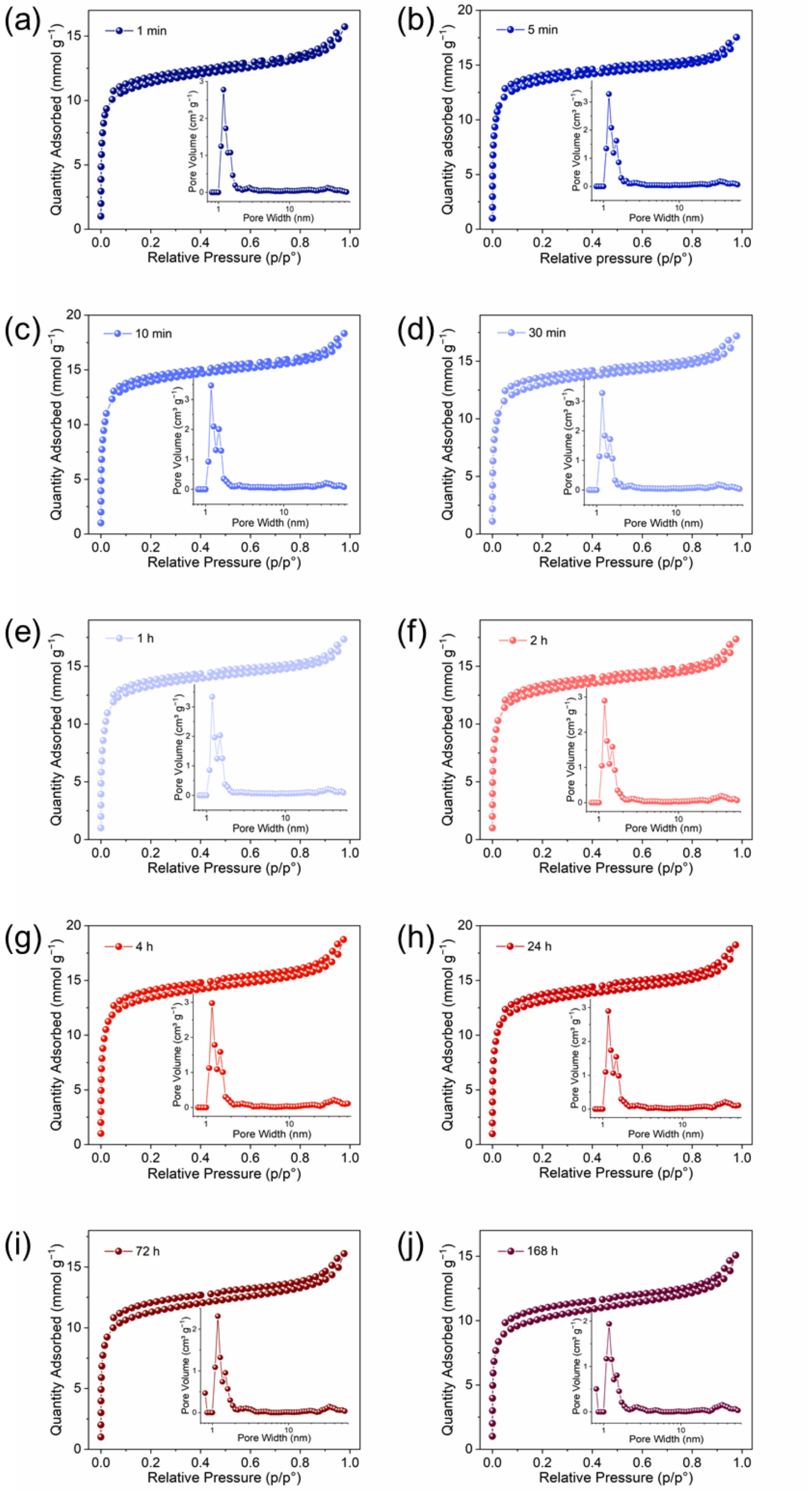

Figure S6. N_2 adsorption-desorption isotherms of the purified TFB-DB COFs that were synthesized using the AP method at room temperature with different reaction intervals (1, 5,

30 min, 1, 2, 4, 24, 72, and 168 h). The isotherms were recorded at 77 K. The insert figures showed the pore size distributions of the samples that were obtained by analysing the adsorption branches using a density functional theory (DFT) model.

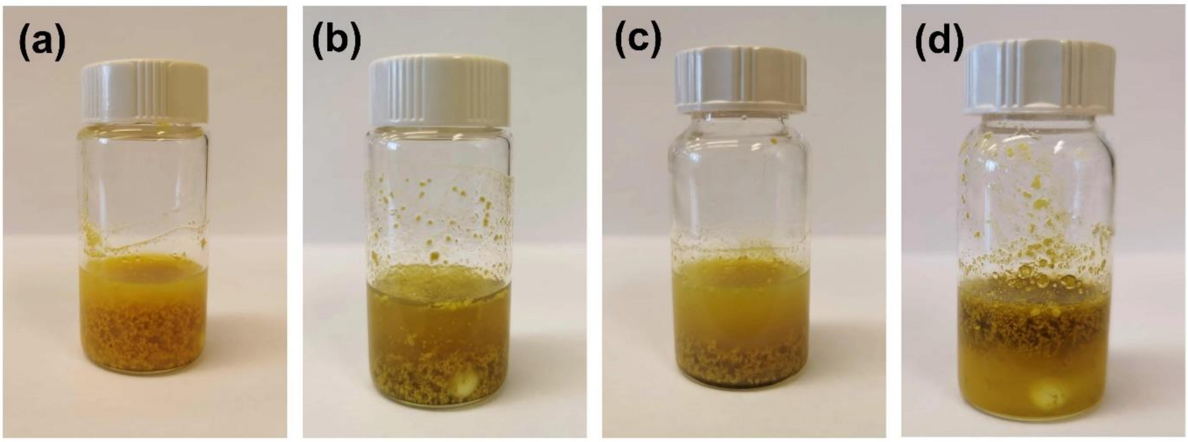

Figure S7. Optical images showing the initial stage of the reactions for synthesizing TFB-DB COF using different methods at room temperature. (a) AP synthesis method, (b) control synthesis 1, (c) control synthesis 2, and (d) control synthesis 3.

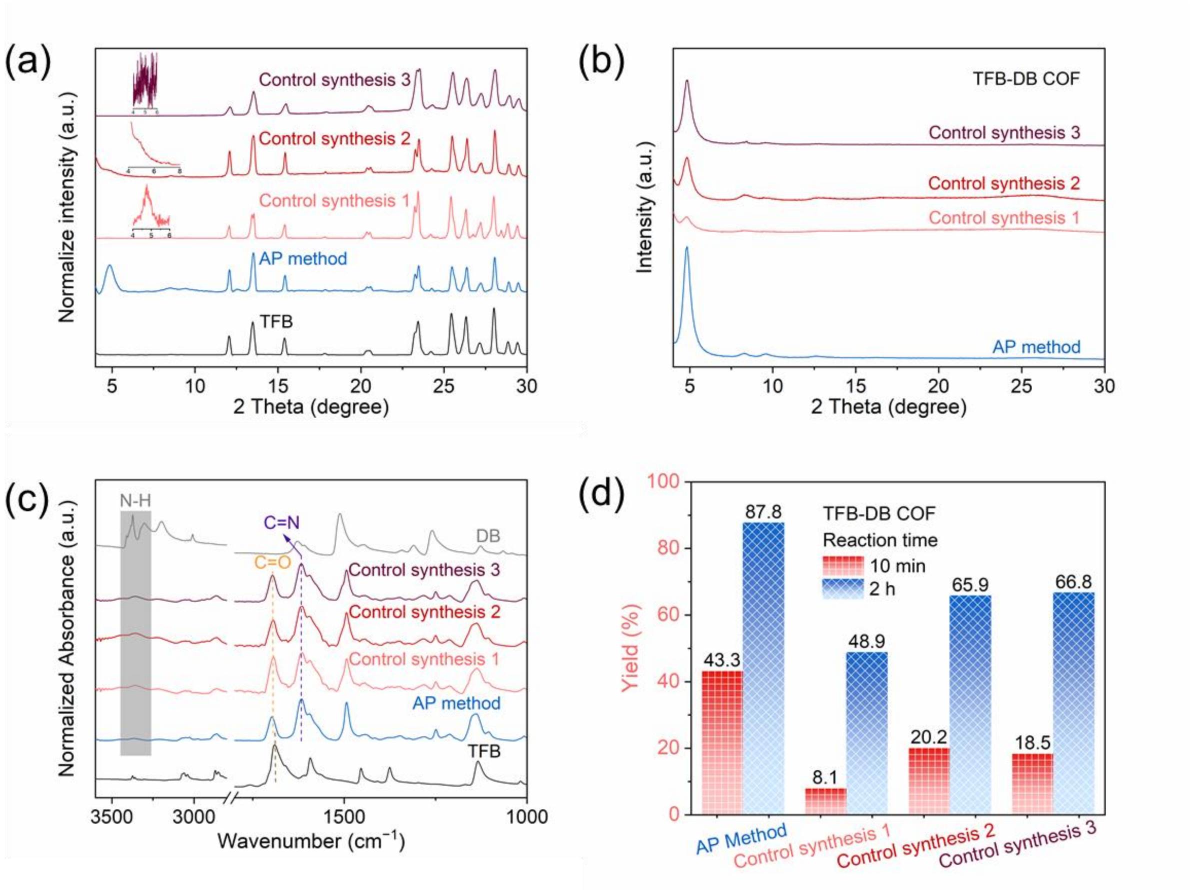

Figure S8. (a) Comparison of XRD patterns of the TFB monomer and the crude TFB-DB COF samples synthesized by different methods at room temperature with a reaction time of 10 min. The samples were washed with water to remove acetic acid and unreacted DB monomer prior to the measurements. (b) XRD patterns of the purified TFB-DB COF samples synthesized by different methods at room temperature with a reaction time of 10 min. The samples were thoroughly washed by water and acetone to remove acetic acid and any unreactive monomers prior to the measurements; (c) Comparison of IR spectra of the TFB and DB monomers with the purified TFB-DB COF samples synthesized by different methods at room temperature with a reaction time of 10 min (d) Comparison of the yield of TFB-DB COFs synthesized by different methods at room temperature with a reaction time of 10 min and 2 h.

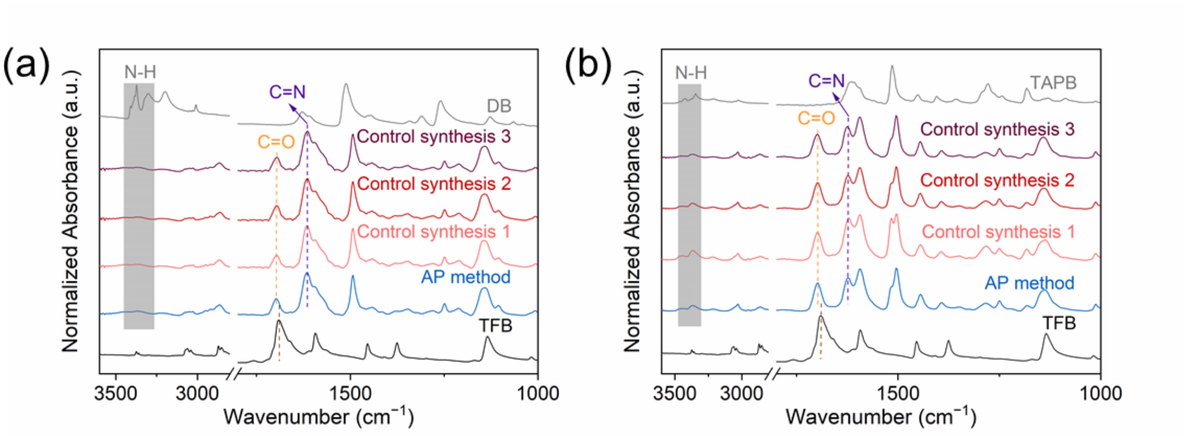

Figure S9. (a) Comparison of IR spectra of the TFB and DB monomers with the purified TFB-DB COF samples synthesized by different methods at room temperature with a reaction time of 2 h. The samples were washed with water and acetone to remove acetic acid and unreacted DB monomer prior to the measurements. (b) Comparison of IR spectra of the TFB and TAPB monomers with the purified TFB-TAPB COF samples synthesized by different methods at room temperature with a reaction time of 2 h. The samples were washed with water and acetone to remove acetic acid and unreacted TAPB monomer prior to the measurements.

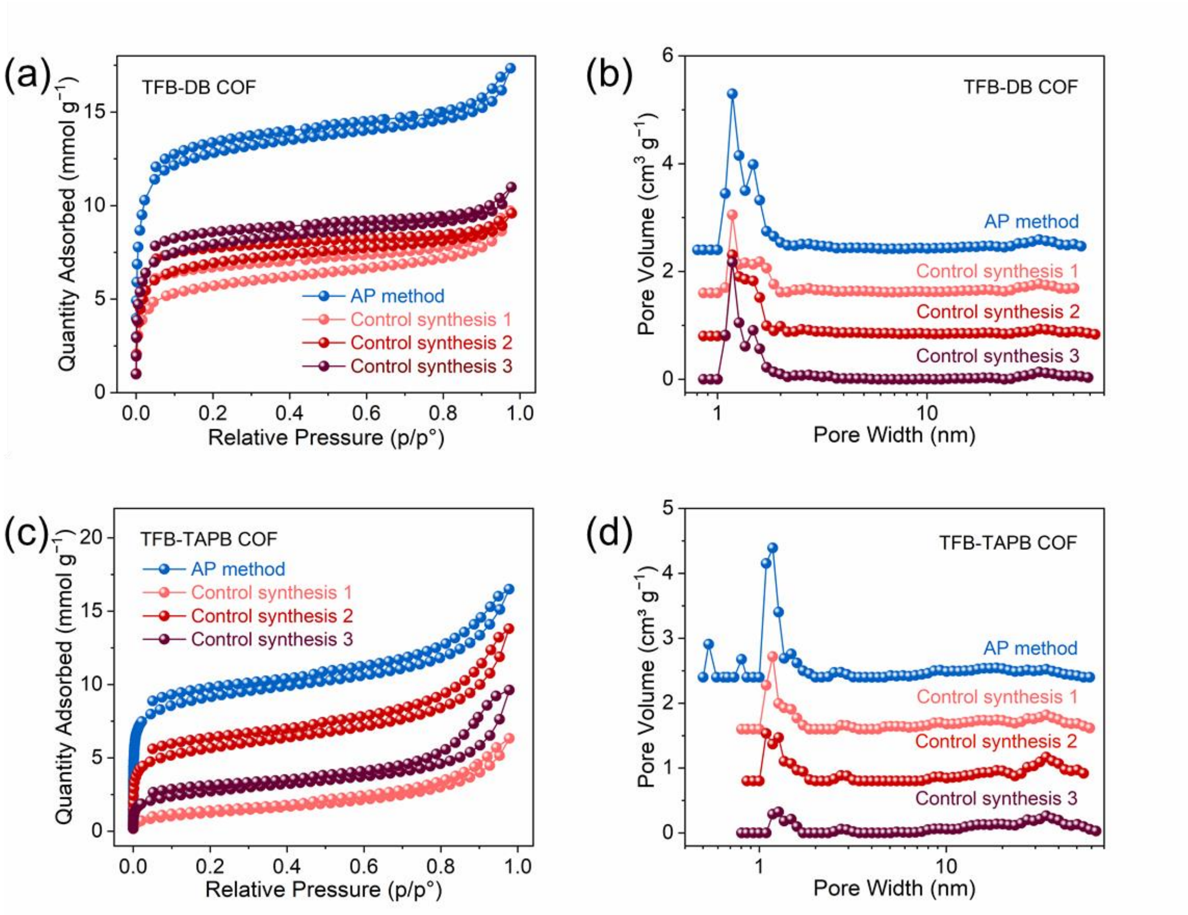

Figure S10. N_2 adsorption-desorption isotherms and pore size distribution analysis of TFB-DB COF (a-b) and TFB-TAPB COF (c-d). The COFs were synthesized using different methods at room temperature with a reaction time of 2 h.

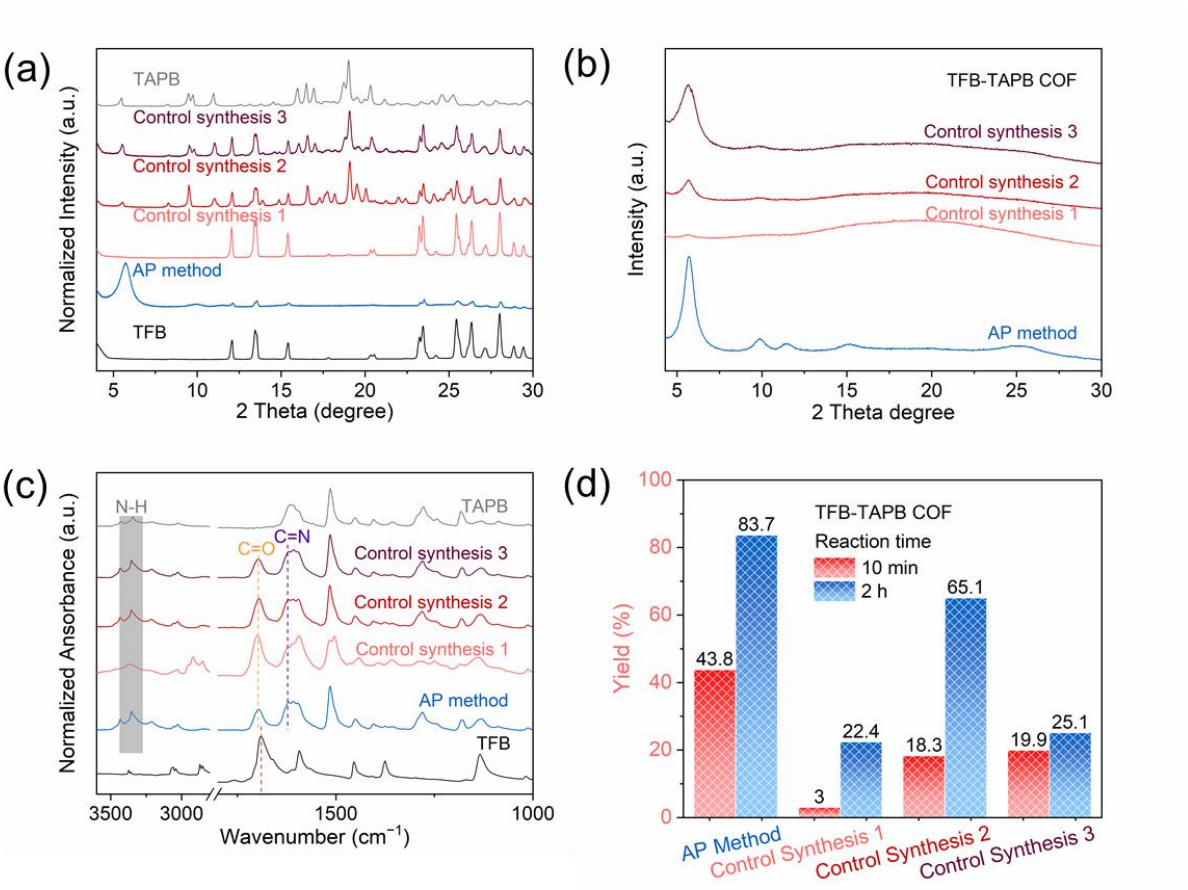

Figure S11. (a) Comparison of XRD patterns of the TFB monomer and the crude TFB-TAPB COF samples synthesized by different methods at room temperature with a reaction time of 10 min. The samples were washed with water to remove acetic acid prior to the measurements. (b) XRD patterns of the purified TFB- TAPB COF samples synthesized by different methods at room temperature with a reaction time of 10 min. The samples were thoroughly washed by water and acetone to remove acetic acid and any unreactive monomers prior to the measurements; (c) Comparison of IR spectra of the TFB and TAPB monomers with the purified TFB-TAPB COF samples synthesized by different methods at room temperature with a reaction time of 10 min (d) Comparison of the yield of TFB- TAPB COFs synthesized by different methods at room temperature with a reaction time of 10 min and 2 h.

As shown in Figure S11a, the crude product collected from the Control syntheses 1 at 10 min only displayed strong diffraction peaks for the TFB monomer and the samples collected from the Control syntheses 2 and 3 at 10 min displayed strong diffraction peaks for the TFB monomer and TAPB monomer. No significant diffraction peaks for TFB-TAPB COF were observed, indicating the low yield and weak crystallinity of the products obtained from the

control syntheses. In contrast, the crude product obtained from the AP synthesis clearly showed the characteristic diffraction peak at 5.8° of the (001) plane of TFB-TAPB COF.

In addition, the purified sample obtained from the AP synthesis had significantly higher crystallinity than those of the samples synthesized under control synthesis conditions. Control synthesis 1 gave the COF products with the lowest yields (3 % at 10 min and 22.4 % at 2 h) and the poorest crystallinity among all syntheses.

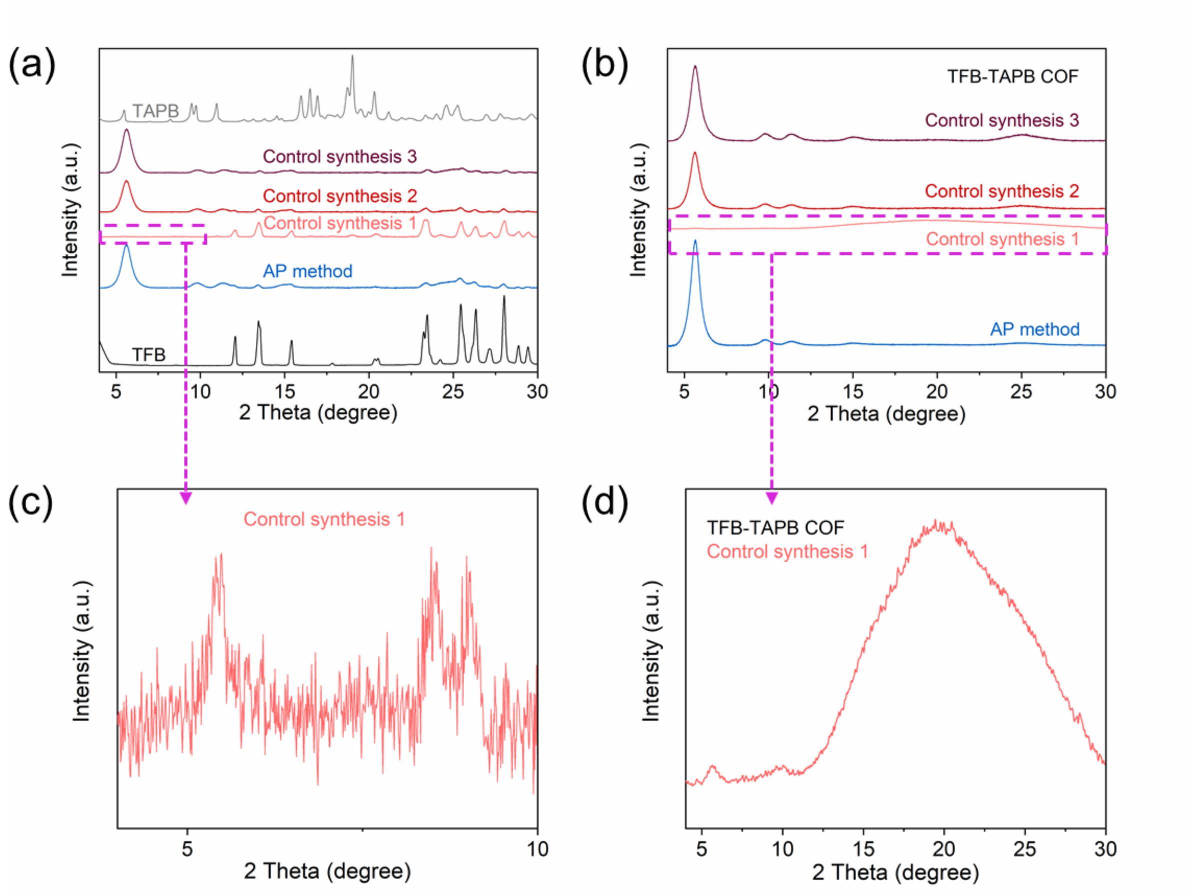

Figure S12. (a) Comparison XRD patterns of the collected TFB-TAPB COF samples synthesised under AP method and control conditions (reaction time: 2 h). The samples were washed with water to remove acetic acid prior to the measurements; (b) Comparison XRD patterns of the collected TFB-TAPB COF samples synthesized under AP method and control conditions (reaction time: 2 h). The samples were purified by washing with both water and acetone to remove acetic acid and any unreactive monomers prior to the measurements; (c) zoom in the XRD pattern of TFB-TAPB COF synthesized under Control synthesis 1 showed in Figure S12a; (d) zoom in the XRD pattern of TFB-TAPB COF synthesized under Control synthesis 1 showed in Figure S12b.

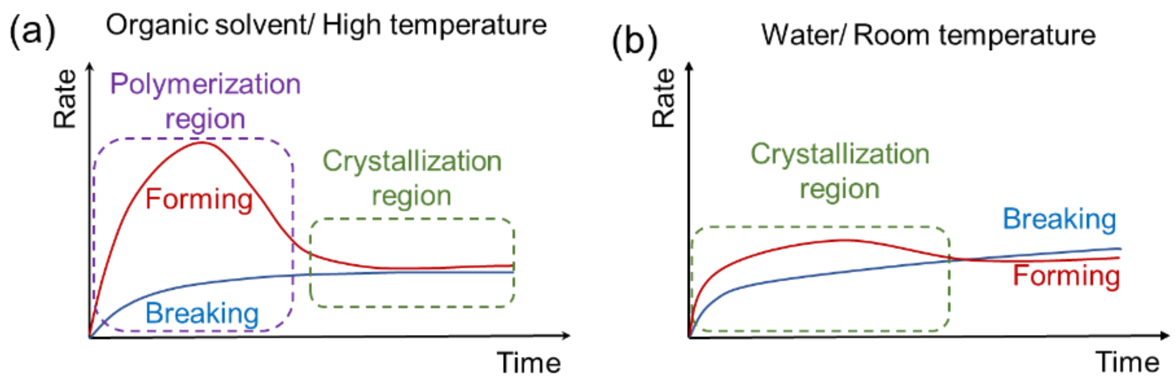

Figure S13. Proposed reaction and crystallization process of imine-linked COFs under different synthesis conditions.

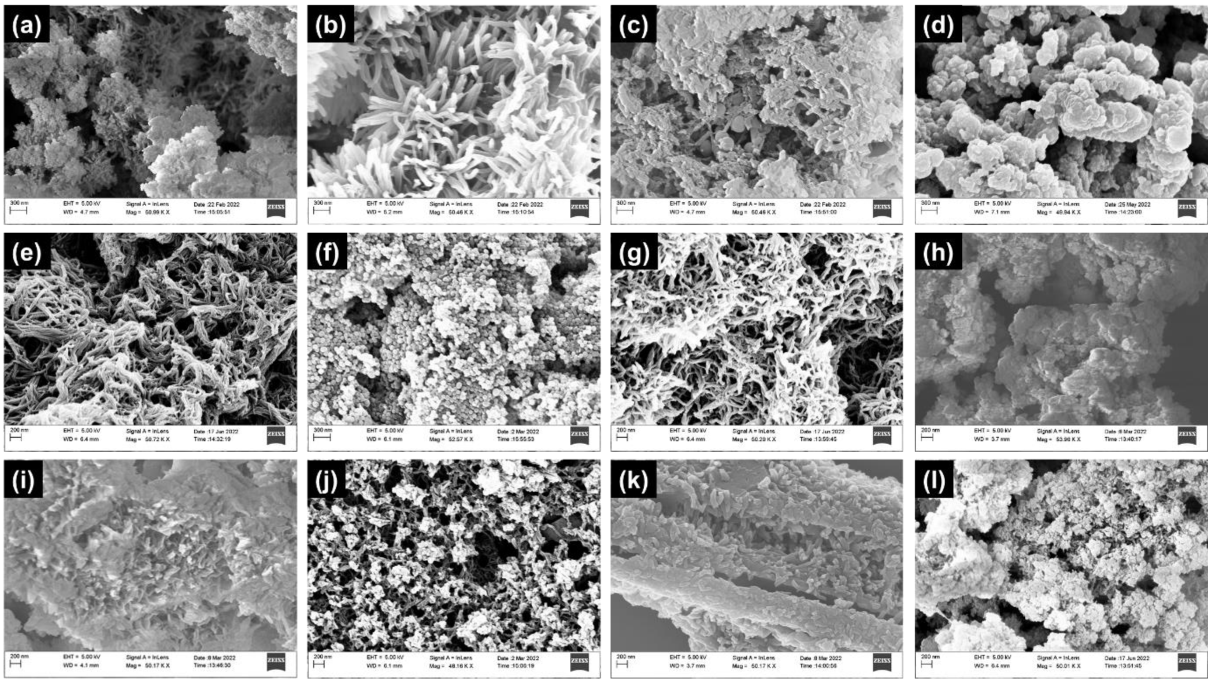

Figure S14. SEM images of TFB-BD COF (a), TFB-DDB COF (b), TFB-TAPA COF (c), TPA-TAPPA COF (d), DMTA-TAPT COF (e), DMTA-PTTA COF (f), TFPA-TAPB COF (g), TFPB-TAPA COF (h), TFPB-TAPB COF (i), TFPB-TAPT COF (j), TFPB-ETTA COF (k) and TFPT-ETTA COF (l).

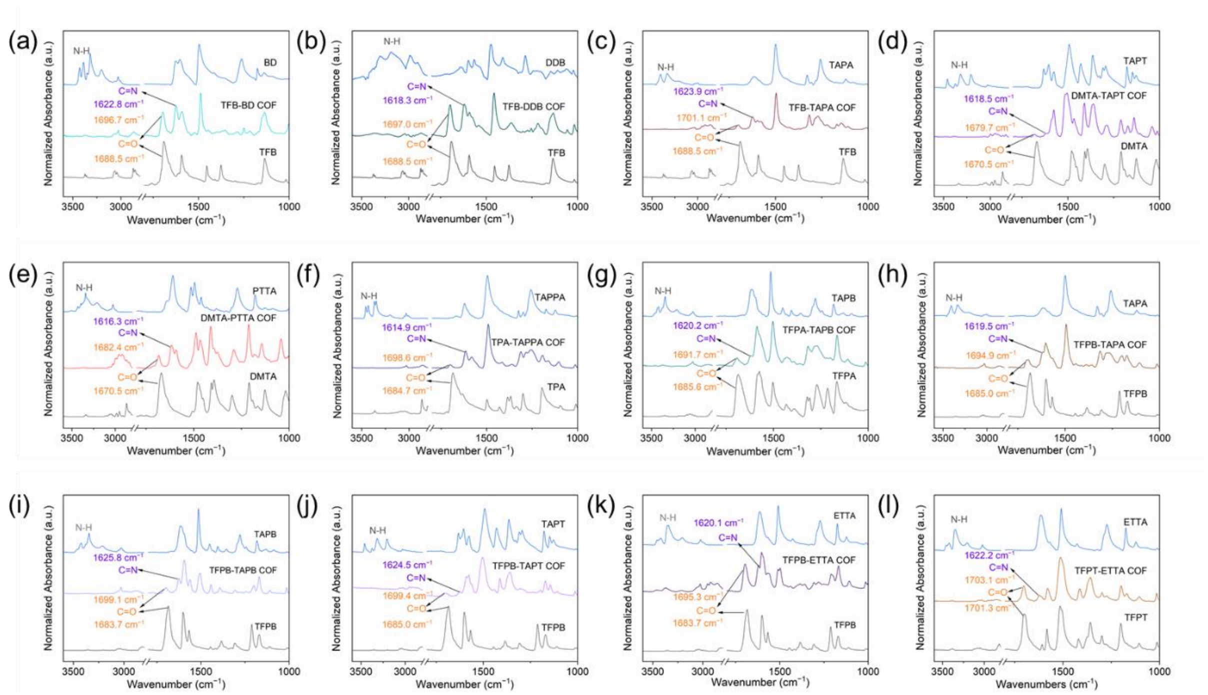

Figure S15. IR spectra of the synthesized COFs and the corresponding organic monomers. TFB-BD COF (a), TFB-DDB COF (b), TFB-TAPA COF (c), TPA-TAPPA COF (d), DMTA-TAPT COF (e), DMTA-PTTA COF (f), TFPA-TAPB COF (g), TFPB-TAPA COF (h), TFPB-TAPB COF (i), TFPB-TAPT COF (j), TFPB-ETTA COF (k) and TFPT-ETTA COF (l).

All synthesized COF showed intense IR peaks at 1614.9-1625.8 cm^{-1}, which indicated the formation of imine bonds. Compared to the strong C=O vibrations at 1670.5-1701.3 cm^{-1} for the aldehyde monomers, the COFs showed significantly reduced intensity of C=O vibrations due to the consumption of the aldehyde group during the Schiff-base condensation and the relatively high polymerization degree of COFs.

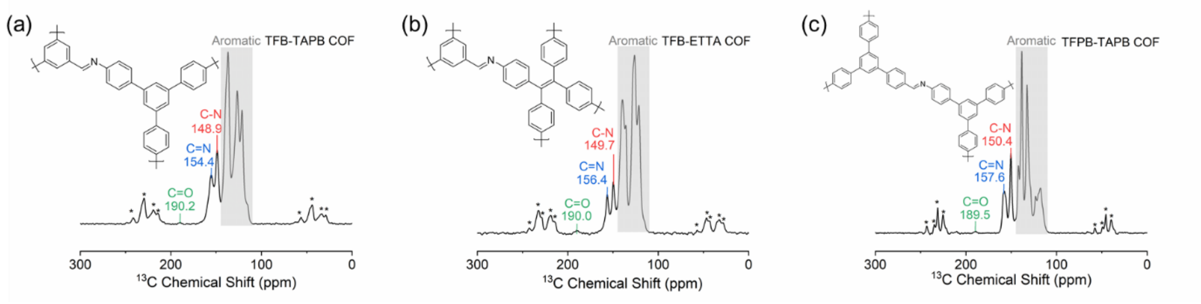

Figure S16. Solid-state ^{13}C NMR spectroscopy of the synthesized COFs and the corresponding composites. TFB-TAPB COF (a), TFB-ETTA COF (b), and TFPB-TAPB COF (c).

In addition to infrared spectroscopy, we have utilized solid-state ^{13}C NMR spectroscopy to analyze the molecular structure of the COFs. As displayed in Figure S16, the solid-state ^{13}C NMR spectra of TFB-TAPB COF, TFB-ETTA COF, and TFPB-TAPB COF display intense peaks with chemical shifts in the range of 110-142 ppm, corresponding to aromatic carbons. The distinctive peak at ~155 ppm is attributed to the carbon atoms in imine bonds. Furthermore, a subtle peak at ~190 ppm is identified as originating from the carbon atoms of aldehyde end groups.

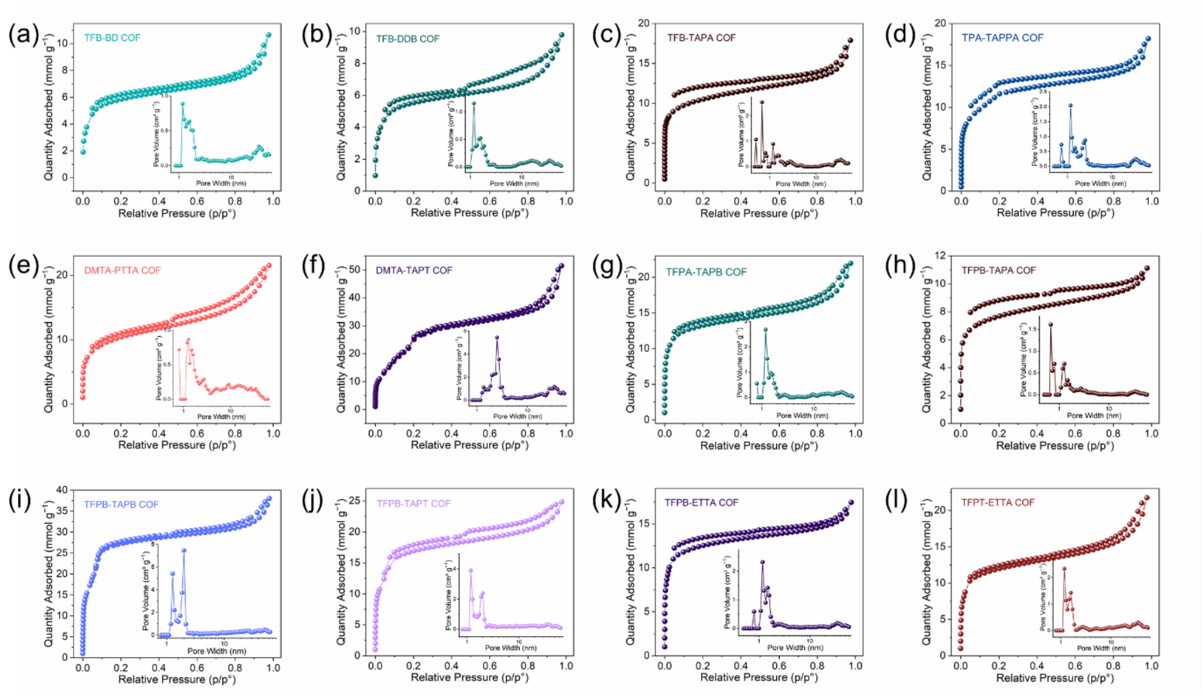

Figure S17. N_2 adsorption-desorption isotherms of TFB-BD COF (a), TFB-DDB COF (b), TFB-TAPA COF (c), TPA-TAPPA COF (d), DMTA-TAPT COF (e), DMTA-PTTA COF (f), TFPA-TAPB COF (g), TFPB-TAPA COF (h), TFPB-TAPB COF (i), TFPB-TAPT COF (j), TFPB-ETTA COF (k) and TFPT-ETTA COF (l). The insert figures display the pore size distributions of the COFs.

The porosity of the COFs was measured by nitrogen sorption at 77 K (Figure S17). The COFs displayed either type I or type IV isotherms. All of the isotherms showed rapid N_2 uptake at low relative pressures ($p/p_0<0.01$), indicating the formation of micropores in the COFs. The BET surface area of TFB-BD COF, TFB-DDB COF, TFB-TAPA COF, TPA-TAPPA COF, DMTA-TAPT COF, DMTA-PTTA COF, TFPA-TAPB COF, TFPB-TAPA COF, TFPB-TAPB COF, TFPB-TAPT COF, TFPB-ETTA COF, and TFPT-ETTA COF were calculated to be 482, 459, 848, 923, 1994, 847, 1070, 617, 2487, 1468, 1010 and 965 m^2 g^{-1}, respectively.

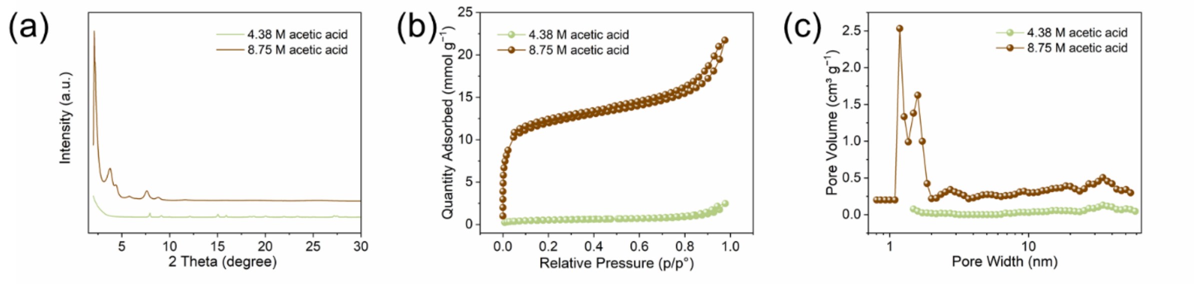

Figure S18. (a) XRD patterns of TFPT-ETTA COF synthesized from the aqueous solution with different concentrations of acetic acid; (b) N_2 adsorption-desorption isotherms of the obtained TFPT-ETTA COFs and their (c) pore size distributions.

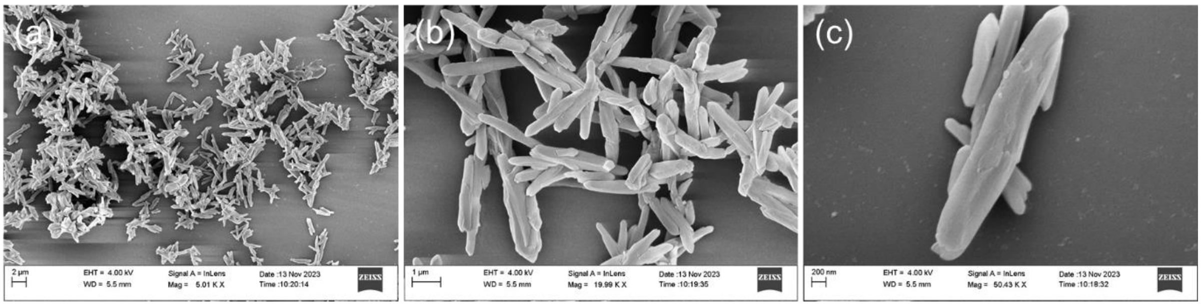

Figure S19. SEM images of TPA-TAPM COF.

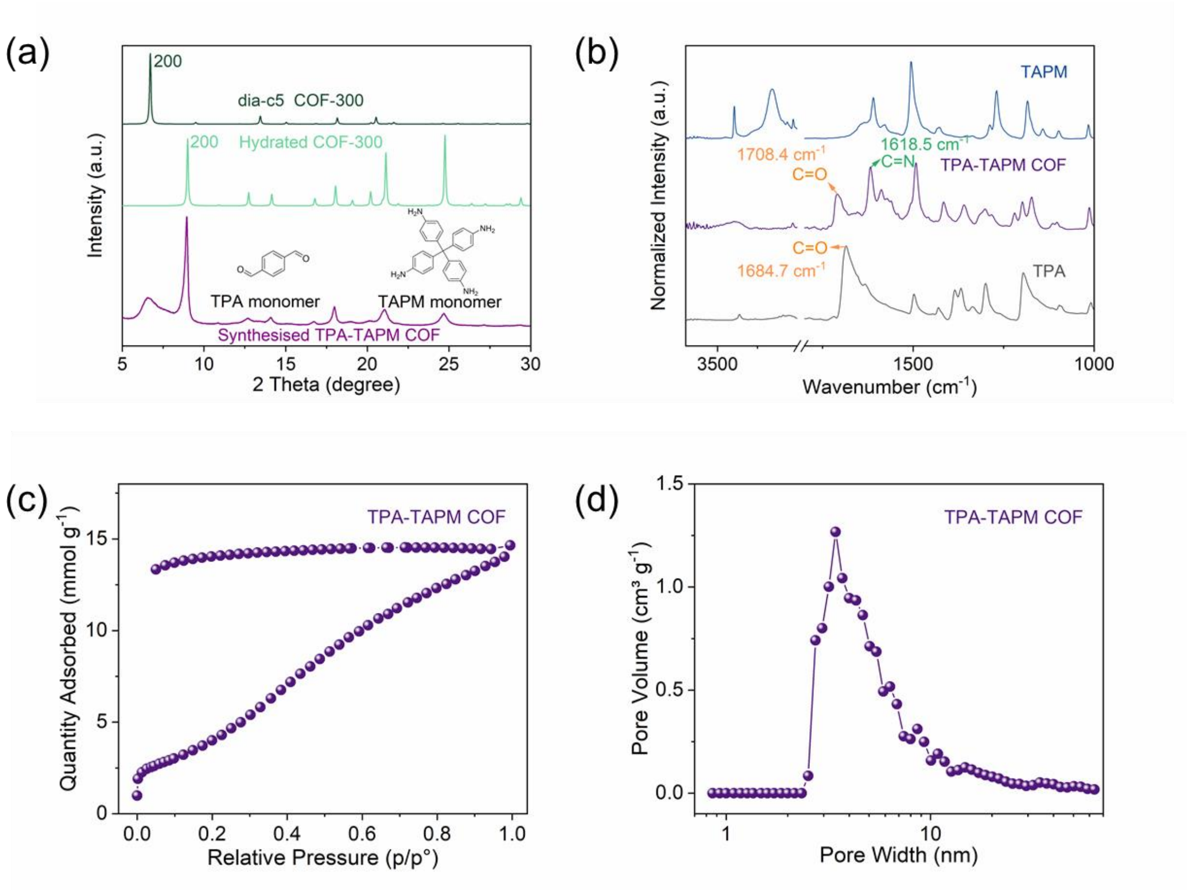

Figure S20. (a) XRD pattern, (b) IR spectrum, (c) N_2 adsorption-desorption isotherms, and (d) pore size distributions of TPA-TAPM COF.

We have attempted to synthesize 3D COFs by reacting tetrakis(4-aminophenyl)methane (TAPM) with different aldehyde monomers including TFB and TPA. Interestingly, the reaction between TAPM and TPA using our developed aqueous synthesis approach led to the formation of crystalline COF-300 (Figure S19 and S20); however, two different crystalline phases (dia-c5 COF-300 and the hydrated form of COF-300) were identified in the obtained materials based on the XRD studies (Figure S20a). Previous studies indicate that dia-c5 COF-300 exhibits a diamond (dia) topology with a 5-fold interpenetrated framework, giving a high surface area of 1360 m^2/g and rich microporosity.[4] In contrast, the hydrated form of COF-300 represents an isomer of dia-c5 COF-300 with a distorted structure and is nonporous.[5] The COF-300 sample obtained in our study displays a moderate surface area of 305 m^2/g, which is consistent with the fact that it contains mixed phases of the porous dia-c5 COF-300 and the hydrated form.

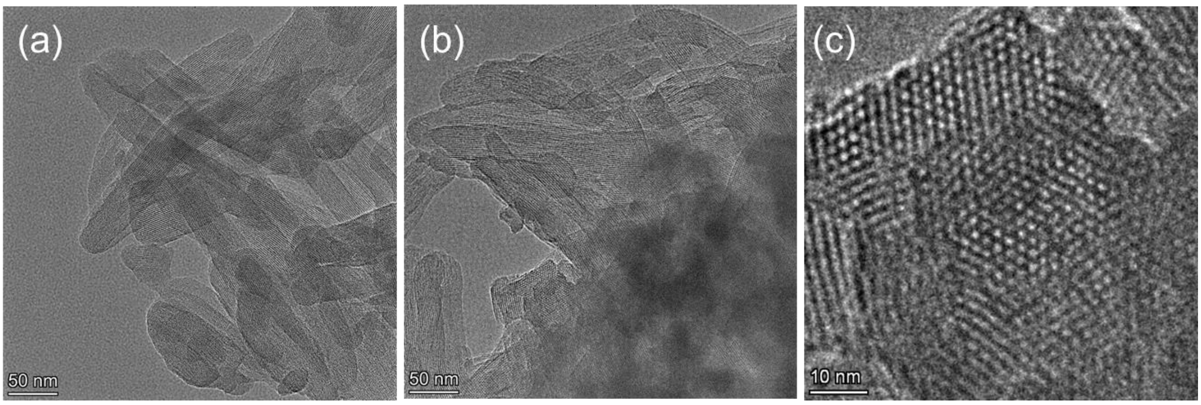

Figure S21. High-resolution transmission electron microscopy (HRTEM) images of TFPB-TAPB COF.

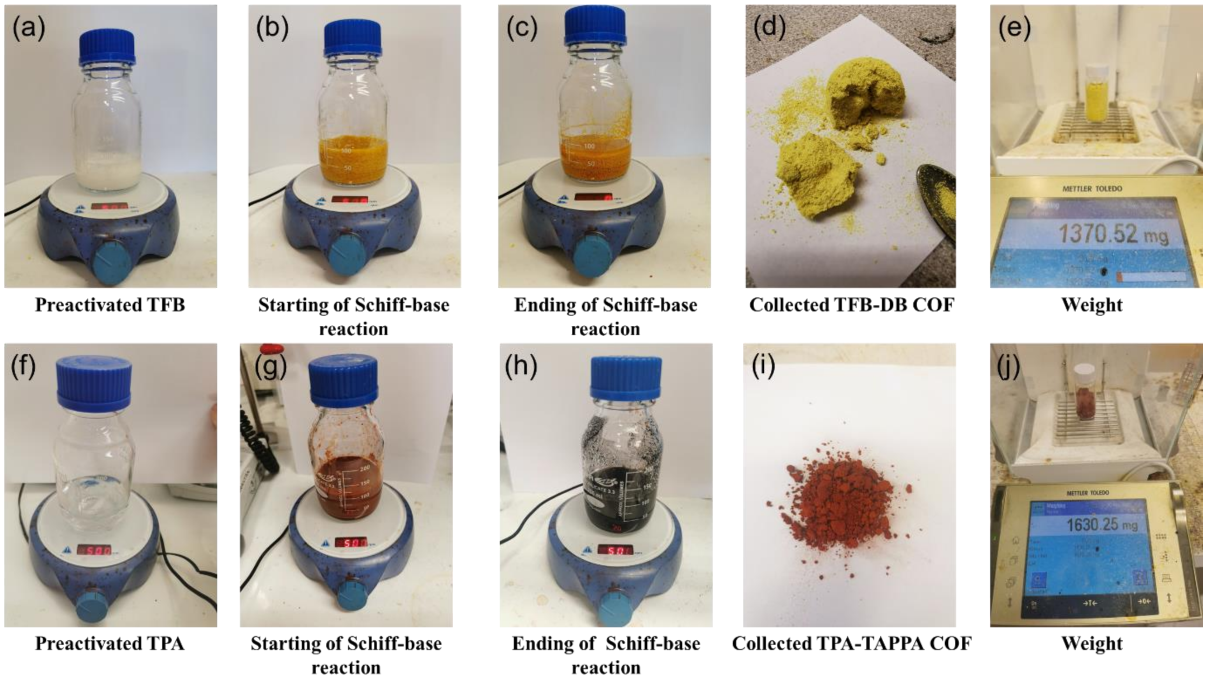

Figure S22. Optical images showing the gram-scale synthesis of TFB-DB COF (a-e) and TPA-TAPPA COF (f-j).

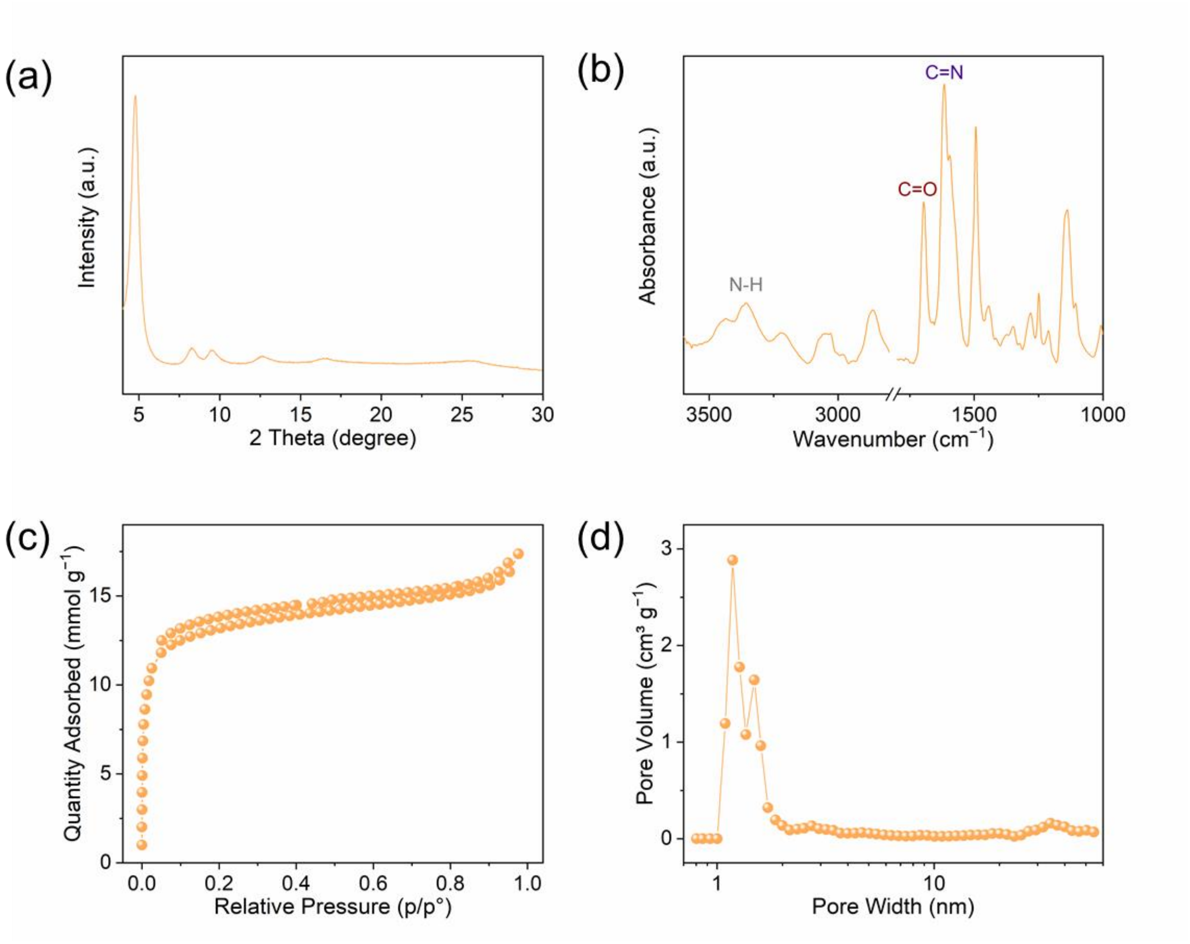

Figure S23. (a) XRD pattern, (b) IR spectrum, (c) N_2 adsorption-desorption isotherms, and (d) pore size distribution of TFB-DB COF obtained from the gram scale synthesis.

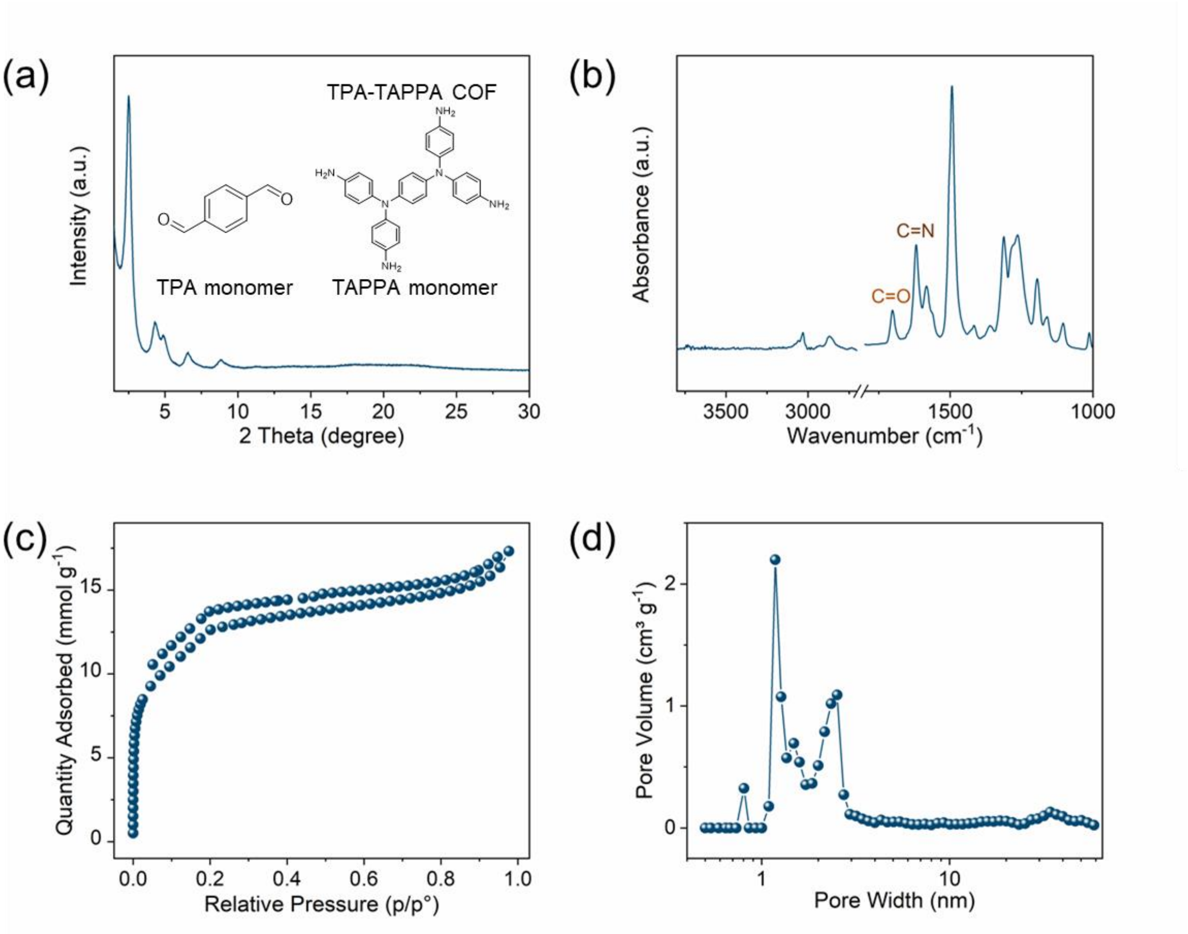

Figure S24. (a) XRD pattern, (b) IR spectrum, (c) N_2 adsorption-desorption isotherms, and (d) pore size distribution of TPA-TAPPA COF obtained from the gram scale synthesis.

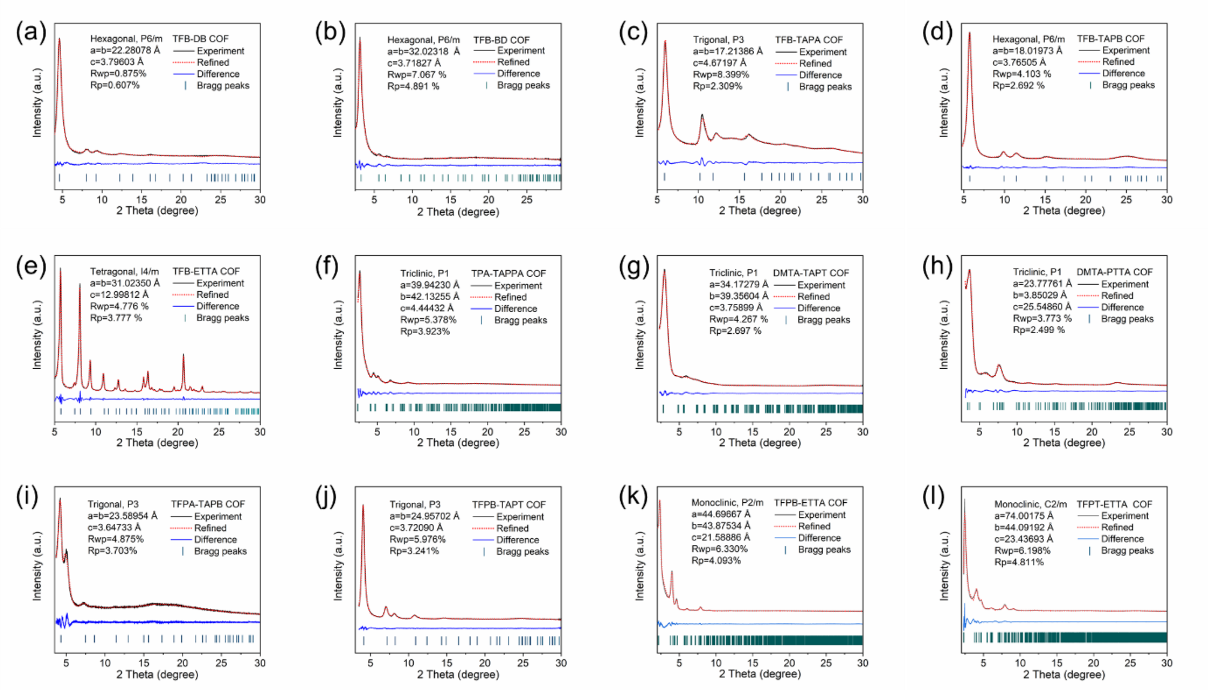

Figure S25. PXRD pattern and Pawley refinement of TFB-DB COF (a), TFB-BD COF (b), TFB-TAPA COF (c), TFB-TAPB COF (d), TFB-ETTA COF (e), TPA-TAPPA COF (f), DMTA-TAPT COF (g), DMTA-PTTA COF (h), TFPA-TAPB COF (i), TFPB-TAPT COF (j), TFPB-ETTA COF (k), and TFPT-ETTA COF (l) (all samples were synthesized with a same reaction time of 72 h).

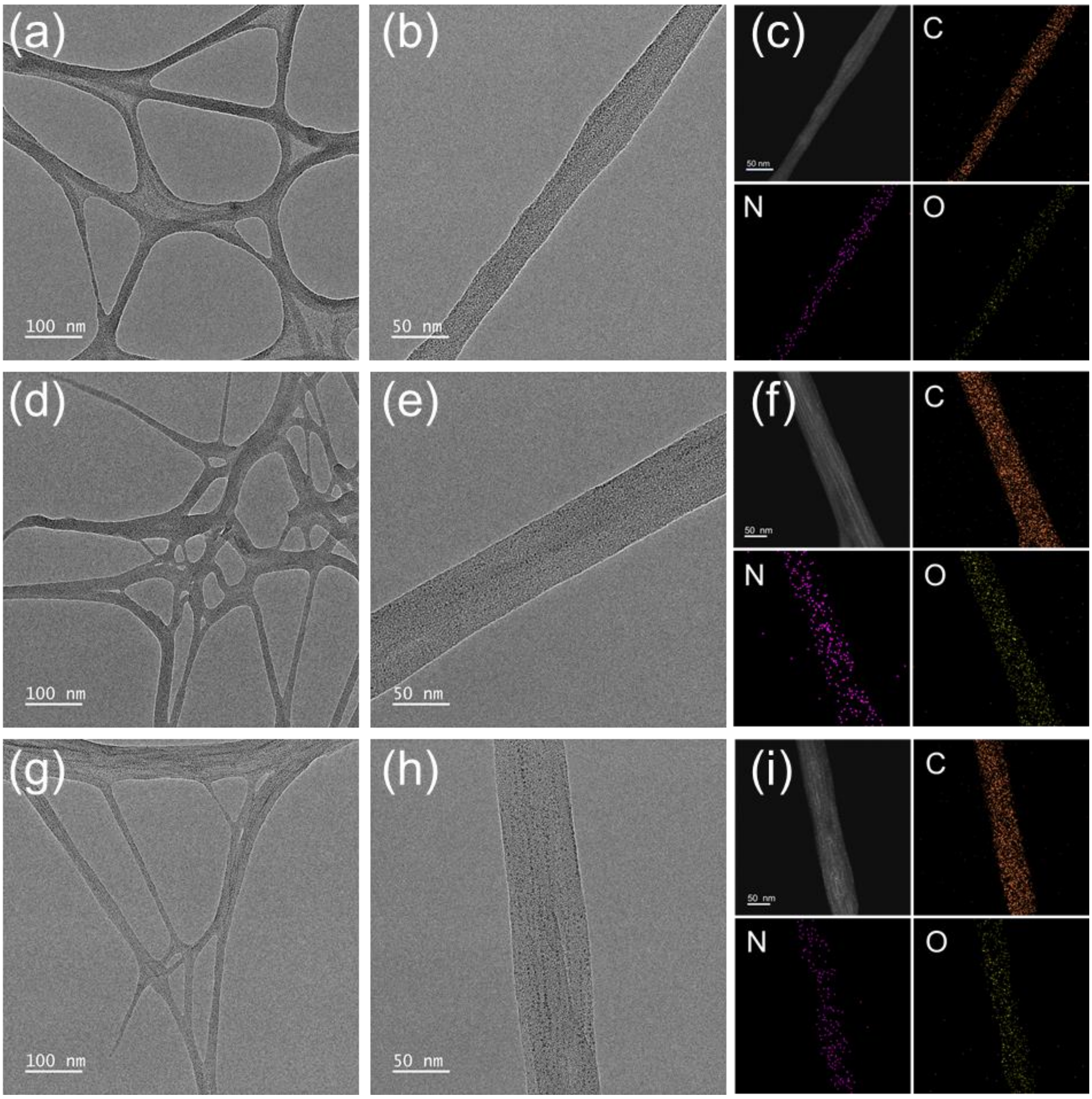

Figure S26. TEM and EDX mapping images of the synthesized CNF@TFB-DB COF (a-c), CNF@TFB-TAPB COF (d-f), and CNF@TFB-ETTA COF (g-i).

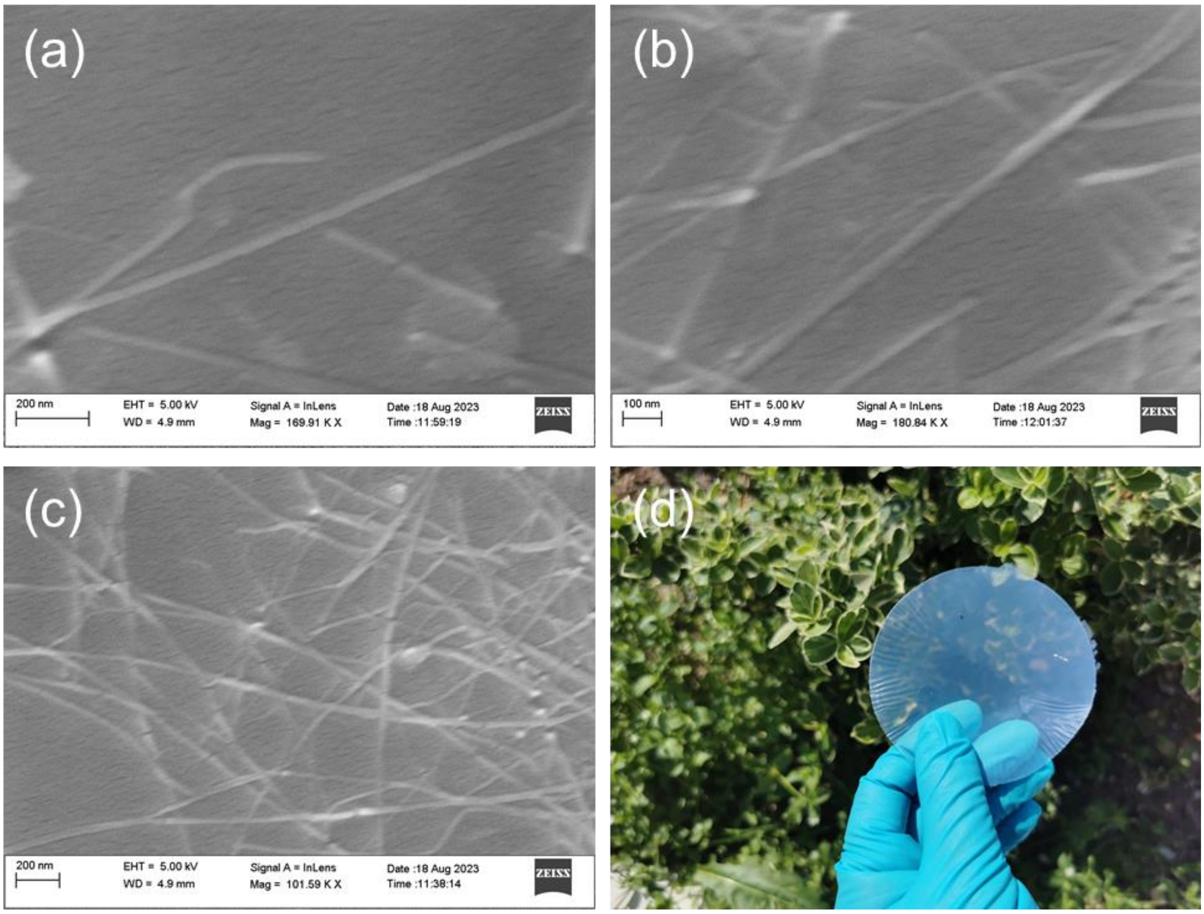

Figure S27. (a-c) SEM images of carboxylated CNF; (d) optical image of carboxylated CNF nanopaper.

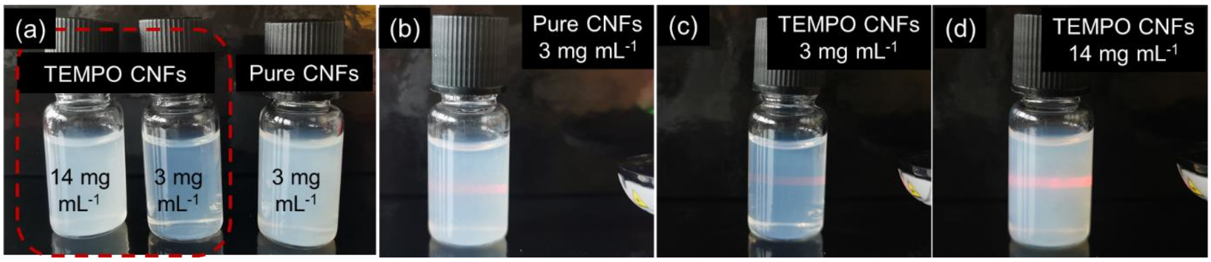

Figure S28. Optical images of aqueous suspension of unmodified CNFs (pure CNFs) and carboxylated CNFs (treated by TEMPO oxidation) with different concentrations.

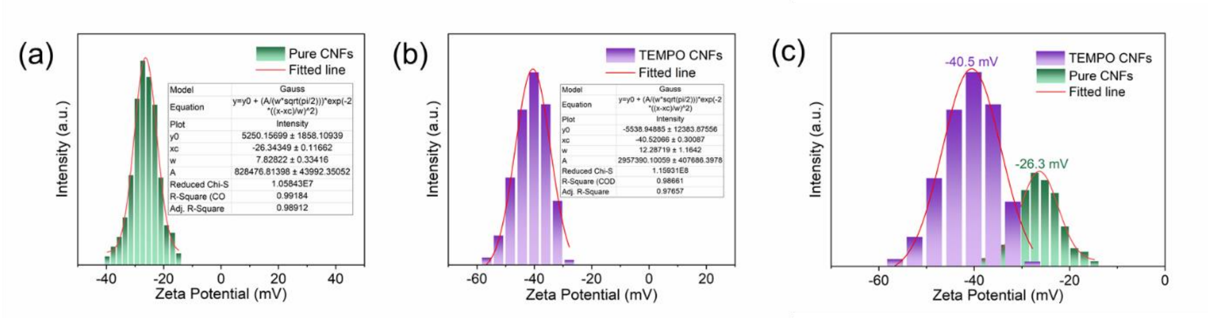

Figure S29. Zeta potential distribution of unmodified CNFs (pure CNFs) and carboxylated CNFs (treated by TEMPO oxidation). Carboxylated CNFs show a more negative zeta potential than that of unmodified CNFs, indicating an increased charge density after TEMPO oxidation treatment.

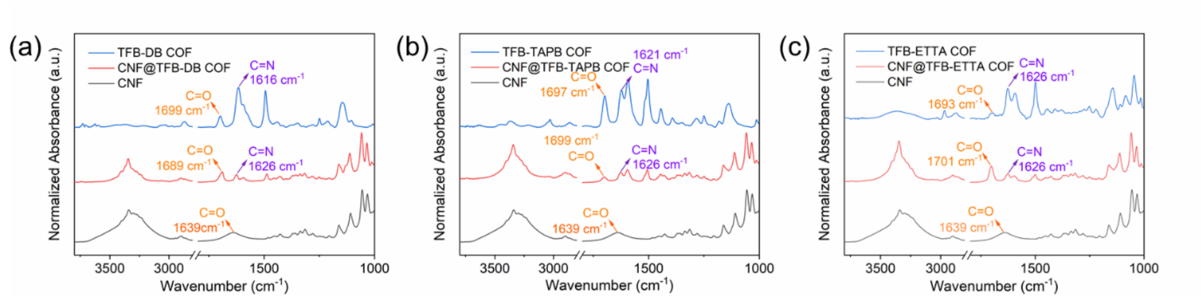

Figure S30. Infrared spectra of carboxylated CNFs, synthesized COFs and the corresponding composites of CNF@COF.

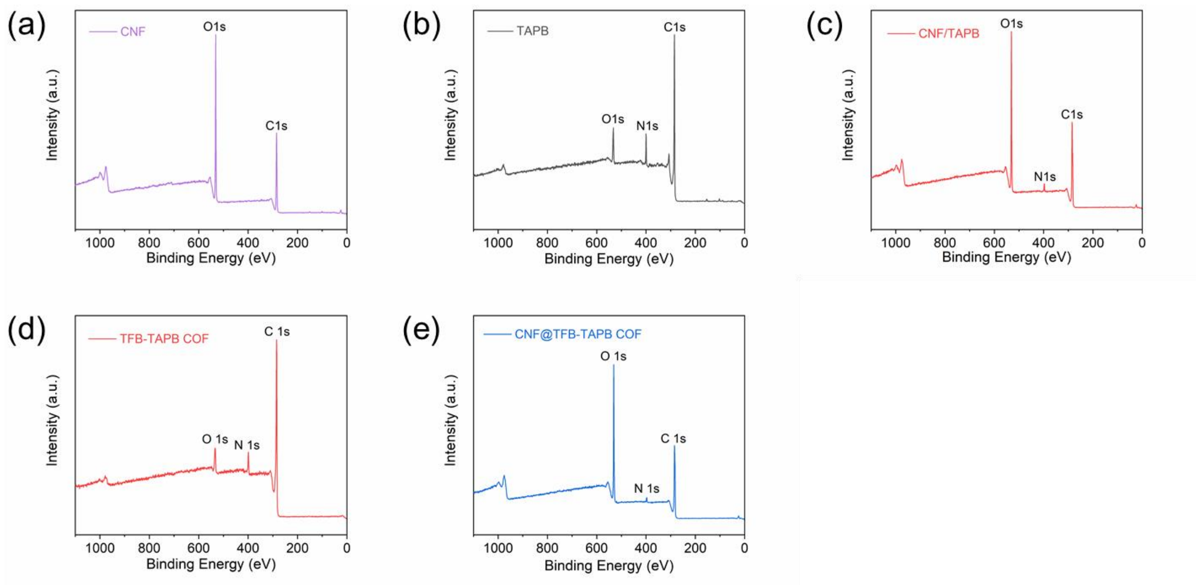

Figure S31. XPS survey spectra of CNFs, TAPB, CNF/TAPB, TFB-TAPB COF, and CNF@TFB-TAPB COF. CNFs refer to carboxylated CNFs, while CNF/TAPB denotes carboxylated CNFs treated by TAPB monomer.

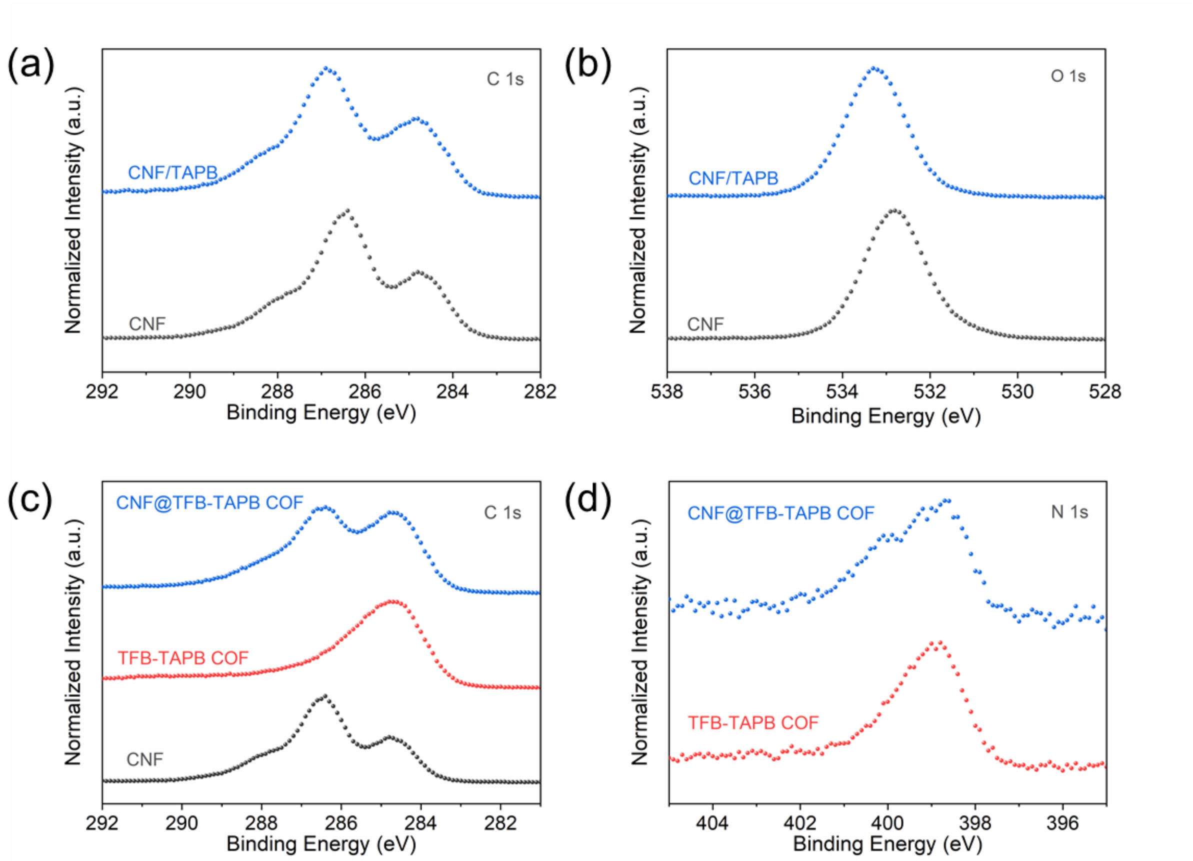

Figure S32. High-resolution X-ray photoelectron spectroscopy (XPS) spectra illustrating (a) C 1s and (b) O 1s for CNFs and CNFs treated with TAPB monomer, (c) C 1s for CNFs, TFB-TAPB COF, and CNF@TFB-TAPB COF, and (d) N 1s for TFB-TAPB COF and CNF@TFB-TAPB COF. Note: CNFs refer to carboxylated CNFs.

We propose that the synthesis of COF-CNFs is primarily driven by a templating effect, where CNF acts as a template for the gradual growth of COFs on its surface (Figure 4a). In the synthesis process, CNFs were initially mixed with amine monomers, and the carboxylate groups on the CNFs facilitated a robust interaction with the amine monomer, allowing the amine molecules to attach to the CNF surface. This interaction was confirmed by XPS studies, revealing a positive shift of 0.5 eV in the binding energy of O1s for the carboxylated CNFs upon treatment with the amine monomer (TAPB) (Figure S32). Subsequently, with the addition of aldehyde monomer into the suspension mixture of CNFs and amine monomer, polycondensation between aldehyde and amine occurred around the CNF surface, acting as a template. Eventually, COF nanolayers were successfully coated on the CNF surface. Both IR and XPS studies provided evidence of a strong interaction between CNFs and the COF nanolayer. In contrast to the pure COF samples, the C=N vibration in the IR spectra exhibited a blue shift of 5−10 cm^{-1} upon COF growth on CNFs (Figure S30). In addition to the main

peak at 399.0 eV in the N1s region for TFB-TAPB COF, a new shoulder peak emerged at 400.0 eV upon the growth of TFB-TAPB COF onto the surface of CNFs (Figure S32d). These results suggest a templating mechanism and highlight the essential role of the surface chemistry of CNFs in the synthesis of CNF@COF.

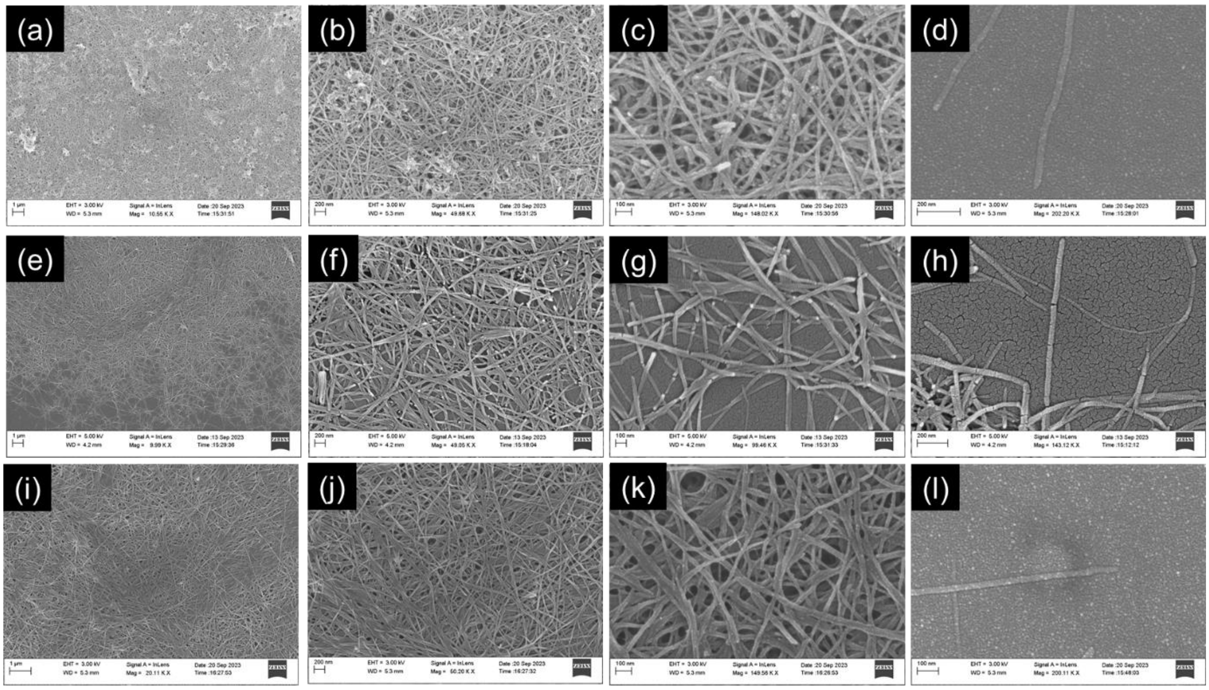

Figure S33. SEM images of the synthesized CNF@COF nanofibers. CNF@TFB-DB COF (a-d), CNF@TFB-TAPB COF (e-h), and CNF@TFB-ETTA COF (i-l).

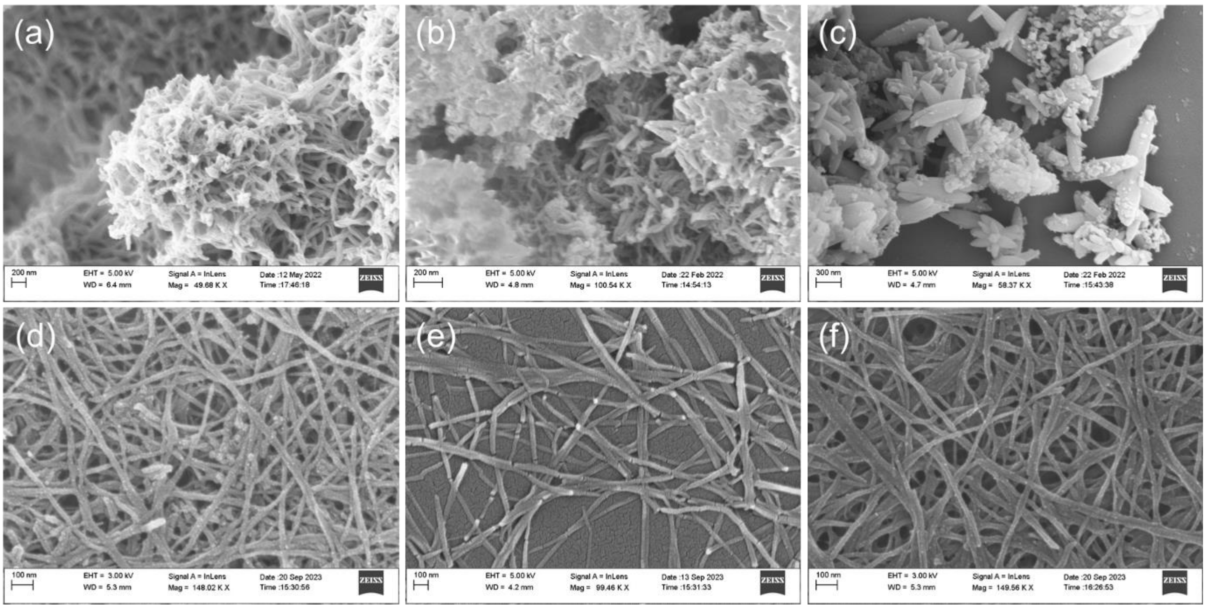

Figure S34. Comparison of SEM images of pure COFs with those of CNF@COF nanofibers. TFB-DB COF (a), TFB-TAPB COF (b), TFB-ETTA COF (c), CNF@TFB-DB COF (d), CNF@TFB-TAPB COF (e), and CNF@TFB-ETTA COF (f).

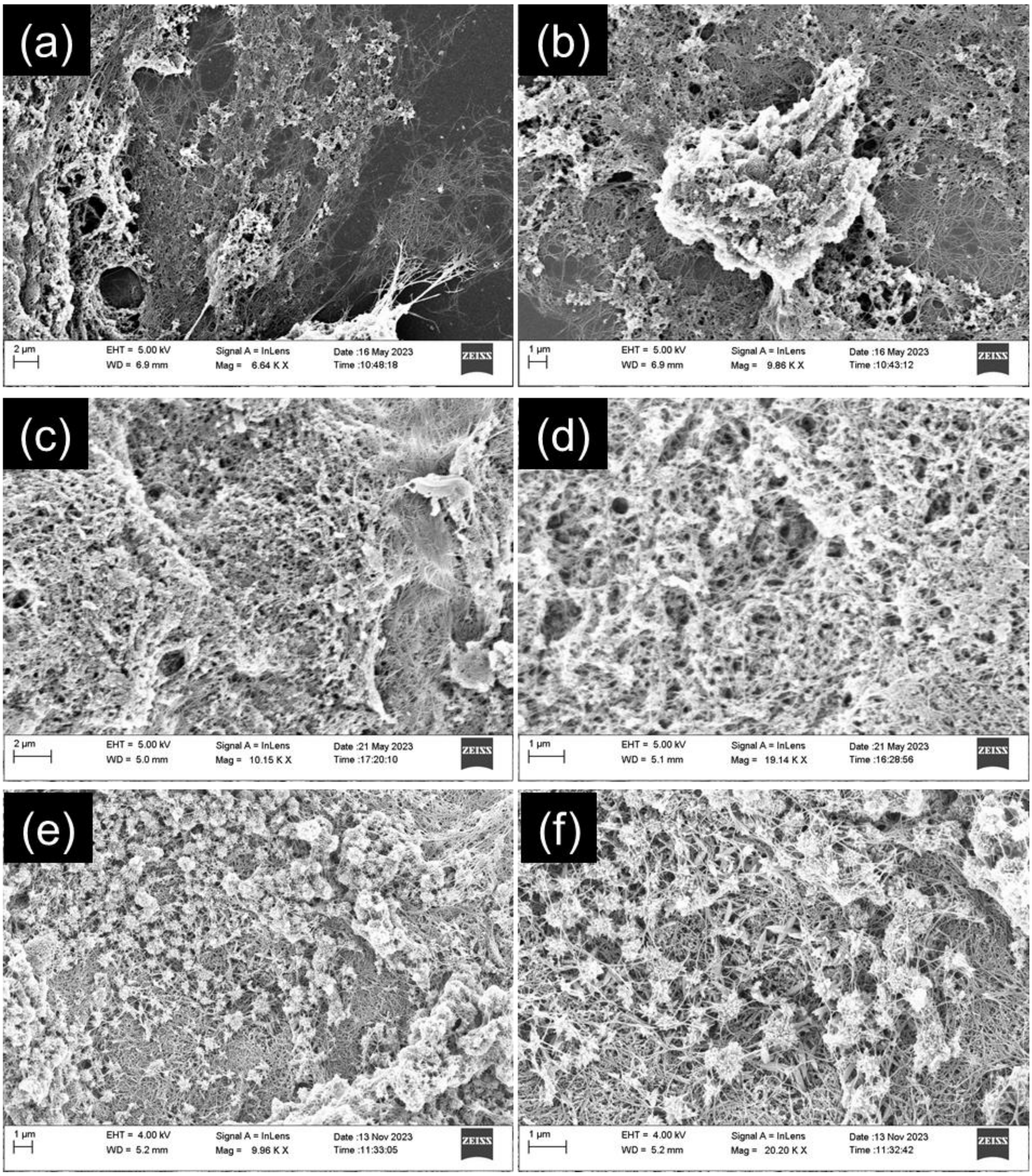

Figure S35. SEM images of CNF/TFB-DB COF (a-b), CNF/TFB-TAPB COF (c-d), and CNF/TFB-ETTA COF (e-f) synthesized using unmodified CNFs.

As shown in Figure S35, employing unmodified CNFs for the synthesis of CNF/COF led to the formation of a substantial quantity of isolated COF nanoparticles. In contrast, the use of carboxylated CNFs in the synthesis resulted in the creation of a core-shell nanofibrous structure, denoted as CNF@COF, with no significant observation of isolated COF nanoparticles (see Figures S33-S34). The observed differences in morphology in the

composites can be attributed to the distinct dispersibility between unmodified CNFs and carboxylated CNFs in aqueous solution. As shown in Figure S28, the aqueous suspension of carboxylated CNFs with a concentration of 3 mg/mL is nearly transparent, while the aqueous suspension of unmodified CNFs with the same CNF concentration is more turbid. Carboxylated CNFs possess a high surface charge density (Figure S29), resulting in excellent dispersibility in water, facilitating the formation of homogeneous CNF@COFs hybrid nanofibers. In contrast, the lack of surface charges in unmodified CNFs leads to strong hydrogen bonding between the fibers, causing aggregation of the nanofibers in the aqueous solution. As a result, this hinders efficient coating of COF nanolayers on CNFs and induces the formation of isolated COF nanoparticles during the COF synthesis (Figure S35). These studies demonstrate the pivotal role of CNF surface chemistry in determining CNF@COFs morphology.

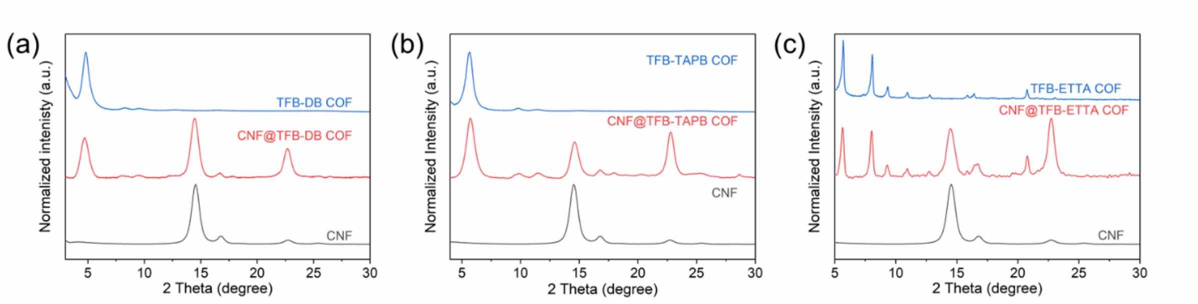

Figure S36. XRD patterns of CNFs, synthesized COFs and the corresponding CNF@COF composites.

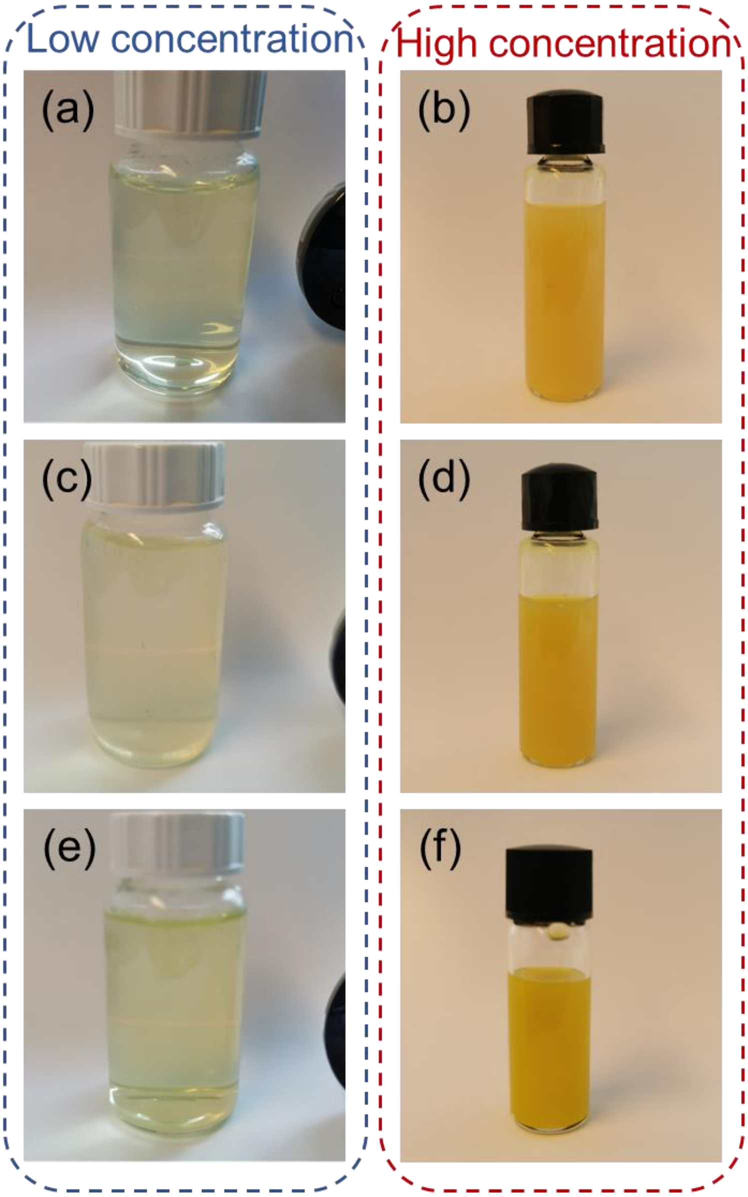

Figure S37. Optical images of CNF@COF suspensions in water: (a-b) CNF@TFB-DB COF; (c-d) CNF@TFB-TAPB COF; (e-f) CNF@TFB-ETTA COF.

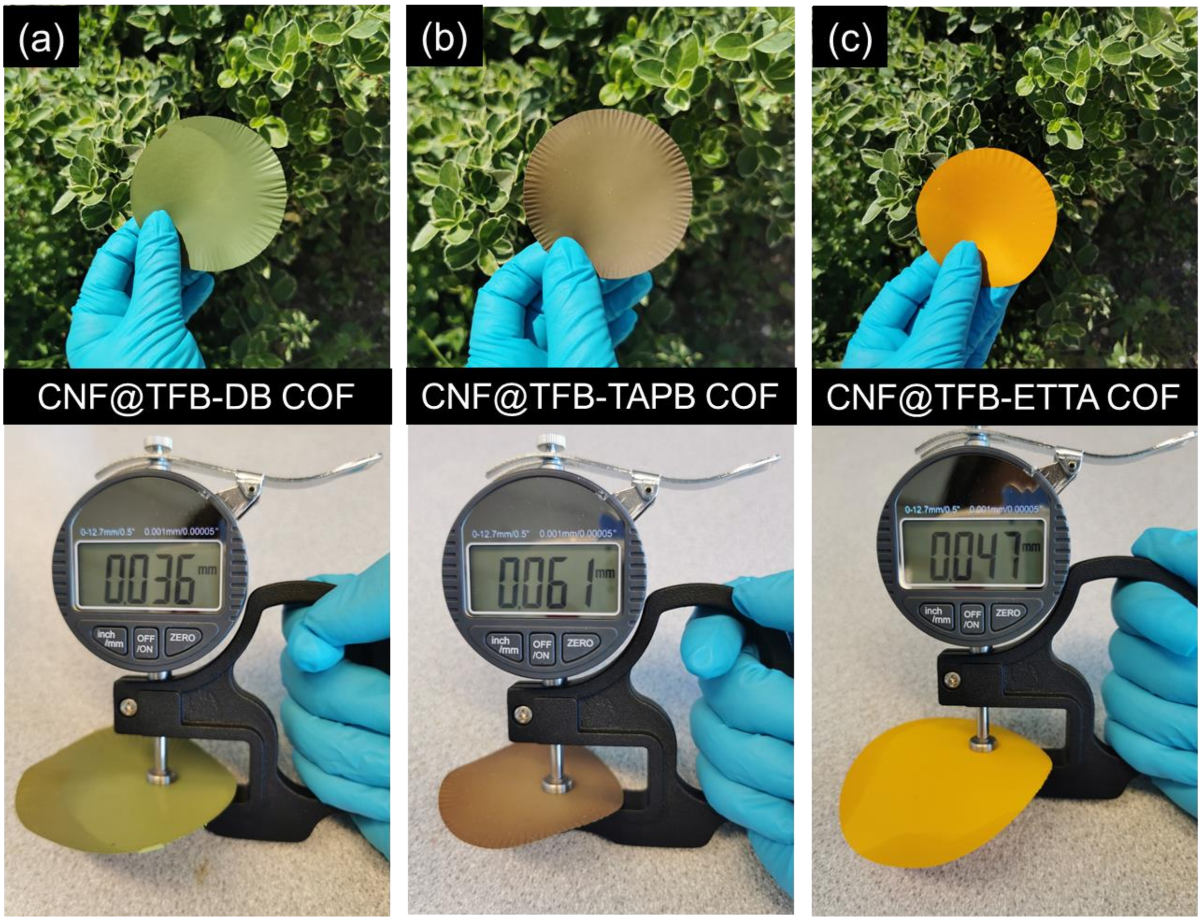

Figure S38. Optical images of freestanding CNF@TFB-DB COF nanopaper (a), CNF@TFB-TAPB COF nanopaper (b), and CNF@TFB-ETTA COF nanopaper (c).

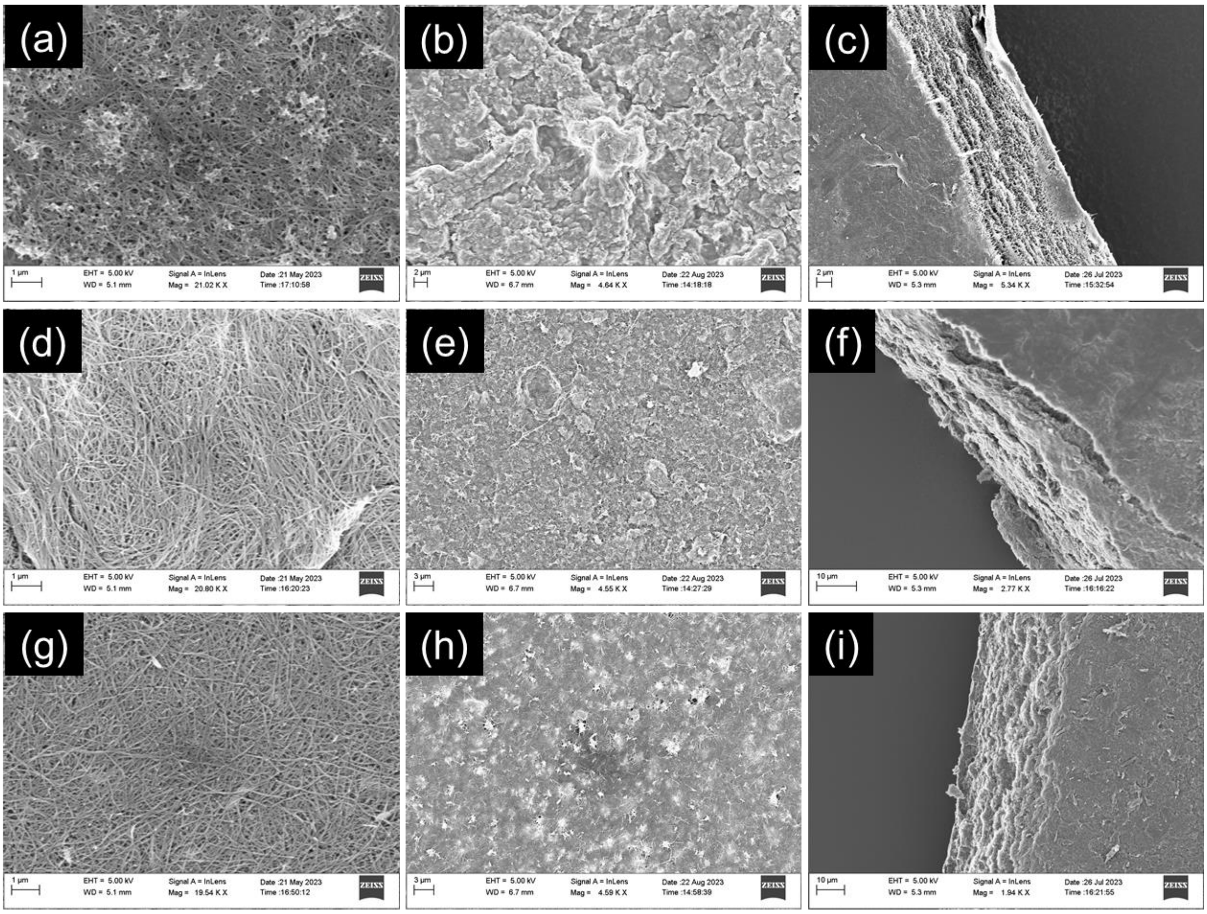

Figure S39. SEM images of the obtained CNF@COF nanopapers. CNF@TFB-DB COF nanopaper (a-c), CNF@TFB-TAPB COF nanopaper (d-f), and CNF@TFB-ETTA COF nanopaper (g-i).

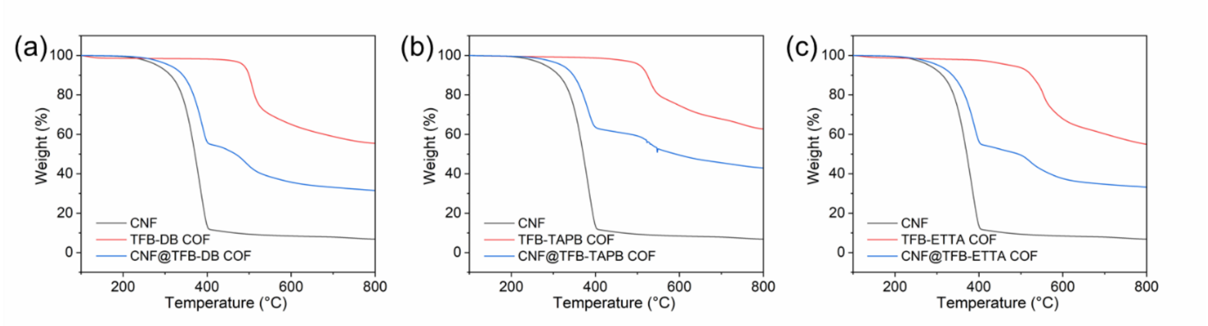

Figure S40. TGA curves of CNFs, synthesized COFs and the corresponding CNF@COF composites.

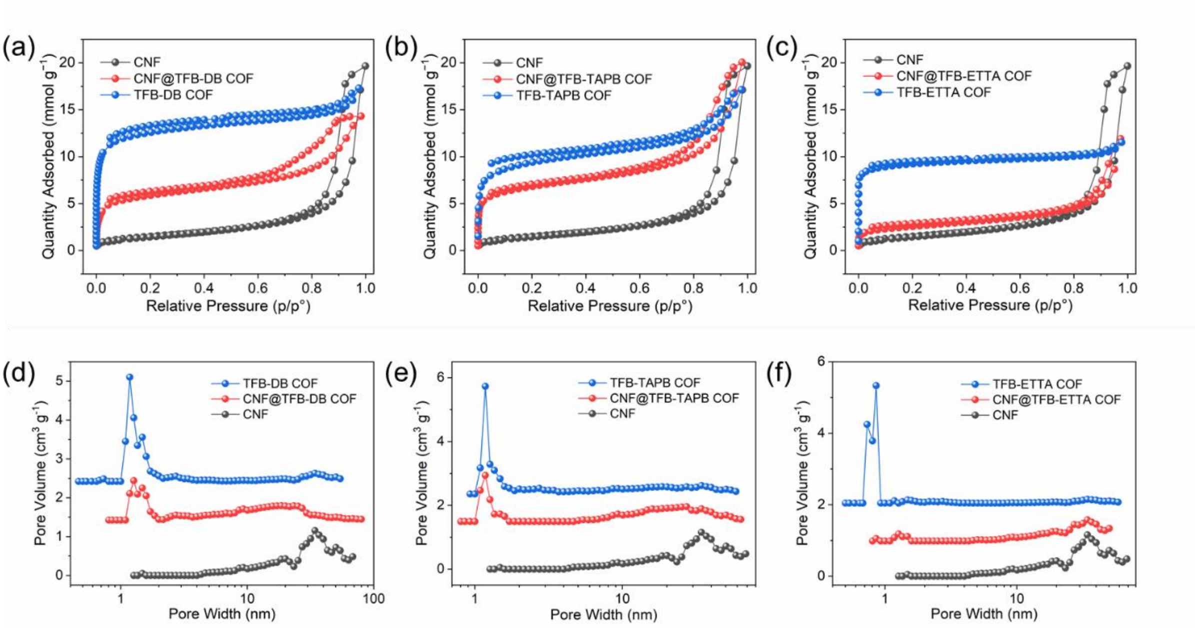

Figure S41. N_2 adsorption-desorption isotherms (a-c) and pore size distributions (d-f) of CNFs, synthesized COFs and the corresponding CNF@COF nanopapers.

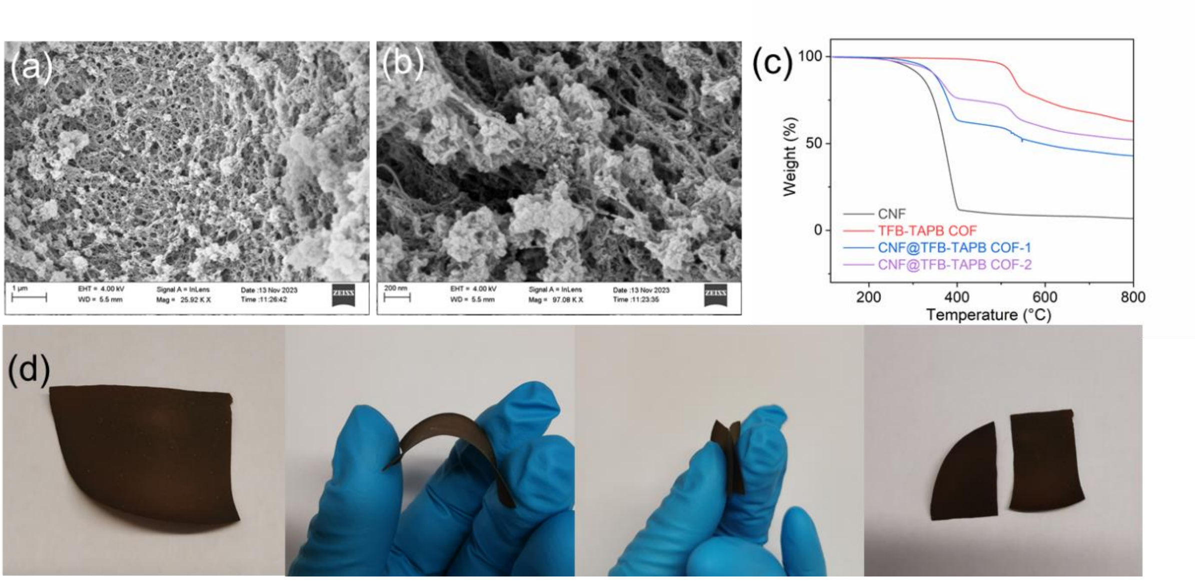

Figure S42. (a-b) SEM images of CNF@TFB-TAPB COF-2; (c) TGA curves of CNF, TFB-TAPB COF, and CNF@ TFB-TAPB COF composites. CNF@TFB-TAPB COF-2 exhibits a higher COF loading (81%) compared to CNF@TFB-TAPB COF-1 (64%). (d) Optical images illustrating the weak flexibility and foldability of CNF@TFB-TAPB COF-2 nanopaper.

We have attempted to increase the thickness of the COF coating and enhance the COF loading in CNF@COFs by adjusting the ratio between CNFs and organic monomers for COFs during the synthesis. The synthesis resulted in significantly increased COF loading amount; however,

a substantial amount of isolated COF nanoparticles formed within the composites, and there was no increase in the thickness of the COF nanolayer (Figure S42a-S42b). This result can be attributed to the limited surface area and the limited density of carboxylate groups on the CNF surface. As illustrated in Figure S42c-d, the CNF-COF nanopaper, featuring a higher TFB-TAPB COF loading amount (81 %) but comprised of isolated COF nanoparticles, demonstrated weaker flexibility in comparison to the nanopapers made from CNF@TFB-TAPB COF-1 hybrid nanofibers with a 64 % loading amount (Figure 4d).

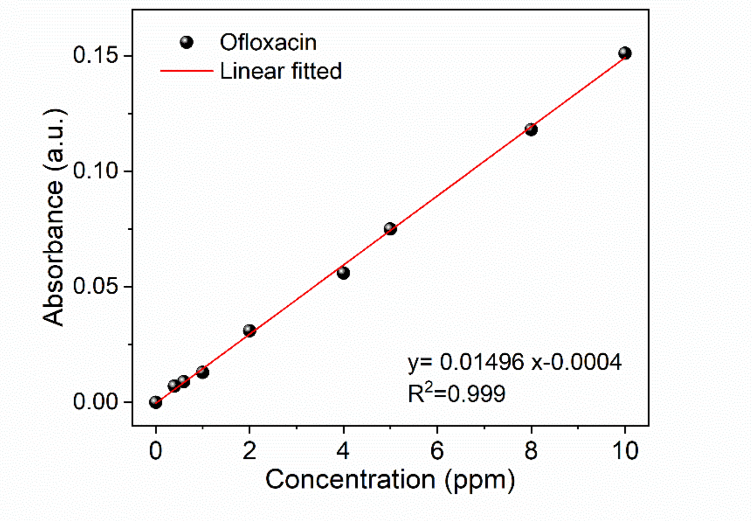

Figure S43. Calibration of OFX concentration in aqueous solutions within the ranges of 0.04−10 ppm using UV-vis spectroscopy, with absorbance at 330 nm extracted from the UV-vis spectra.

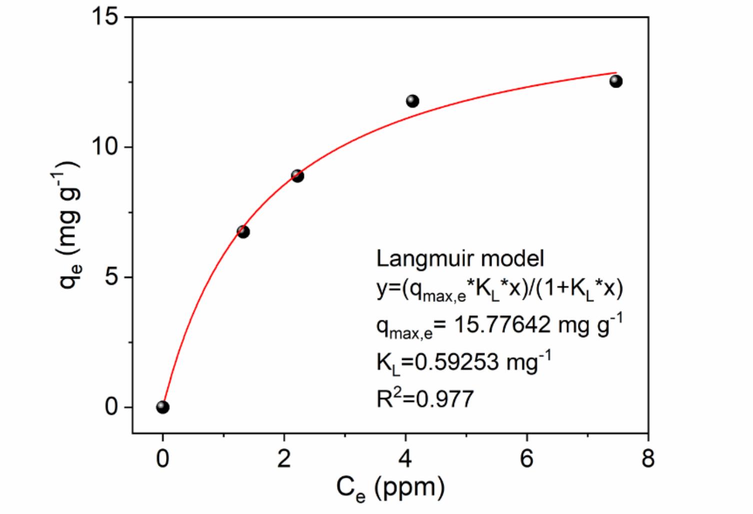

Figure S44. Ofloxacin adsorption isotherm of TFB-TAPB COF and the fitted results (red line) by using Langmuir adsorption model.

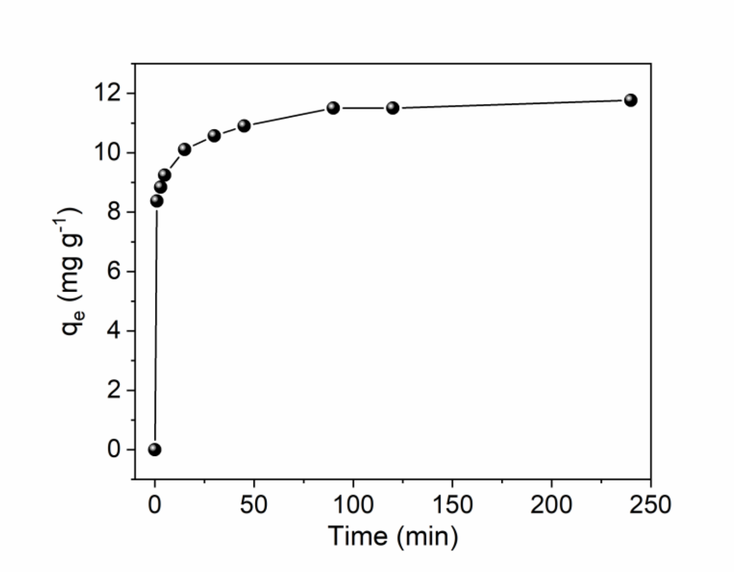

Figure S45. Kinetics of ofloxacin adsorption by TFB-TAPB COF from aqueous solutions at a concentration of 10 ppm.

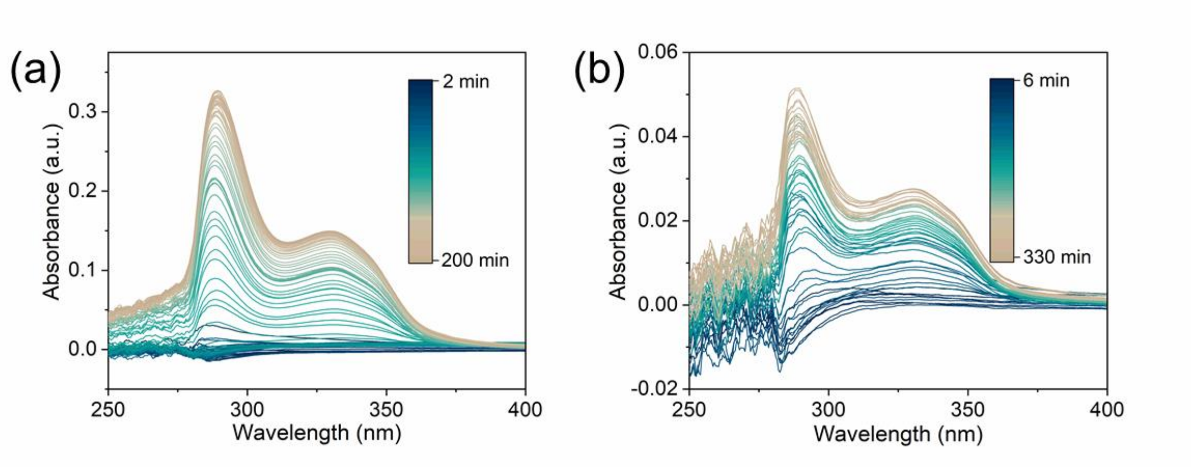

Figure S46. Time-dependent UV-vis spectra of OFX solution after treated by TFB-TAPB COF powder (a) and five CNF@TFB-TAPB COF nanopapers in series (b).

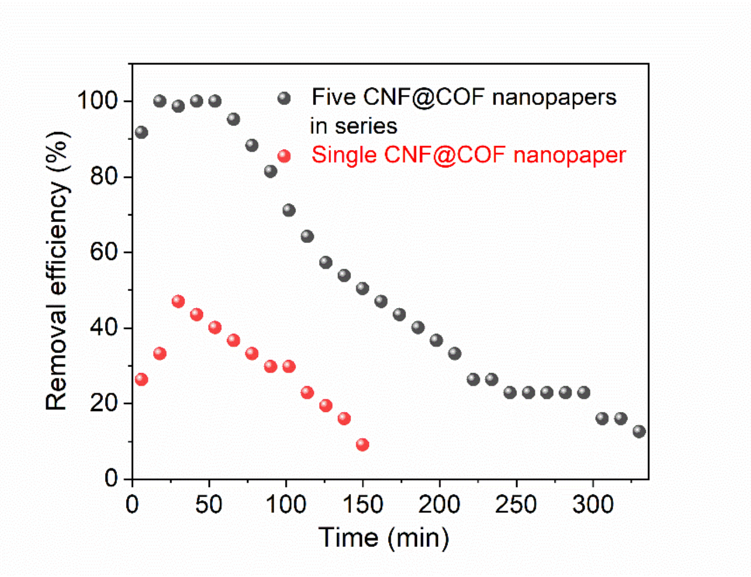

Figure S47. Removal efficient of single CNF@TFB-TAPB COF nanopaper and five CNF@TFB-TAPB COF nanopapers in series for 2 ppm ofloxacin**.**

Table S4. A comparison of aqueous synthesis methods and conditions for imine-linked COFs in this study and relevant literature.

Method	Synthesis conditions			Results					Ref.
	Catalyst	Temperature (°C)	Reaction time	Crystallinity	BET surface area ($m^2 g^{-1}$)	Yield (%)	Gram-level Synthesis	Space time yield ($g\ h^{-1}\ L^{-1}$)	
Hydrothermal synthesis in autoclaves	5.8 M aqueous acetic acid	120	3 days	High	302-1140	70-80	Yes	0.214 (TpPa-1)	6
Aqueous synthesis by stirring	5% aqueous acetic acid	35 or 80	120 h	High	800	90 or 97	No	0.005 (TFB-TAPB COF)	7
Mechanochemical synthesis and solid state reaction	Solid PTSA	120 or 90	3 or 12 h	high	593-2023	85	No	-	8
Reaction in sealed autoclaves with pressurized CO_2 at 4.5 MPa	H_2CO_3	Room temperature	24 h	High	678	82	No	0.9 (TFB-DB COF)	9
Sonochemical synthesis. Probe ultrasonication is needed.	6-12 M aqueous acetic acid	Ambient temperature generated by ultrasonication	1 h	High	940-2059	56-99	No	21.5 (TPB-TAPB COF)	10
Sonochemical synthesis. Probe ultrasonication is needed.	6-12 M aqueous acetic acid	Ambient temperature generated by ultrasonication	1 h	High	34-3054	32-99	No	11.8 (TFB-DB COF)	11
Aqueous synthesis by stirring	4-13 M aqueous acetic acid	Room temperature	2-72 h	High	482-1994	58-92	Yes	15.5 (TFB-TAPB COF, 2h) 9.5 (TFB-DB COF, 2h)	This work

Table S5. Acetic acid concentrations used in the synthesis of various imine-linked COFs.

	TFB-BD COF	TFB-DB COF	TFB-DDB COF	TFB-TAPA COF
The concentration of acetic acid (M)	4.38	4.38	8.75	4.38
	TFB-TAPB COF	TFB-ETTA COF	TPA-TAPM COF	TPA-TAPPA COF
The concentration of acetic acid (M)	4.38	4.38	8.75	13.2
	DMTA-TAPT COF	DMTA-PTTA COF	TFPA-TAPB COF	TFPB-TAPA COF
The concentration of acetic acid (M)	4.38	4.38	8.75	8.75
	TFPB-TAPB COF	TFPB-TAPT COF	TFPB-ETTA COF	TFPT-ETTA COF
The concentration of acetic acid (M)	8.75	4.38	8.75	8.75

Table S6. Comparison of synthesis conditions and properties between imine-linked COFs in this study and in the literature

Imine-linked COF	Reaction temperature	Solvent	Reaction time (h)	Crystallinity	BET surface area ($m^2 g^{-1}$)	Yield (%)	Morphology	Ref.
TFB-DB COF	Room temperature	Water	2	high	1015	87.8	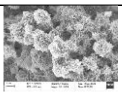	This work
	Room temperature	Water	72	high	944	75.5	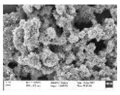	This work
	Ambient temperature under probe sonication	Water	1	high	1003	73	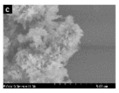	[11]
	25 °C	Organic solvents	48	high	1523	78	-	[12]
	120 °C	Organic solvents	72	High	410	90	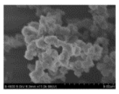	[2]
TFB-BD COF	Room temperature	Water	72	high	482	87	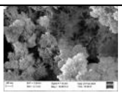	This work
	Ambient temperature under probe sonication	Water	1	high	449	93	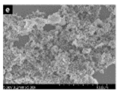	[11]
	80 °C	Organic solvents	72	high	1142	76	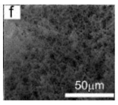	[13]
TFB-DDB COF	Room temperature	Water	72	high	459	58	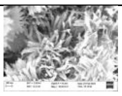	This work
	120 °C	Organic solvents	72	high	787	81	-	[14]
TFB-TAPA COF	Room temperature	Water	72	high	848	90	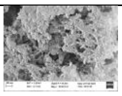	This work
	Ambient temperature under probe sonication	Water	1	high	866	90	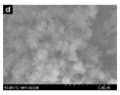	[11]
	120 °C	Organic solvents	168	high	680	92	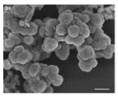	[15]
TFB-TAPB COF	Room temperature	Water	2	high	771	83.7	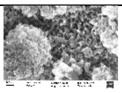	This work
	Room temperature	Water	72	high	1415	80	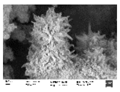	This work
	Ambient temperature under probe sonication	Water	1	high	1013	90	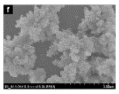	[11]
	80 °C	Water	120	high	806	90	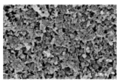	[7]
	120 °C	Organic solvents	168	high	1268	94	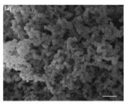	[6]

TFB-ETTA COF	Room temperature	Water	72	high	737	81	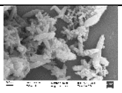	This work
	Ambient temperature under probe sonication	Water	1	high	940	86	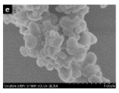	11
	120 °C	Organic solvents	72	high	895	-	-	16
TPA-TAPPA COF	Room temperature	Water	72	high	923	72	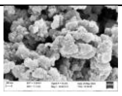	This work
	Sonochemical synthesis	Water	1	high	318	70	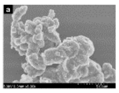	11
	100 °C	Organic solvents	72	high	1509	-	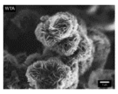	17
DMTA-TAPT COF	Room temperature	Water	72	high	1994	73	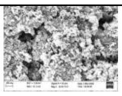	This work
	Ambient temperature under probe sonication	Water	1	high	1890	60	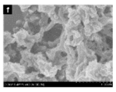	10
	Room temperature	Organic solvents	2	high	2068	93.1	-	18
DMTA-PTTA COF	Room temperature	Water	72	high	847	83	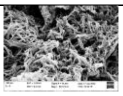	This work
	Ambient temperature under probe sonication	Water	1	high	1103	63	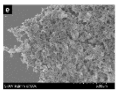	11
	120 °C	Organic solvents	120	high	1183	91	-	19
TFPA-TAPB COF	Room temperature	Water	72	high	1070	74	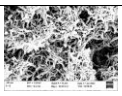	This work
	Ambient temperature under probe sonication	Water	1	high	645	50	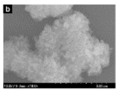	11
	120 °C	Organic solvents	72	high	2290	-	-	20
TFPB-TAPA COF	Room temperature	Water	72	high	617	73	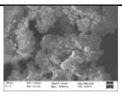	This work
	120 °C	Organic solvents	72	high	798	89.4	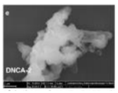	21
TFPB-TAPB COF	Room temperature	Water	72	high	2487	82	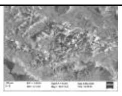	This work
	120 °C	Organic solvents	72	high	519	87.6	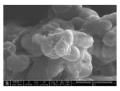	22
TFPB-TAPT COF	Room temperature	Water	72	high	1468	92	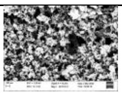	This work

	120 °C	Organic solvents	72	high	747	89.7	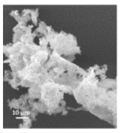	23
TFPB-ETTA COF	Room temperature	Water	72	high	1010	65	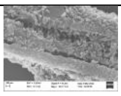	This work
	120 °C	Organic solvents	72	high	1174	80	-	24
TFPT-ETTA COF	Room temperature	Water	72	high	965	81	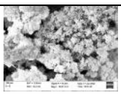	This work
	Ambient temperature under probe sonication	Water	1	high	984	67	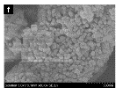	10
TPA-TAPM COF	Room temperature	Water	72	high	305	88	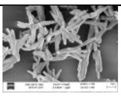	This work
	90 °C	Organic solvents	48	high	20	89	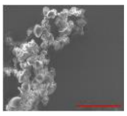	25 (hydrated form of COF-300)

Table S7. Comparison of CNF@COFs nanopaper presented in this study with reported COF-cellulose composites

Composites	Crystallinity	S_{BET} (m^2 g^{-1})	Freestanding	Foldability	Tensile strength (MPa)	Young's moduli (GPa)	Ref.
CNF@COFs nanopaper	high	205-549	yes	yes	40.2	2.6	This work
Bacterial Cellulose/COF-366	weak	37.81	yes	no	4.73	-	[26]
CF-COF nanopaper	weak	100.3	yes	yes	1.84	0.07	[27]
TamDbta-COF-Cellulose acetate membrane	high	104 (TamDbta-COF)	yes	no	-	-	[28]

Note: The reported COF-cellulose composites were fabricated through the physical mixing of COF nanoparticles or nanosheets with cellulose, while our study develops an interfacial synthesis approach for growing COF nanolayers on the surface of CNFs, leading to the fabrication of freestanding and mechanically stable CNF@COF nanopapers. The CNF@COF nanopapers demonstrated superior flexibility, enhanced mechanical strength, and higher porosity compared to those of COF-cellulose composites. This underscores the advantages of the developed interfacial synthesis strategy for the fabrication of COF-cellulose composites.

References

(1) Zhou, S.; Stromme, M.; Xu, C., Highly Transparent, Flexible, and Mechanically Strong Nanopapers of Cellulose Nanofibers @Metal-Organic Frameworks. *Chem. Eur. J.* **2019**, 25 (14), 3515-3520.

(2) Ding, S. Y.; Gao, J.; Wang, Q.; Zhang, Y.; Song, W. G.; Su, C. Y.; Wang, W., Construction of covalent organic framework for catalysis: Pd/COF-LZU1 in Suzuki-Miyaura coupling reaction. *J. Am. Chem. Soc.* **2011**, 133 (49), 19816-19822.

(3) Li, X.; Qiao, J.; Chee, S. W.; Xu, H. S.; Zhao, X.; Choi, H. S.; Yu, W.; Quek, S. Y.; Mirsaidov, U.; Loh, K. P., Rapid, Scalable Construction of Highly Crystalline Acylhydrazone Two-Dimensional Covalent Organic Frameworks via Dipole-Induced Antiparallel Stacking. *J. Am. Chem. Soc.* **2020**, 142 (10), 4932-4943.

(4) Uribe-Romo F. J.; Hunt J. R.; Furukawa H.; Klöck C.; O'Keeffe M.; Yaghi O. M., A Crystalline Imine-Linked 3-D Porous Covalent Organic Framework. *J. Am. Chem. Soc.* **2009**, 131 (13), 4570–4571

(5) Ma T.; Kapustin E. A.; Yin S. X.; Liang L.; Zhou Z.; Niu J.; Li L. H.; Wang Y.; Su J.; Li J.; Wang X.; Wang D. W.; Wang W.; Sun J.; Yaghi O. M., Single-Crystal X-ray Diffraction Structures of Covalent Organic Frameworks. *Science* **2018**, 361,48–52.

(6) Jayshri, T.; Harshitha, B. A.; Raya, R. K.; Sharath, K.; Bishnu P. B. ; Digambar, B. S. ; Neha, C.R. and Rahul, B., Constructing Covalent Organic Frameworks in Water via Dynamic Covalent Bonding. *IUCrJ* **2016**, *3*, 402–407.

(7) Martin-Illan, J. A.; Rodriguez-San-Miguel, D.; Franco, C.; Imaz, I.; Maspoch, D.; Puigmarti-Luis, J.; Zamora, F., Green Synthesis of Imine-Based Covalent Organic Frameworks in Water. *Chem. Commun.* **2020,** *56* (49), 6704-6707.

(8) Arjun, H.; Suvendu, K.; Matthew, A.; Saibal, B.; Amit, C.; Shebeeb, H. K.; Pradip, P.; Thomas, H.; and Rahul, B., Ultrastable Imine-based Covalent Organic Frameworks for Sulfuric Acid Recovery: An Effect of Interlayer Hydrogen Bonding. *Angew. Chem. Int. Ed.* **2018**, *57*,5797–5802.

(9) Fanyu, Z.; Jianling, Z.; Bingxing, Z.; Xiunithe, T.; Dan, S.; Jinbiao, S.; Dongxiog, T., Life L., Jiaqi F., Buxing H., Guanying Y., Lirong Z., and Jing Z., Room-Temperature Synthesis of Covalent Organic Framework (COF-LZU1) Nanobarsin CO2/Water Solvent, *ChemSusChem* **2018**,*11*,3576 –3580.

(10) Zhao, W.; Yan, P.; Yang, H.; Bahri, M.; James, A. M.; Chen, H.; Liu, L.; Li, B.; Pang, Z.; Clowes, R.; Browning, N. D.; Ward, J. W.; Wu, Y.; Cooper, A. I., Using Sound to Synthesize Covalent Organic Frameworks in Water. *Nat. Synth.* **2022,** *1* (1), 87-95.

(11) Zhao, W.; Yan, P.; Li, B.; Bahri, M.; Liu, L.; Zhou, X.; Clowes, R.; Browning, N. D.; Wu, Y.; Ward, J. W.; Cooper, A. I., Accelerated Synthesis and Discovery of Covalent Organic Framework Photocatalysts for Hydrogen Peroxide Production. *J. Am. Chem. Soc.* **2022,** *144* (22), 9902-9909.

(12) Shiraki, T.; Kim, G.; Nakashima, N., Room-temperature Synthesis of a Covalent Organic Framework with Enhanced Surface Area and Thermal Stability and Application to Nitrogen-doped Graphite Synthesis. *Chem. Lett.* **2015,** *44* (11), 1488-1490.

(13) Zhu, D.; Zhu, Y.; Yan, Q.; Barnes, M.; Liu, F.; Yu, P.; Tseng, C.-P.; Tjahjono, N.; Huang, P.-C.; Rahman, M. M.; Egap, E.; Ajayan, P. M.; Verduzco, R., Pure Crystalline Covalent Organic Framework Aerogels. *Chem. Mater.* **2021,** *33* (11), 4216-4224.

(14) Zhao, Y.; Xu, C.; Qi, Q.; Qiu, J.; Li, Z.; Wang, H.; Wang, J., Tailoring Delicate Pore Environment of 2D Covalent Organic Frameworks for Selective Palladium Recovery. *Chem. Eng. J.* **2022,** *446*.

(15) Li, X.; Gao, Q.; Aneesh, J.; Xu, H.-S.; Chen, Z.; Tang, W.; Liu, C.; Shi, X.; Adarsh, K. V.; Lu, Y.; Loh, K. P., Molecular Engineering of Bandgaps in Covalent Organic Frameworks. *Chem. Mater.* **2018,** *30* (16), 5743-5749.

(16) Nguyen, H. L.; Hanikel, N.; Lyle, S. J.; Zhu, C.; Proserpio, D. M.; Yaghi, O. M., A Porous Covalent Organic Framework with Voided Square Grid Topology for Atmospheric Water Harvesting. *J. Am. Chem. Soc.* **2020,** *142* (5), 2218-2221.

(17) Rotter, J. M.; Guntermann, R.; Auth, M.; Mahringer, A.; Sperlich, A.; Dyakonov, V.; Medina, D. D.; Bein, T., Highly Conducting Wurster-Type Twisted Covalent Organic Frameworks. *Chem. Sci.* **2020,** *11* (47), 12843-12853.

(18) Zhu, D.; Zhang, Z.; Alemany, L. B.; Li, Y.; Nnorom, N.; Barnes, M.; Khalil, S.; Rahman, M. M.; Ajayan, P. M.; Verduzco, R., Rapid, Ambient Temperature Synthesis of Imine Covalent Organic Frameworks Catalyzed by Transition-Metal Nitrates. *Chem. Mater.* **2021,** *33* (9), 3394-3400.

(19) Guo, M.; Jayakumar, S.; Luo, M.; Kong, X.; Li, C.; Li, H.; Chen, J.; Yang, Q., The Promotion Effect of pi-pi Interactions in Pd NPs Catalysed Selective Hydrogenation. *Nat. Commun.* **2022,** *13* (1), 1770.

(20) Xie, Y.; Pan, T.; Lei, Q.; Chen, C.; Dong, X.; Yuan, Y.; Maksoud, W. A.; Zhao, L.; Cavallo, L.; Pinnau, I.; Han, Y., Efficient and Simultaneous Capture of Iodine and Methyl Iodide Achieved by a Covalent Organic Framework. *Nat. Commun.* **2022,** *13* (1), 2878.

(21) Yang, J.; Ghosh, S.; Roeser, J.; Acharjya, A.; Penschke, C.; Tsutsui, Y.; Rabeah, J.; Wang, T.; Djoko Tameu, S. Y.; Ye, M. Y.; Gruneberg, J.; Li, S.; Li, C.; Schomacker, R.; Van De Krol, R.; Seki, S.; Saalfrank, P.; Thomas, A., Constitutional Isomerism of the Linkages in Donor-Acceptor Covalent Organic Frameworks and Its Impact on Photocatalysis. *Nat. Commun.* **2022,** *13* (1), 6317.

(22) Ma, D. L.; Qi, Q. Y.; Lu, J.; Xiang, M. H.; Jia, C.; Lu, B. Y.; Jiang, G. F.; Zhao, X., Transformation between 2D Covalent Organic Frameworks with Distinct Pore Hierarchy via Exchange of Building Blocks with Different Symmetries. *Chem. Commun.* **2020,** *56* (98), 15418-15421.

(23) Yang, J.; Acharjya, A.; Ye, M. Y.; Rabeah, J.; Li, S.; Kochovski, Z.; Youk, S.; Roeser, J.; Gruneberg, J.; Penschke, C.; Schwarze, M.; Wang, T.; Lu, Y.; van de Krol, R.; Oschatz, M.; Schomacker, R.; Saalfrank, P.; Thomas, A., Protonated Imine-Linked Covalent Organic Frameworks for Photocatalytic Hydrogen Evolution. *Angew. Chem. Int. Ed.* **2021,** *60* (36), 19797-19803.

(24) Lan, Y.; Han, X.; Tong, M.; Huang, H.; Yang, Q.; Liu, D.; Zhao, X.; Zhong, C., Materials Genomics Methods for High-Throughput Construction of COFs and Targeted Synthesis. *Nat. Commun.* **2018,** *9* (1), 5274.

(25) Fischbach D.M., Rhoades G., Espy C., Goldberg F., Smith B.J., Controlling the Crystalline Structure of Imine-Linked 3D Covalent Organic Framework. *Chem. Commun.*, **2019**, 55, 3594-3597**.**

(26) Falak S.; Shin B.; Kang C.; Khan Z. A.; Huh D. O., Novel Capturer-Catalyst Microreactor System with a Polypyrrole/Metal Nanoparticle Composite Incorporated in the Porous Honeycomb-Patterned Film. *ACS Appl. Mater. Interfaces* 2023, 15, 32903−32915.

(27) Xu Q.; Du X. H.; Luo D.; Strømme M.; Zhang Q. F.; Xu C., Gold Recovery from E-waste Using Freestanding Nanopapers of Cellulose and Ionic Covalent Organic Frameworks. *Chem. Eng. J.* **2023**, 458, 141498.

(28) Mohammed A. K.; Ali J. K.; Kuzhimully M. B. S.; Addicoat M. A.; Varghese S.; Baias M.; Alhseinat E.; Shetty D., The Fragmented 3D-Covalent Organic Framework in Cellulose Acetate Membrane for efficient Phenol Removal. *Chem. Eng. J.* **2023**, 466, 143234.
